# Comprehensive proteomic analysis of human cervical-vaginal fluid using colposcopy samples

**DOI:** 10.1186/1477-5956-7-17

**Published:** 2009-04-17

**Authors:** Geert Zegels, Geert AA Van Raemdonck, Edmond P Coen, Wiebren AA Tjalma, Xaveer WM Van Ostade

**Affiliations:** 1 Laboratory for Functional Proteomics, University of Antwerp, Universiteitsplein 1, 2610 Antwerp, Belgium; 2 Department of Gynecology and Gynecologic Oncology, University Hospital Antwerp, Wilrijkstraat 10, 2650 Antwerp, Belgium

## Abstract

**Background:**

Cervical-vaginal fluid (CVF) plays an important role in the prevention of gynecological infections, although little is known about the contribution of CVF proteins to the immunity of the lower female genital tract. In order to analyze the protein composition of human CVF, we used CVF samples that are routinely collected during colposcopy, but are usually discarded. Since these samples are available in large quantities we aimed to analyze their usefulness for proteomics experiments. The samples were analyzed using different prefractionation techniques (ultrafiltration and C_4_(RP)-LC protein separation) followed by C_18_(RP)-LC peptide separation and identification by MALDI-TOF-TOF mass spectrometry. To determine the reproducibility of this proteomics platform we analyzed three technical replicates. Using spectral counting, protein abundances were estimated in a semiquantitative way. We also compared the results obtained in this study with those from previous studies derived from patients with different physiological conditions in order to determine an overlapping protein set.

**Results:**

In total, we were able to identify 339 proteins in human CVF of which 151 proteins were not identified in any other proteomics study on human CVF so far. Those included antimicrobial peptides, such as human beta-defensin 2 and cathelicidin, which were known to be present in CVF, and endometrial proteins such as glycodelin and ribonucleoprotein A. Comparison of our results with previously published data led to the identification of a common protein set of 136 proteins. This overlapping protein set shows increased fractions of immunological and extracellular proteins, confirming the extracellular immunological role of CVF.

**Conclusion:**

We demonstrated here that CVF colposcopy samples can be used in proteomics experiments and hence are applicable for biomarker discovery experiments. The delineation of an overlapping set of proteins that is identified in most proteomics studies on CVF may help in the description of a reference proteome when performing proteomics studies on human CVF.

## Background

The female lower genital tract (vagina and ectocervix) is exposed to a large microbial pressure, whereby pathogens can invade via the mucosa or the epithelial layer. These microorganisms can cause infections and diseases which can lead to preterm birth, increased susceptibility to sexually transmitted diseases, infertility and cancer[1,2]. However, despite the frequent contact with pathogenic microorganisms, the incidence of infections is relatively low, suggesting that the female genital tract has developed numerous defense mechanisms against potential pathogens. Of these, the constant removal of adherent bacteria by shedding epithelial cells and the hydration of the cervical-vaginal mucosa by excretions from cervical and vaginal glands and by plasma transudate are believed to be most effective, yet these mechanisms are only partially understood. These actions lead to the formation of a biological fluid in the ectocervix and vaginal region, called the cervical-vaginal fluid (CVF) [1-6]. In addition, the vaginal and ectocervical mucosa is covered by numerous commensal bacteria, such as *Lactobacillus *spp., which produce organic acids and compete with exogenous bacteria for nutrients[1,2,5,7-9]. The adaptive immunity of the lower female genital tract mainly constitutes of T-lymphocytes present in the lamina propria of the cervix, Langerhans cells in the cervicovaginal mucosa and plasma cells in the close vicinity of submucosal glands producing secretory immunoglobulin A (sIgA) and IgG[1,10]. Recently, Tang *et al*.[11] suggested the presence of neutrophils and eosinophils on the basis of neutrophil and eosinophil granule secretion proteins, present in the human cervical-vaginal fluid. The innate immunity of the mucosa of the female lower genital tract has antimicrobial proteins/peptides (AMPs) (e.g. defensins, lactoferrin, cathelicidin, lysozyme, SLPI, etc.) as predominant effector molecules, which are present in cervicovaginal mucosal and glandular excretions (reviewed in[1]). Although structurally diverse, they are often small (< 100 amino acids), cationic and amphipathic molecules[12]. AMPs exert antimicrobial activity by 1) sequestration of microbial nutrients[13,14], 2) disruption of microbial structural proteins and membranes [14-16] and 3) preventing microbial adhesion on the mucosa[14,17]. Aside from these activities, they also have effects on the host's immunity[12] and on the target cells of viruses and bacteria [18-20].

It has been frequently demonstrated that proteomic analysis of body fluids can yield information for biomarker discovery and treatment development[21]. CVF samples are especially interesting in terms of gynecological diagnostics since these samples can easily be collected using non-invasive methods. Although conventional biomarkers are often quantified in plasma samples, there are two reasons why CVF samples are preferred over plasma samples in terms of gynecological biomarker discovery. Firstly, since the volume of plasma (± 3 liters) is much larger than e.g. vaginal washings (CVF + washing fluid = ± 50 ml) it could be expected that dilution of a (potential) biomarker will be much lower in the latter fluid. Secondly, altered biomarker expression patterns in plasma are often not very specific as they may be associated with different pathologies because plasma comes in contact with all organs of the body. In contrast, when using CVF samples, it is expected that expression patterns will directly correlate with gynecological pathologies[22].

The first large proteomics study on the CVF proteome was performed by Gravett *et al*. on Rhesus monkeys[23]. In addition, seven studies analyzed peptides and proteins present in human CVF using antibody-independent proteomics techniques[11,24-29]. The studies of Venkataraman *et al*.[29], which focused on the cationic protein and peptide fraction, and Di Quinzio *et al*.[26], which used 2D-PAGE to analyze common spots of the obtained gels from different CVF samples, were limited to the identification of subfractions of the CVF proteome. The other five studies attempted to catalogue the whole CVF-proteome[11,24,25,27,28] but differed in patient physiology (healthy, infected, pregnant, etc.) and sample preparation, separation and identification methods. Mutual comparison of these studies shows there is a large variation in protein identifications. This is not surprising given the fact that CVF is a body fluid that can be influenced by many biological factors including menstruation, age, infection, sexual intercourse, usage of contraceptives, pregnancy, etc. Also, the different studies on human CVF used diverse sample collection and analytical methods to analyze the CVF proteome, which may contribute to the large variation. We therefore hypothesise that the CVF proteome consists of 1) a fixed set of proteins ("core proteome") which is usually present in the extracellular cervical-vaginal region independent of the condition of the patient and applied experimental setups and 2) a variable set of proteins whose abundance is dependent on (combinations of) several of the abovementioned physiological and experimental factors. These arguments point to the CVF as a highly variable body fluid where normalization will be an absolute prerequisite in considering the use of these samples for biomarker identification and for diagnosis/follow-up of gynecological diseases.

To further explore the CVF proteome, we combined C_4_(RP)-LC on protein level followed by C_18_(RP)-LC on peptide level with MALDI-TOF-TOF mass spectrometry for protein identification. The main goal of the study was to further complete the protein list of the CVF proteome by analyzing new types of samples (routinely collected samples that are available in high quantities; see below) rather than measuring variability. We noticed however that one part of our protein list consisted of newly identified proteins, while another part comprised proteins that were frequently identified in previous studies. This last set ("overlapping protein set") could be considered as a subset of the human CVF "core proteome".

## Methods

### Sample collection

Low-grade squamous intraepithelial lesions (LSIL) are considered to be a benign cytological effect of human papilloma virus (HPV) replication. However, since 20% of these individuals progress to high-grade squamous intraepithelial lesions (HSIL)[30], a stage that precedes cervical cancer, these women are routinely checked for progression to HSIL by colposcopy, a procedure that requires washing of the vagina with 5% acetic acid. The lavage fluid is usually discarded but was used by us since these samples are routinely collected and hence are available in large quantities. As such, we wished to examine whether they could serve other diagnostic and/or proteomics purposes too (e.g. early diagnosis of cervical cancer). Therefore, CVF samples were collected from seven women with LSIL at the department of gynecology of the University Hospital of Antwerp (UZA). All patients (37–45 years old) included in this study were either in the first or second half of the menstrual cycle, but were not menstruating at time of sample collection, were not pregnant, did not use any contraceptive and had not had sexual intercourse less than 48 hours prior to sample collection. The cervicovagina was washed with 50 ml of 5% acetic acid for 2 minutes, the lavage fluid containing the CVF was collected (15–30 ml), immediately transported to the laboratory and stored at -80°C. Patients agreed to participate by written consent.

### Sample preparation and ultrafiltration

**Figure 1:**
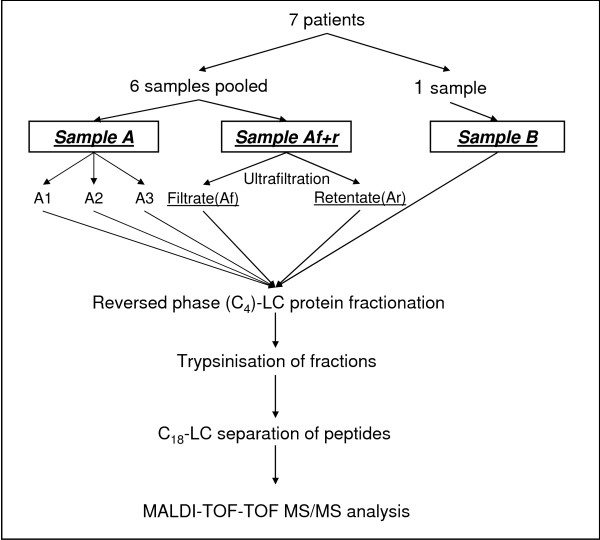
**Overview of the different workflows used**. 7 samples were divided over 3 experimental setups using different fractionation techniques (ultrafiltration and reversed phase (C_4_)-LC protein fractionation). All processes ended with a C_18_-LC separation on peptide level and MALDI-TOF-TOF MS/MS analysis.

Six samples were pooled (together sample A; 191 ml; 128 μg/ml) and one sample (sample B; 16 ml; 111 μg/ml) (fig. 1) was not pooled prior to analysis. Sample A was divided into two fractions: A (not ultrafiltrated; 31 ml; 128 μg/ml) and Af+r (which was later ultrafiltrated; 160 ml; 128 μg/ml). In order to improve the recovery of the low molecular weight fraction (LMWF) from human CVF during ultrafiltration, acetonitril (ACN) was added to sample Af+r to a final concentration of 20%[31]. All samples (A, Af+r and B) were then centrifuged for 150 minutes at 4°C at 10000 g to obtain a clear pellet and the supernatant was collected. Sample Af+r was then applied onto Centriplus^® ^centrifugal filter devices (Amicon, Millipore, Bedford, MA, USA) with a molecular weight cut-off of 30 kDa and filtered according to the manufacturer's guidelines. Unfiltrated samples A and B, the filtrate (sample Af; 108 ml; 18 μg/ml) and the retentate (sample Ar; 8 ml; 1780 μg/ml) of sample Af+r were lyophilized to near dryness. Afterwards, sample A (0.5 ml; 7960 μg/ml) was divided into three fractions (samples A1, A2 and A3) in order to determine the technical variation of the used analytic LC-MS platform. Protein concentrations were determined using the Bradford method.

### Reversed phase (C_4_)-LC protein fractionation

Samples A1-3, Af (0.5 ml; 3300 μg/ml), Ar (1.0 ml; 9133 μg/ml) and B (0.5 ml; 4910 μg/ml) were fractionated on a RP Protein C_4 _VYDAC HPLC column (214TP5415; 4.6 × 150 mm, particle size 5 μm; Alltech Associates Inc., Lokeren, Belgium) using a Waters™ 600S controller, a Waters™ 626 pump and a Waters™ 996 PDA (Waters Corporation, Milford, MA, USA). Solvent A was 0.1% TFA in water and solvent B 0.1% TFA in ACN. 1 mg of protein was loaded on the column and an ACN gradient was applied which differed between samples. In all cases the flowrate used for the chromatography was 1 ml/min. For unfiltrated samples A1-3, and B the following gradient was used: 5 minutes 3%B, 3%B to 60%B over 15 minutes, 60%B to 100%B over 2 minutes, 2 minutes 100%B and 100%B to 3%B over 3 minutes. For the sample Ar (which contained the larger proteins): 4 minutes 3%B, 3%B to 25%B over 1 minute, 25%B to 60%B over 15 minutes, 60%B to 100%B over 2 minutes, 2 minutes 100%B and 100%B to 3%B over 3 minutes. For sample Af (which contained smaller proteins): 5 minutes 3%B, 3%B to 40%B over 15 minutes, 40%B to 100%B over 3 minutes, 2 minutes 100%B and 100%B to 3%B over 3 minutes. In all cases, 16 fractions were collected every 1.5 minutes from minute 3 to minute 27. Fractions were lyophilized to dryness and protein content of the fractions was determined based upon the chromatogram surface.

### Sample digestion

All fractions were resuspended in 50 mM TRIS.HCl/6 M urea/5 mm dithiothreitol/10% beta-mercaptoethanol pH 8.5 (25 μl/100 μg protein). Because a minimum amount of material is required to perform a reproducible trypsin digest, fraction 1 was pooled with fractions 10–14, resulting in 9 fractions for further analysis. Each fraction was incubated for 1 h at 65°C for denaturation and reduction. Samples were diluted with 50 mM TRIS.HCl/1 mM CaCl_2 _(75 μl/100 μg protein) and alkylated by adding 200 mM iodoacetamide (10 μl/100 μg protein; 1 h at 21°C in dark). Proteomics-grade modified trypsin (Roche, Manheim, Germany) was added in a 30:1 protein-to-enzyme ratio and fractions were incubated for 18 h at 37°C. Digestion was stopped by freezing (-80°C).

### Microcapillar HPLC separation of peptides and MALDI-target spotting

One dimensional peptide separations were performed on an Agilent 1100 series Micro-Capillary HPLC system (Agilent Technologies, Waldbronn, Germany). Digested fractions from sample A1-3, Af, Ar and B were injected at a flowrate of 6 μl/min using a Rheodyne (Cotati, CA, USA) 9725 manual injection valve, connected with the capillary pump, on a Zorbax 300SB-C_18 _guard column (0.3 mm × 5 mm; particle size 5 μm; Agilent Technologies) serially connected with a Zorbax 300SB-C_18 _analytical reverse-phase column (0.3 mm × 150 mm; particle size 3.5 μm; Agilent Technologies). The solvents used were 0.1% formic acid (FA) in water (solvent A) and 0.1%FA/90%ACN (solvent B). Columns were equilibrated with 3% solvent B. Using the capillary pump, an ACN gradient was applied (flowrate 6 μl/min unchanged over the chromatographic run): 5% to 55% in 56.7 min, ramp to 90%B over 3.3 min, 90%B for 5 min, 85%B for 5 min and back to equilibrating conditions after the end of the run. During the chromatographic separation, 350 spots (800 nl/spot) were collected on Opti-TOF^® ^MALDI-targets (28 columns × 25 rows; 700 spots; 2 runs/target) (part number: 1018469; Applied Biosystems, Inc., Foster City, CA, USA) with an 8 second interval starting from minute 5 to minute 51.7 of the run. Thereafter, matrix (5 mg/ml α-cyanohydroxy cinnamic acid in 70% ACN; internal calibrant: 63 pmol/ml human [Glu^1^]-fibrinopeptide B) was added to the collected spots using an external syringe pump. A flow-rate of 6 μl/min was applied and matrix was spotted with an 8 second interval (800 nl matrix/spot) until all spots were covered with matrix.

### MALDI-TOF-TOF analysis

Spots from samples A1-3, Af, Ar and B were analyzed using an AB4800 proteomics analyzer (Applied Biosystems) first by MALDI-TOF (reflectron mode; 25 × 20 lasershots per spot; mass-range: 700–2500 Da; laser intensity: 2500) and precursors with a signal-to-noise (S/N) ratio above or equal to 35 were selected for MALDI-TOF-TOF (MS/MS) analysis. A maximum of 50 unique precursors per spot was selected for fragmentation, starting from the precursor with the lowest S/N-ratio. Selected precursors were ionized (25 × 20 lasershots per spot; laser intensity: 4000) and fragmented in a collision cell (1 kV collisions (positive mode) with air).

### Data analysis

#### Protein identification

Spectra obtained from the different samples (A1-3, Af, Ar and B) were sent to the MASCOT search engine (version 2.1.03; ) using the GPS Explorer Software (Applied Biosystems) and screened against the Swissprot database specified for *Homo sapiens *(version: Sprot_55.2). Carbamidomethylation of cystein was entered as a fixed modification, while oxidation of methionine was entered as a variable modification. Up to two missed trypsin cleavages were tolerated. The mass tolerance for the precursors was 50 ppm and 0.25 Da for the fragment ions. The MudPIT scoring algorithm was used.

Since proteins that are part of the LMWF often result in only a few or one detectable tryptic peptide, protein identifications with one unique peptide were allowed and accepted, however only under certain stringent conditions. In general, only the top-ranking peptides (for more information see ) were used for identification, the difference between the calculated and experimental peptide-mass had to be larger than -0.1 and less than 0.1 and the obtained spectra, used for identification, had to fulfil the criteria for high quality spectra (see below)[32]. Beside those, additional criteria used were: 1) Proteins with a MASCOT-score that corresponded with p-value < 0.01 were considered identified if there was at least one unique peptide, used for the identification, with a peptide-score above or equal to the threshold score and with a length of at least 7 amino acids. 2) Protein identifications with a MASCOT-score that corresponded with a p-value between 0.05–0.01 were manually validated. Here, peptides used for identification were to meet the following criteria[32]: peptides had to be at least 7 amino acids long, the peptide-score had to be above or equal to the threshold score and the spectra had to contain at least 3 consecutive b-and/or y-ions. The partially identified sequences were then submitted to "BLAST protein-protein"  and screened against the *Homo sapiens *Swissprot database to see if this identification matched the MASCOT-identification. All keratins were removed from the list.

In order to analyze whether the applied identification methodology described above was stringent enough, we estimated the false discovery rate on the protein level (FDR). Therefore, spectra were screened against a concatenated database consisting of the target Swissprot database (*Homo sapiens*) and a shuffled (decoy) Swissprot database (*Homo sapiens*). FDR was calculated as described: 2× false positive identifications/(false positive identifications + true positive identifications)[33]. In all cases, the FDR on the protein level had to be less than 5% before we assumed that the obtained results were trustworthy.

#### Functional and cellular component classification of proteins

Functional classification of proteins was achieved using a multi-staged classification methodology based upon four different databases: 1) The "PANTHER" classification system [34,35], 2) Gene Ontology [36], 3) the "DAVID" database [37] and 4) Swissprot (manual annotation of proteins not classified using the other three databases). Proteins which remained unclassified after applying the four abovementioned tools were placed in the "not determined" (ND) category. Classification of proteins according to their cellular localization was achieved using a similar method as used for the functional classification, with the exception that the "PANTHER" classification system was not used. Again, unclassified proteins were placed in the ND category.

#### Semiquantitative analysis of proteins

To semiquantitatively estimate the abundance of a protein in a mixture, we determined the total count of MS/MS spectra for each detected protein [38-41]. To correct the determined spectral count for differences in protein size, we normalized by dividing the number of counted spectra through the number of predicted observable peptides [40-42]. These observable peptides are obtained after *in silico *trypsinization of the protein. However, due to some technical restrictions, not all theoretical tryptic peptides can be identified. Therefore, the theoretical tryptic peptides were filtered according to their mass (mass-range mass spectrometer: 700–2500 Da) and we estimated retention time (C_18_(RP)-LC eluate collection window: from minute 5 to 51.7) using the "Sequence Specific Retention Calculator" version 3.0[43]. We then calculated the Normalized Spectral Abundance Factor (NSAF)[41], which is directly proportional to the molar concentration, as follows (equation 1):



Where *SC *is the number of spectral counts for protein *k*, *OP *the number of observable peptides (after filtering) and *N *the total number of proteins identified in one experiment. For convenience we multiplied these NSAF-values by a factor of 1000. We also calculated a "normalized spectral count factor" (NSCF), which does not take into account the number of observable peptides, and normalized those data by dividing the number of spectral counts for protein *k *through the summation of the spectral counts of all identified proteins *N *(equation 2).



Again, we multiplied these NSCF-values by a factor 1000 for convenience. This NSCF-value is directly proportional to the mass concentration rather then molar concentration[44].

### Comparison of the obtained data with previous studies using a relational database

For the comparison of the different proteomics studies on CVF, a relational database was constructed in Microsoft^® ^Office Access 2003 around the data presented in the articles[11,23-29] and our study. Before creating the final input lists for the database, we manually examined all data sets and corrected for redundancies since we noticed that in some cases the same proteins were entered more than once or different proteins were identified by the same set of peptides. Each study was entered in a table containing a specific study number and the Swissprot accession numbers of the identified proteins. These tables were linked to a table with the study specifications and a table with the corresponding protein descriptions. The latter was further linked with tables containing specific details about the identified proteins (functional classification, cellular component classification, MW, pI, etc.; additional file 1).

## Results

### Protein identification from human cervical-vaginal fluid

Since it is well described that usage of different fractionation techniques enhances the dynamic range of the experimental setup and increases proteome coverage[45], we analyzed the effect of the incorporation of an ultrafiltration (cut-off 30 kDa) step, prior to the C_4_(reversed-phase (RP))-LC fractionation on the protein level, on the number of protein identifications obtained (fig. 1). Every experimental setup ended in C_18_(RP)-LC peptide separation and MALDI-TOF-TOF mass spectrometric analysis.

Table 1 shows the number of spectra collected during MS-analysis of the different samples. Also shown is the number of proteins identified by Mascot with a score corresponding to a p-value of < 0.05 (second column). A further manual screening of this protein list was performed since proteins often represented several isoforms or protein subsets that could not be identified on the unique peptide level. Isoforms of a particular protein were only taken up in the final protein list when it was possible to identify with a high degree of confidence the peptide which contained the discriminating sequence. We also noticed during this validation that many identifications were based upon low scoring peptides which did not fulfill the criteria described in the Methods section (see above) and hence were left out. The last column therefore shows the number of proteins which were considered identified after applying the manual data-analysis and after removal of known contaminants such as keratins. As can be seen, using these stringent criteria, about half of the proteins from the original list passed this screening. We believe this stringent screening is necessary in order to be able to positively identify small proteins and peptides on the base of only one unique peptide.

**Table 1:** Overview of the number of spectra collected and MASCOT search results before and after manual data inspection.

	***# spectra collected***	***# proteins identified before*** ***manual data-analysis***	***# proteins identified after *** ***manual data-analysis***
***Sample A1***	6351	312	147
***Sample A2***	10514	424	201
***Sample A3***	9063	364	179
***Sample Af***	7630	194	85
***Sample Ar***	9711	329	164
***Sample B***	6880	210	129

For each sample the number of spectra collected (column 1), the number of proteins which were identified by Mascot with a score which corresponded with a p-value < 0.05, before (column 2) and after (column 3) our manual data analysis, are presented.

In order to estimate the FDR of the applied identification methodology, spectra from each experimental setup were screened against a concatenated database consisting of a forward (target) Swissprot database and a shuffled (decoy) database. FDR estimations were calculated as described[33] and were as follows: 2.7% (sample A1), 2.0% (sample A2), 2.2% (sample A3), 2.0% (sample Af), 2.2% (sample Ar) and 1.4% (sample B).

Table 2 lists the number of proteins identified in the different experiments and shows the overlapping numbers. All together, we were able to identify 339 proteins from human CVF with high confidence. Complete lists of identifications and their corresponding score from the different experiments are presented in the supporting information (additional file 2). 151 (45%) out of those 339 proteins had not been identified in any other previous study (additional file 3).

**Table 2:** Overview of the number of proteins identified in the different experimental setups showing the interexperimental overlap.

***Sample:***	***A1***	***A2***	***A3***	***A1-3***	***Af***	***Ar***	***Af+r***	***B***
***A1***	**147**							
***A2***	130	**201**						
***A3***	126	152	**178**					
***A1-3***	147	201	178	**237**				
***Af***	48	53	50	55	**85**			
***Ar***	87	103	93	109	49	**164**		
***Af+r***	94	112	100	118	85	164	**200**	
***B***	87	95	91	103	48	79	84	**129**

We then classified the proteins according to the biological process in which they exert their role and their cellular localization. We noticed that CVF consists of a mixture of proteins with a wide variety of functions. The largest categories are formed by proteins which have a function in protein metabolism and modification (19%), immunity and defense (13%), developmental process (9%) and signal transduction (9%). Aside from these, several smaller functional categories were also represented (additional file 4). Using the multi-staged classification process (see Methods) we could reduce the unclassified category from 17% (PANTHER alone) to 2%. Classification according to their cellular localization showed us that identified proteins were most likely to be present in the cytoplasm (26%), extracellular region (25%), membrane (12%) and cytoskeleton (11%). The complete cellular component classification is presented in additional file 5.

### Analysis of the technical variation of the applied LC-MS platform

**Figure 2:**
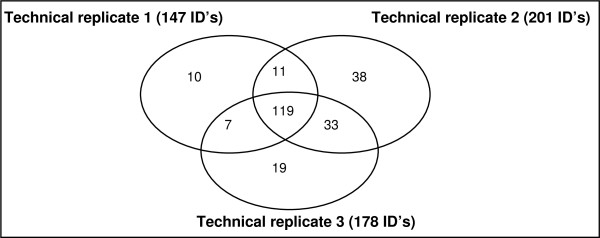
**Overlap of protein identifications between three technical replicates**.

To determine the technical variation of the used LC-MS platform, we analyzed a pool of CVF (sample A) in triplicate. After centrifugation and lyophilization, sample A was divided into three fractions (sample A1, A2 and A3) which were separately analyzed in triplicate. The Venn-diagram illustrating the number of proteins found in each technical replicate and the overlap between the triplicates are shown in figure 2. In total, 237 different proteins were identified in the three technical replicates, of which 119 were common to all three replicates. To correctly calculate the percentage of shared versus total number of identifications we made use of the calculation of Hattan *et al*[46]. that takes into account the redundancy of identifications. The three technical replicates therefore yielded 526 (147+201+178) identifications of which 357 (3*119) were found in all three replicates. This results in a ratio of 68% (357/526).

### Estimation of protein abundance

We estimated protein abundances of the identified proteins using the spectral counting method and calculated the NSAF-value (equation 1). The spectral counting information of all proteins identified in the different experiments is given in the additional information (additional file 6). The results show clearly that protein S100A9 is the most abundant protein present in human CVF.

**Figure 3:**
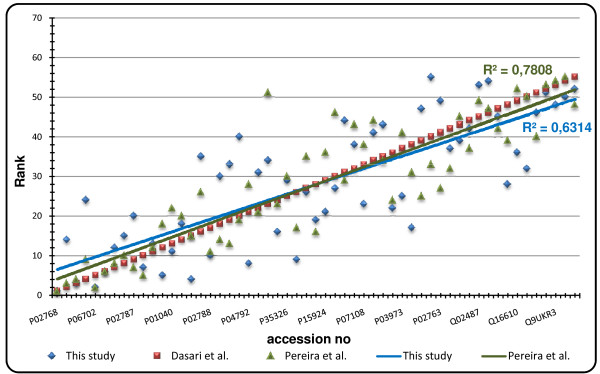
**Comparison of spectral counting information from different studies**.

We next compared our spectral counting information with results from other studies on human CVF[24,27]. In order to achieve this, we selected from the common 119 proteins (see above), those which were also present in the studies from Dasari *et al*.[24] and Pereira *et al*.[27]. This resulted in 67 proteins. For a correct comparison, average NSCF values over the three technical replicates for these proteins were calculated after which proteins were ranked according to their abundancy (1 = most abundant; 67 = least abundant). Proteins were then sorted on the basis of the ranking from Dasari *et al*. and plotted as protein (x-axis) vs. rank (y-axis). After removal of six outliers, best-fit plots were calculated showing very similar trends in all of the three studies (fig. 3).

### Delineation of an overlapping protein set that is shared by various CVF proteomics studies

Comparison of our results with those from others [11,24,26-29] showed a very high variability in protein identifications between different studies. However, we reasoned that if one considers the CVF "core proteome" as a set of proteins that is present in theoretically every CVF sample, then these proteins must appear in the majority of CVF proteomics studies, even when these studies make use of samples originating from patients under different physiological conditions. Although the determination of the complete CVF "core proteome" lies beyond the scope of this study, an overlapping protein set which is shared by diverse proteomics studies and which is therefore irrespective of sampling and analytical methods as well as patient physiology, may very well represent a subset of this "core proteome" and hence may be a first step in delineating it. We therefore constructed a relational database (additional file 1) consisting of our data combined with data from all proteomics studies on CVF[11,24-29].

**Figure 4:**
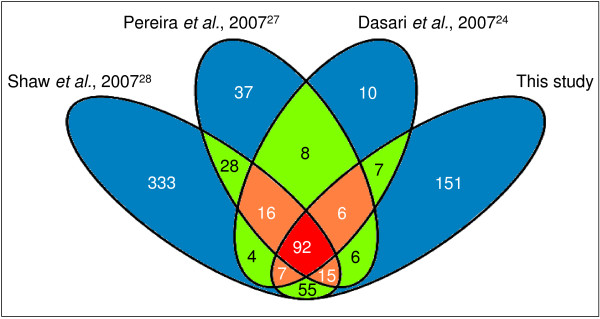
**Overlap of protein identifications between the four most comprehensive studies on CVF**. Orange and red areas contain proteins which are present in at least 3 out of 4 studies and were included in the overlapping protein set (136 proteins).

Thus far, a total of 826 proteins were identified from the CVF proteome (complete list, overlapping proteins (additional file 7), and functional (additional file 8) and cellular component (additional file 9) classification of those proteins can be found in the additional information. Using this database, we extracted an overlapping protein set from the three most comprehensive studies[24,27,28] (all with > 150 identifications) and our results (fig. 4). In total, 136 proteins (listed in additional file 10 and 11) were present in at least 3 out of 4 studies, of which 92 were present in all four studies. As can be seen in table 3, these studies made use of samples coming from patients with different profiles. Moreover, the samples underwent various experimental procedures, indicating that this overlapping protein set can be identified, independent of patient physiology or applied analytical methods.

**Table 3:** Overview of studies performed on human CVF.

***Study***	***Samples***	***Part of sample analyzed***	***Separation method***	***MS method***
Venkataraman *et al*., 2005[29]	Undiluted CVF collected in cup from healthy women (postmenarcheal, pre-menopausal)	Cationic fraction	2D-PAGE (1D: AU-PAGE; 2D: Tricine- SDS-PAGE)	MALDI-TOF-TOF

Di Quinzio *et al*., 2007[26]	Swabs from pregnant women (37 weeks gestation)	Only protein spots identified common to five gels	2D-PAGE (1^st^D: IEF; 2^nd^D SDS-PAGE) followed by RP-LC	MALDI-TOF or ESI-linear IT

Dasari *et al*., 2007[24]	Swabs from pregnant women (18,5 weeks gestation as mean)	Whole CVF	1D-SDS-PAGE followed by offline 2D(SCX/RP)-LC	ESI-Q-TOF

Tang *et al*., 2007[11]	Washings from clinically normal women; 7 washings from women infected with *Candida *spp.	Whole CVF	2D-PAGE (1^st^D: IEF; 2^nd^D SDS-PAGE)	MALDI-TOF-TOF

Shaw *et al*., 2007[28]	Gauze from healthy women	Whole CVF	1D-SDS-PAGE or SCX-LC both followed by RP-LC	ESI-linear IT

Pereira *et al*., 2007[27]	Swabs from pregnant women (15.8–35.9 weeks gestation)	Whole CVF	2D-DIGE or MudPIT(SCX/RP)-LC	ESI-Q-TOF

Klein *et al*., 2008[25]	Swabs from pregnant women (30.5 weeks gestation as mean)	Whole CVF	RP-LC	ESI-IT

This study	Washings from HPV-infected women	Whole CVF	Ultrafiltration or C_4_-LC protein fractionation/C_18_-LC peptide separation	MALDI-TOF-TOF

For each study the following information is presented: 1) the nature of the samples which were used in the study (sample collection method and patient physiology), 2) whether the study focused on a particular part of the sample or analyzed the whole CVF, 3) the methods used to separate proteins and peptides and 4) MS method used for analysis.

**Figure 5:**
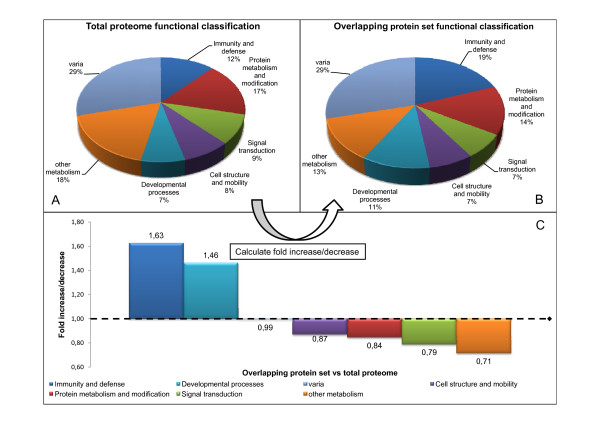
**Fold changes of functional categories between the total proteome and the overlapping protein set**. The total proteome (A) and the overlapping protein set (B) were classified into functional categories and the fold change of the different functional classes between these two sets was calculated as follows: percentage of a specific category of the overlapping protein set divided by the percentage of that category in the total proteome (C).

**Figure 6:**
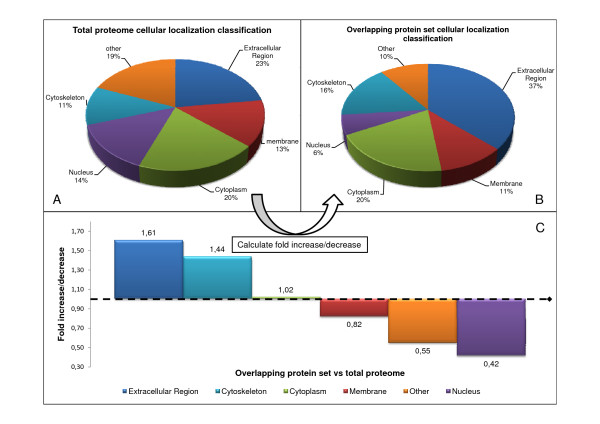
**Fold changes of cellular localization categories between the total proteome and the overlapping protein set**. The total proteome (A) and the overlapping protein set (B) were classified according to the cellular localization (respectively A en B) and the fold change of the different functional classes between these two sets was calculated as follows: percentage of a specific category of the overlapping protein set divided by the percentage of that category in the total proteome (C).

We then compared the proportion (in percentage) of the functional and cellular component categories from this overlapping protein set (respectively fig. 5B and 6B) with those from the total CVF proteome, consisting of all 826 proteins (respectively fig. 5A and 6A), and calculated the fold change (increase/decrease) between these two sets (fig. 5C and 6C). The calculation was done as follows: percentage of a specific category of the overlapping protein set divided by the percentage of that category in the total proteome. Hence, a fold change > 1 indicates an increase of a particular category in the overlapping protein set. Highly variable categories (i.e. categories consisting of proteins which differ a lot between different studies) will lead to a fold change < 1, since only a small fraction of this category will be identified in more studies. Figure 5C shows an increase of the "immunity and defense" and the "developmental processes" category in the overlapping protein set, whereas all other functional categories decrease.

Similarly, figure 6C shows the fold change in cellular localization classification. Here, two categories ("extracellular region" and "cytoskeleton") were markedly increased in the overlapping protein set.

## Discussion

### Evaluation of different experimental setups for human CVF proteomics analysis

In order to obtain a maximal coverage of the CVF proteome, we evaluated the effect of enhanced sample fractionation on identification yield (fig. 1).

An average of 175 proteins was identified in the three technical replicates (samples A1-3; no ultrafiltration + C_4_(RP)-LC). This number is lower than the 200 identifications obtained from samples Af+r (ultrafiltration + C_4_(RP)-LC), suggesting that the setup with the highest degree of prefractionation (sample Af+r) still results in the largest number of identifications. However, although it was expected that extended prefractionation drastically reduces sample complexity resulting in an enhancement of proteome coverage and dynamic range of the used method[45], the effect of an additional ultrafiltration is only modest. Moreover, filtrate (Af) and retentate (Ar) showed a significant overlap (49 proteins), suggesting that ultrafiltration does not result in a precise separation, as also suggested in other studies[47]. Therefore, supplementary prefractionation on the protein level, such as SCX-LC or SDS-PAGE, may prove more useful, although these procedures suffer from an increased workload and sample consumption.

When comparing the pooled samples A1-3 (technical replicates) with the single sample B (which was not part of the pool; table 2), 80% of the proteins from sample B were also found in samples A1-3. This preliminary comparison points to a significant overlap between samples from different patients. Further studies with more samples are required to ascertain this (Van Raemdonck *et al*., in preparation).

Since 339 protein identifications is clearly above the average number as compared to previous publications, and because a large fraction of these proteins is frequently found in other studies too (see below), we conclude that the samples resulting from the colposcopy of patients with LSIL may well be used for proteomics studies, e.g. for biomarker discovery.

### Analysis of the technical variation of the applied LC-MS platform

119 identifications were found in all of the three technical replicates (samples A1, A2 and A3), which is an overlap of 68% between these replicates. Moreover, 89% of all peptides were used to identify these 119 overlapping proteins.

These results are very similar in terms of technical variation and reproducibility as compared to other studies which analyzed technical replicates based on multidimensional LC-MS proteome analysis platforms, such as those from Liu *et al*.[44] and Shaw *et al*.[28] (respectively 60% and 70% overlap between technical replicates).

It has been stated that, for complex protein mixtures, a multidimensional chromatographic separation is reasonably reproducible, whereas the acquisition of MS/MS spectra (and therefore also the identification of proteins) shows much less reproducibility[48]. This assumption was confirmed by the analysis of the reproducibility of our LC-system based on the comparison of retention times derived from 63 peaks extracted from the chromatograms of three technical replicates. We determined an average coefficient of variance of 0.56% meaning that the chromatography is very reproducible. As such, these results suggest that the largest variation of the platform can probably be found at the level of the mass spectrometric analyses. We assume that a certain degree of random selection of precursor-ions, the masking of low abundant peptides by higher abundant ones[44] and small day-to-day variations in sensitivity and accuracy may result in variable MS/MS spectra influencing the reproducibility of the platform[49].

### Characterization of the CVF proteome

A substantial number of identifications concerned proteins with a serine protease function (e.g. kallikrein-6/10/11/13/14, transmembrane serine proteases 11D/11E, leukocyte elastase and myeloblastin) and other proteases (e.g. cathepsin G). Also, inhibitors of serine proteases (serpin B3/B4/B12/B13, calpastatin, SLPI, alpha-1 antitrypsin, serine protease inhibitor Kazal-type 7/5, plasma serine protease inhibitor), inhibitors of cystein proteases (calpastatin, cystatin A/B) and inhibitors of other proteases (SLPI, WAP four-disulfide core domain protein 2) were found. Many of these serve an immunological function (beside other functions) and some are described to originate from neutrophils (elastase, cathepsin G). Indeed, the presence of polymorphonuclear leucocytes in CVF was recently demonstrated by Tang *et al*.[11]. Other immune peptides and proteins (e.g. defensins, lactoferrin, immunoglobulins, azurocidin, myeloperoxidase, TLR-7, IL-17 ...) were also identified of which cathelicidin (sample A1-3, Af+r and B) and human beta-defensin 2 (HBD-2; sample A1-3 and B) were of special interest. These antimicrobial peptides have not been identified in any other proteomics study on CVF so far[11,24-29]. Frohm *et al*. used immunohistochemistry to show that cathelicidin is expressed by the squamous epithelium of the cervix and vagina[50]. Also, the group of Valore *et al*. analyzed the concentration of several AMP's in CVF using ELISA and determined the concentration of HBD-2 (0.57 ± 0.13 μg/ml)[5]. Remarkably, although the measured concentration of human neutrophil peptide (HNP; alpha-defensin) (0.35 ± 0.07 μg/ml) was slightly lower than HBD-2[5], almost every proteomics study on human CVF was able to identify HNP, but not HBD-2[24,27-29], thereby raising the question as to why it is so difficult to identify HBD in CVF samples. We presume that the reason for this may be found in the higher arginine and lysine content of the cathelicidin propeptide (19%; one observable peptide) and HBD-2 (18%; three observable peptides) compared to HNP (12%; 5 observable peptides) which leaves, after trypsinization, only very few peptides that are large enough for identification by MS.

A large portion of the identified proteins, including involucrin, small proline rich proteins, cystatin A and desmosomal proteins, are components of the cornified envelope, a protein/lipid structure situated just below the epithelial cell membrane[51]. At the final stage of epithelial cell differentiation, this envelope resides on the exterior of the dead cornified cells, so that many of its elements may come off and could be detected in the CVF. This process is probably stimulated by the subsequent changes in estrogen and progesterone levels during menstruation, which causes cyclic histological changes in the cervical-vaginal epithelium[52].

Of particular interest were some endometrial proteins including glycodelin (picked up for the first time in CVF), a protein involved in regulation of the uterine environment[53], and heterogeneous nuclear ribonucleoprotein A which is thought to serve a role in the formation of specific myometrial proteins[54]. We also identified proteins such as mucin 5B which is an important component of secreted mucus[55] and carbonic anhydrase 1/2 which is involved in the formation of aqueous humor[56].

25% of the identified proteins were present in the extracellular compartment, which was to be expected since samples concerned an extracellular fluid. By comparison, the studies of Dasari *et al*.[24], Pereira *et al*.[27] and Tang *et al*.[11] identified about 39% extracellular proteins. All of the abovementioned studies[11,24,27], including ours, removed cells from their samples by centrifugation. The use of 5% acetic acid as colposcopy washing buffer in our study may have caused additional cellular lysis before centrifugation of the samples. Nonetheless, in absolute numbers, 83 proteins were classified in the extracellular region which is more than in any of the three abovementioned studies[11,24,27] (respectively 22, 58 and 79). We therefore conclude that the colposcopy samples have the intrinsic disadvantage of increased cellular lysis but this is compensated for by the proteomics platform described here.

In general, intracellular proteins are abundantly identified in every study. This may have different reasons. A certain number of lysed cells may always be present in CVF because of the natural loss of epithelial cells in the course of the menstrual cycle[52]. Also, all patients were infected with HPV which leads to a weakening of the cornified envelope resulting in fragile cells[57,58] that are more easily lysed upon mechanical stress. Intracellular proteins may also have an extracellular function, such as histones (which were also identified in this study) that are secreted or are part of the "neutrophil extracellular traps" (long extensions built out of chromatine and elastase and which are able to bind and inhibit bacteria and fungi [59-61]). Furthermore, it has been shown that exosomes (nanovesicles which are secreted and carry MHC and adhesion molecules on their surfaces and contain cytosolic enzymes and proteins) can be secreted by epithelial cells and B cells, although this has not yet been described for the cervical-vaginal mucosa[62,63]. Finally, intracellular proteins may be actively secreted to fulfill an as yet unknown extracellular physiological function.

### Estimation of protein abundance

We used the semiquantitative spectral counting method and calculated the NSAF-value to analyze abundances of proteins from three replicate samples [40-42] (additional file 6). Many highly abundant proteins were extracellular proteins with an immunological function such as protein S100A9 and S100A8, cystatin A/B, antileukoproteinase, immunoglobins and elafin. Serum albumin and hemoglobin alpha/beta were also abundant probably due to plasma transudate.

Abundancy ranking of the 61 overlapping proteins between our study and those from Dasari *et al*.[24] and Pereira *et al*.[27] showed a very similar trend in all studies. The slightly better fit between the latter two was not unexpected because both studies were carried out by the same research group and thus the sample collection method, patient physiology and LC-MS method were quite similar (table 3). Nevertheless, we conclude that the relative abundancy of the proteins identified in our study is similar to other studies even when other sample collection and LC-MS methods are used.

### Delineation of an overlapping protein set that is shared by various CVF proteomics studies

As mentioned before, we speculate that the CVF proteome consists of two large parts: 1) a fixed set of proteins ("core proteome") of which the composition does not vary and which is usually present in the cervical-vaginal region irrespective of patient condition and the analytical methods used and 2) a variable set of proteins from which abundance is dependent on several physiological and experimental factors. In fact, a similar situation has been seen in human plasma where a substantial fraction of identifications from previous research papers (between 13 and 35%) wherein different sample preparations and identification technologies were used, matched with a core dataset established by the human plasma project[64]. We compared the three most comprehensive CVF proteomics studies [24,27,28] with our results and found an overlapping protein set of 136 proteins. 120 proteins (35%) from the 339 identified in our study were part of this overlapping protein set (including 101 and 73 proteins from samples A and B, respectively). These 120 proteins have very divergent NSAF-values (from 32 to 2021; additional file 6) indicating that they are present in different concentrations in the CVF and are not always highly abundant. Also, the majority (60%, 80%, 87% and 94%) of the proteins identified in the studies of respectively Tang *et al*.[11], Venkataraman *et al*.[29], Di Quinzio *et al*.[26] and Klein *et al*.[25] were present in this overlapping protein set, confirming its mutual nature.

We then calculated the fold increase/decrease (in percentage) of functional and cellular localization categories (fig. 5 and fig. 6 respectively) in the overlapping protein set as compared to the total set of proteins (826) identified in all of the studies[11,24-29]. From this, we noticed significant increases of the "immunity and defense" and "developmental processes" functional categories and of the "extracellular region" and "cytoskeleton" cellular localization categories. An increase of the "immunity and defense" category was not unexpected since cervical-vaginal immunity is an indispensable factor in preventing infections of the lower female genital tract (see introduction). Indeed, proteins such as HNP, SLPI, lysozyme C, azurocidin, etc. were frequently detected; many of them even by all four studies (additional files 10 and 11). Since all studies made use of samples of cervical-vaginal fluid, and because many proteins from the innate immunity system are present in the extracellular region, it was expected that the portion of this latter category would also increase in the overlapping protein set. The increases in the "developmental process" and "cytoskeleton" categories were less likely to occur. However, a closer look shows that a large fraction (both 50%) of these categories consisted of proteins that are part of the cornified envelope (e.g. small proline rich proteins, periplakin, cystatin A, S100 proteins, desmoplakin, enveloplakin, annexin I, etc). As mentioned before, since the lower female genital tract is lined with a differentiated epithelial cell layer, the cornified envelope is well developed[51] and parts of it may be shed in the CVF.

We conclude that the use of colposcopy samples, combined with the LC-MS platform described here, results in a relatively high number of CVF protein identifications (extracellular as well as intracellular). However, we are aware of the fact that, due to the presence of acetic acid in the colposcopy sample solution, local cell lysis may occur, leading to the delivery of unwanted intracellular proteins in the CVF. However, the comparison of our results with other studies wherein cell lysis was minimized (Dasari *et al*.[24] and Pereira *et al*.[27]) allowed us to distinguish between correct and aberrant identifications. This is because we expect identifications from unwanted intracellular proteins to be more random. As such, these proteins will not be picked up frequently and the chance of them falling within the overlapping protein set will be drastically reduced. Indeed, during determination of the overlapping protein set, many intracellular proteins from all of the studies were quickly filtered out since they did not occur in at least three out of the four studies. As an example, from the 40 nuclear proteins we identified in our study, only 8 remained in the overlapping set.

In our study, we were able to identify 151 proteins which were not identified in any other proteomics study on human CVF so far[11,24-29]. Many of these are probably the result of increased cellular lysis, due to the 5% acetic acid present in the sample. On the other hand, the high efficiency of the proteomics platform may have contributed to the unique identification of proteins which can be expected to reside in the CVF. For instance, HBD-2 and cathelicidin have never been identified in any previous proteomics study, yet ELISA experiments show that these peptides are definitely present in CVF[5,50]. One explanation for this high number could be that characterization of the CVF proteome has not reached saturation yet. Alternatively, we suggest that every comprehensive proteomics study on CVF will bring in proteins from the "core" as well as the "variable" protein set whereby the chance of picking up proteins from the former set is higher, hence the "core" set will reach saturation faster. One must keep in mind however, that not all proteins from the "core proteome" have an expression level that allows identification. Therefore, although this study gives an idea of the ratio between the "core" and the "variable" set in CVF samples, exhaustive lists of both proteomes must come from a collaborative effort whereby statistically relevant numbers of samples from well chosen patients are analyzed by several proteomics strategies.

## Conclusion

We showed here that routinely collected CVF colposcopy samples, which are usually discarded, can be used for proteomic studies on human CVF. In total, 339 proteins were identified using different experimental setups. Comparison with other studies suggests that there is a large variability in terms of protein composition in CVF samples. Therefore, two requirements need to be fulfilled when using CVF samples for biomarker discovery and validation experiments: first, high numbers of samples need to be analyzed in order to obtain statistically significant results. Since the colposcopy samples used here can easily be obtained in large quantities (samples from several hundreds of patients can be collected over one year from the colposcopy department) and from different centres, they are well suited for such extensive experiments. Second, the lack of a good internal standard makes comparison and quantitative analysis of CVF samples difficult. For this reason we determined an overlapping protein set which we consider to be a first step towards the delineation of a CVF "core proteome". Although more studies need to be executed on CVF samples before the final determination of this "core proteome" can be obtained, this proteome has likely a lower variability and may be proven useful as a reference proteome for normalization during analysis of the different samples with different proteomics platforms.

Finally, among the 339 proteins identified in this study, 151 were not previously identified in any proteomics study on human CVF. Among those were proteins which are present in the lower female genital tract, such as HBD-2 and cathelicidin, two proteins that play an important role in the innate immunity of the cervicovagina. All together, our results suggest that the colposcopy samples in combination with the proteomics platform applied here can be used for comprehensive proteomics studies on CVF.

## Abbreviations

CVF: cervical-vaginal fluid; AMP: antimicrobial peptides/proteins; PAGE: polyacrylamide gel electrophoresis; LC: liquid chromatography; MS: mass spectrometry; MALDI: matrix assisted laser desorption ionisation; TOF: time-of-flight; Q: quadrupole; ESI: electrospray ionisation; IT: ion trap; LSIL: low-grade squamous intraepithelial lesions; HPV: human papilloma virus; HSIL: high-grade squamous intraepithelial lesions; LMWF: low molecular weight fraction; ACN: acetonitril; FA: formic acid; FDR: false discovery rate; MW: molecular weight; RP: reversed phase; SLPI: secretory leukocyte peptidase inhibitor; HBD: human beta-defensin; HNP: human neutrophil peptide; ND: not determined; NSAF: normalized spectral abundance factor; NSCF; normalized spectral count factor; SC: spectral count; OP: observable peptides; MHC: major histocompatibility complex.

## Competing interests

The authors declare that they have no competing interests.

## Authors' contributions

GZ designed and executed all experiments, performed the data-analysis, constructed the relational database and wrote the manuscript. GAAVR was responsible for the execution and analysis of the technical replicates. EPC performed the C_4_(RP)-LC fractionation, provided advice and reviewed the manuscript. WAAT was responsible for the sample collection and provided advice. XWMVO was scientific lead and responsible for the experimental design, supervision and writing of the manuscript.

## Supplementary Material

Additional file 1 **Scheme of the relational database constructed for the comparison of different proteomics studies on CVF.** Click here for file

Additional file 2 **Overview of identifications with their corresponding MASCOT-scores obtained in the different experiments performed in our study.** Click here for file

Additional file 3 **Overview of the proteins which were uniquely identified in our study.** Click here for file

Additional file 4 **Functional classification of the identified proteins in this study.** Click here for file

Additional file 5 **Classification according to cellular localisation of the identified proteins in this study**. Click here for file

Additional file 6 **Overview of the identifications with their corresponding spectral counting information obtained in the different experiments performed in our study.** Click here for file

Additional file 7 **Overview of all identifications obtained in different human proteomics studies on human CVF.** Click here for file

Additional file 8 **Functional classification of all proteins identified in comprehensive CVF proteomics studies so far.** Click here for file

Additional file 9 **Classification according to cellular localisation of all proteins identified in comprehensive CVF proteomics studies so far.** Click here for file

Additional file 10 **Overview of proteins which were included in the overlapping protein set.** Click here for file

Additional file 11 **Classification of proteins which were included overlapping in the protein set.** Click here for file

## References

[B1] Cole AM (2006). Innate host defense of human vaginal and cervical mucosae. Curr Top Microbiol Immunol.

[B2] Quayle AJ (2002). The innate and early immune response to pathogen challenge in the female genital tract and the pivotal role of epithelial cells. J Reprod Immunol.

[B3] Eschenbach DA, Thwin SS, Patton DL, Hooton TM, Stapleton AE, Agnew K, Winter C, Meier A, Stamm WE (2000). Influence of the normal menstrual cycle on vaginal tissue, discharge, and microflora. Clin Infect Dis.

[B4] Kistner RW, Hafez ES, Evans TN (1978). Physiology of the vagina. The human vagina.

[B5] Valore EV, Park CH, Igreti SL, Ganz T (2002). Antimicrobial components of vaginal fluid. Am J Obstet Gynecol.

[B6] Wagner G, Levin RJ, Hafez ES, Evans TN (1978). Vaginal fluid. The human vagina.

[B7] Hillier SL, Holmes KK, Sparling PF, Mardh P, Lemon SM, Stamm WE, Piot P, Wasserheit WN (1999). Normal vaginal flora. Sexually transmitted diseases.

[B8] Reid G (2001). Probiotic agents to protect the urogenital tract against infection. Am J Clin Nutr.

[B9] Stanek R, Gain RE, Glover DD, Larsen B (1992). High performance ion exclusion chromatographic characterization of the vaginal organic acids in women with bacterial vaginosis. Biomed Chromatogr.

[B10] Meredith SD, Raphael GD, Baraniuk JN, Banks SM, Kaliner MA (1989). The pathophysiology of rhinitis. III. The control of IgG secretion. J Allergy Clin Immunol.

[B11] Tang LJ, De SF, Odreman F, Venge P, Piva C, Guaschino S, Garcia RC (2007). Proteomic analysis of human cervical-vaginal fluids. J Proteome Res.

[B12] Brown KL, Hancock RE (2006). Cationic host defense (antimicrobial) peptides. Curr Opin Immunol.

[B13] Striz I, Trebichavsky I (2004). Calprotectin – a pleiotropic molecule in acute and chronic inflammation. Physiol Res.

[B14] Strate BW van der, Beljaars L, Molema G, Harmsen MC, Meijer DK (2001). Antiviral activities of lactoferrin. Antiviral Res.

[B15] Lehrer RI, Barton A, Daher KA, Harwig SS, Ganz T, Selsted ME (1989). Interaction of human defensins with Escherichia coli. Mechanism of bactericidal activity. J Clin Invest.

[B16] Lonnerdal B (2003). Nutritional and physiologic significance of human milk proteins. Am J Clin Nutr.

[B17] Gallo SA, Wang W, Rawat SS, Jung G, Waring AJ, Cole AM, Lu H, Yan X, Daly NL, Craik DJ, Jiang S, Lehrer RI, Blumenthal R (2006). Theta-defensins prevent HIV-1 Env-mediated fusion by binding gp41 and blocking 6-helix bundle formation. J Biol Chem.

[B18] Chang TL, Francois F, Mosoian A, Klotman ME (2003). CAF-mediated human immunodeficiency virus (HIV) type 1 transcriptional inhibition is distinct from alpha-defensin-1 HIV inhibition. J Virol.

[B19] Chang TL, Vargas J, DelPortillo A, Klotman ME (2005). Dual role of alpha-defensin-1 in anti-HIV-1 innate immunity. J Clin Invest.

[B20] Quinones-Mateu ME, Lederman MM, Feng Z, Chakraborty B, Weber J, Rangel HR, Marotta ML, Mirza M, Jiang B, Kiser P, Medvik K, Sieg SF, Weinberg A (2003). Human epithelial beta-defensins 2 and 3 inhibit HIV-1 replication. AIDS.

[B21] Veenstra TD, Conrads TP, Hood BL, Avellino AM, Ellenbogen RG, Morrison RS (2005). Biomarkers: mining the biofluid proteome. Mol Cell Proteomics.

[B22] Good DM, Thongboonkerd V, Novak J, Bascands JL, Schanstra JP, Coon JJ, Dominiczak A, Mischak H (2007). Body fluid proteomics for biomarker discovery: lessons from the past hold the key to success in the future. J Proteome Res.

[B23] Gravett MG, Thomas A, Schneider KA, Reddy AP, Dasari S, Jacob T, Lu X, Rodland M, Pereira L, Sadowsky DW, Roberts CT, Novy MJ, Nagalla SR (2007). Proteomic Analysis of Cervical-Vaginal Fluid: Identification of Novel Biomarkers for Detection of Intra-amniotic Infection. J Proteome Res.

[B24] Dasari S, Pereira L, Reddy AP, Michaels JE, Lu X, Jacob T, Thomas A, Rodland M, Roberts CT, Gravett MG, Nagalla SR (2007). Comprehensive proteomic analysis of human cervical-vaginal fluid. J Proteome Res.

[B25] Klein LL, Jonscher KR, Heerwagen MJ, Gibbs RS, McManaman JL (2008). Shotgun proteomic analysis of vaginal fluid from women in late pregnancy. Reprod Sci.

[B26] DI Quinzio MK, Oliva K, Holdsworth SJ, Ayhan M, Walker SP, Rice GE, Georgiou HM, Permezel M (2007). Proteomic analysis and characterisation of human cervico-vaginal fluid proteins. Aust N Z J Obstet Gynaecol.

[B27] Pereira L, Reddy AP, Jacob T, Thomas A, Schneider KA, Dasari S, Lapidus JA, Lu X, Rodland M, Roberts CT, Gravett MG, Nagalla SR (2007). Identification of novel protein biomarkers of preterm birth in human cervical-vaginal fluid. J Proteome Res.

[B28] Shaw JL, Smith CR, Diamandis EP (2007). Proteomic analysis of human cervico-vaginal fluid. J Proteome Res.

[B29] Venkataraman N, Cole AL, Svoboda P, Pohl J, Cole AM (2005). Cationic polypeptides are required for anti-HIV-1 activity of human vaginal fluid. J Immunol.

[B30] Fey MC, Beal MW (2004). Role of human papilloma virus testing in cervical cancer prevention. J Midwifery Womens Health.

[B31] Tirumalai RS, Chan KC, Prieto DA, Issaq HJ, Conrads TP, Veenstra TD (2003). Characterization of the low molecular weight human serum proteome. Mol Cell Proteomics.

[B32] Adkins JN, Varnum SM, Auberry KJ, Moore RJ, Angell NH, Smith RD, Springer DL, Pounds JG (2002). Toward a human blood serum proteome: analysis by multidimensional separation coupled with mass spectrometry. Mol Cell Proteomics.

[B33] Elias JE, Gygi SP (2007). Target-decoy search strategy for increased confidence in large-scale protein identifications by mass spectrometry. Nat Methods.

[B34] Mi H, Guo N, Kejariwal A, Thomas PD (2007). PANTHER version 6: protein sequence and function evolution data with expanded representation of biological pathways. Nucleic Acids Res.

[B35] Thomas PD, Campbell MJ, Kejariwal A, Mi H, Karlak B, Daverman R, Diemer K, Muruganujan A, Narechania A (2003). PANTHER: a library of protein families and subfamilies indexed by function. Genome Res.

[B36] Ashburner M, Ball CA, Blake JA, Botstein D, Butler H, Cherry JM, Davis AP, Dolinski K, Dwight SS, Eppig JT, Harris MA, Hill DP, Issel-Tarver L, Kasarskis A, Lewis S, Matese JC, Richardson JE, Ringwald M, Rubin GM, Sherlock G (2000). Gene ontology: tool for the unification of biology. The Gene Ontology Consortium. Nat Genet.

[B37] Dennis G, Sherman BT, Hosack DA, Yang J, Gao W, Lane HC, Lempicki RA (2003). DAVID: Database for Annotation, Visualization, and Integrated Discovery. Genome Biol.

[B38] Old WM, Meyer-Arendt K, Aveline-Wolf L, Pierce KG, Mendoza A, Sevinsky JR, Resing KA, Ahn NG (2005). Comparison of label-free methods for quantifying human proteins by shotgun proteomics. Mol Cell Proteomics.

[B39] Zybailov B, Coleman MK, Florens L, Washburn MP (2005). Correlation of relative abundance ratios derived from peptide ion chromatograms and spectrum counting for quantitative proteomic analysis using stable isotope labeling. Anal Chem.

[B40] Ishihama Y, Oda Y, Tabata T, Sato T, Nagasu T, Rappsilber J, Mann M (2005). Exponentially modified protein abundance index (emPAI) for estimation of absolute protein amount in proteomics by the number of sequenced peptides per protein. Mol Cell Proteomics.

[B41] Zybailov BL, Florens L, Washburn MP (2007). Quantitative shotgun proteomics using a protease with broad specificity and normalized spectral abundance factors. Mol Biosyst.

[B42] Liu Q, Tan G, Levenkova N, Li T, Pugh EN, Rux JJ, Speicher DW, Pierce EA (2007). The proteome of the mouse photoreceptor sensory cilium complex. Mol Cell Proteomics.

[B43] Krokhin OV, Craig R, Spicer V, Ens W, Standing KG, Beavis RC, Wilkins JA (2004). An improved model for prediction of retention times of tryptic peptides in ion pair reversed-phase HPLC: its application to protein peptide mapping by off-line HPLC-MALDI MS. Mol Cell Proteomics.

[B44] Liu H, Sadygov RG, Yates JR (2004). A model for random sampling and estimation of relative protein abundance in shotgun proteomics. Anal Chem.

[B45] Qian WJ, Jacobs JM, Liu T, Camp DG, Smith RD (2006). Advances and Challenges in Liquid Chromatography-Mass Spectrometry-based Proteomics Profiling for Clinical Applications. Mol Cell Proteomics.

[B46] Hattan SJ, Marchese J, Khainovski N, Martin S, Juhasz P (2005). Comparative study of [Three] LC-MALDI workflows for the analysis of complex proteomic samples. J Proteome Res.

[B47] Georgiou HM, Rice GE, Baker MS (2001). Proteomic analysis of human plasma: failure of centrifugal ultrafiltration to remove albumin and other high molecular weight proteins. Proteomics.

[B48] Wolters DA, Washburn MP, Yates JR (2001). An automated multidimensional protein identification technology for shotgun proteomics. Anal Chem.

[B49] Berg M, Parbel A, Pettersen H, Fenyo D, Bjorkesten L (2006). Reproducibility of LC-MS-based protein identification. J Exp Bot.

[B50] Frohm NM, Sandstedt B, Sorensen O, Weber G, Borregaard N, Stahle-Backdahl M (1999). The human cationic antimicrobial protein (hCAP18), a peptide antibiotic, is widely expressed in human squamous epithelia and colocalizes with interleukin-6. Infect Immun.

[B51] Nemes Z, Steinert PM (1999). Bricks and mortar of the epidermal barrier. Exp Mol Med.

[B52] Sjoberg I, Cajander S, Rylander E (1988). Morphometric characteristics of the vaginal epithelium during the menstrual cycle. Gynecol Obstet Invest.

[B53] Seppala M, Taylor RN, Koistinen H, Koistinen R, Milgrom E (2002). Glycodelin: a major lipocalin protein of the reproductive axis with diverse actions in cell recognition and differentiation. Endocr Rev.

[B54] Pollard AJ, Sparey C, Robson SC, Krainer AR, Europe-Finner GN (2000). Spatio-temporal expression of the trans-acting splicing factors SF2/ASF and heterogeneous ribonuclear proteins A1/A1B in the myometrium of the pregnant human uterus: a molecular mechanism for regulating regional protein isoform expression in vivo. J Clin Endocrinol Metab.

[B55] Brunelli R, Papi M, Arcovito G, Bompiani A, Castagnola M, Parasassi T, Sampaolese B, Vincenzoni F, De SM (2007). Globular structure of human ovulatory cervical mucus. FASEB J.

[B56] Bar-Ilan A, Pessah NI, Maren TH (1984). The effects of carbonic anhydrase inhibitors on aqueous humor chemistry and dynamics. Invest Ophthalmol Vis Sci.

[B57] Lehr E, Jarnik M, Brown DR (2002). Human papillomavirus type 11 alters the transcription and expression of loricrin, the major cell envelope protein. Virology.

[B58] Lehr E, Hohl D, Huber M, Brown D (2004). Infection with Human Papillomavirus alters expression of the small proline rich proteins 2 and 3. J Med Virol.

[B59] Brinkmann V, Reichard U, Goosmann C, Fauler B, Uhlemann Y, Weiss DS, Weinrauch Y, Zychlinsky A (2004). Neutrophil extracellular traps kill bacteria. Science.

[B60] Buchanan JT, Simpson AJ, Aziz RK, Liu GY, Kristian SA, Kotb M, Feramisco J, Nizet V (2006). DNase expression allows the pathogen group A Streptococcus to escape killing in neutrophil extracellular traps. Curr Biol.

[B61] Urban CF, Reichard U, Brinkmann V, Zychlinsky A (2006). Neutrophil extracellular traps capture and kill Candida albicans yeast and hyphal forms. Cell Microbiol.

[B62] Clayton A, Turkes A, Navabi H, Mason MD, Tabi Z (2005). Induction of heat shock proteins in B-cell exosomes. J Cell Sci.

[B63] van Niel G, Raposo G, Candalh C, Boussac M, Hershberg R, Cerf-Bensussan N, Heyman M (2001). Intestinal epithelial cells secrete exosome-like vesicles. Gastroenterology.

[B64] Omenn GS, States DJ, Adamski M, Blackwell TW, Menon R, Hermjakob H, Apweiler R, Haab BB, Simpson RJ, Eddes JS, Kapp EA, Moritz RL, Chan DW, Rai AJ, Admon A, Aebersold R, Eng J, Hancock WS, Hefta SA, Meyer H, Paik YK, Yoo JS, Ping P, Pounds J, Adkins J, Qian X, Wang R, Wasinger V, Wu CY, Zhao X (2005). Overview of the HUPO Plasma Proteome Project: results from the pilot phase with 35 collaborating laboratories and multiple analytical groups, generating a core dataset of 3020 proteins and a publicly-available database. Proteomics.

