# Supplementary material for: Comprehensive proteomic analysis of human cervical-vaginal fluid using colposcopy samples
Source: Proteome Sci. 2009 Apr 17;7:17. doi: 10.1186/1477-5956-7-17 (PMC2678104; doi:10.1186/1477-5956-7-17)

Additional file 1 - Scheme of the relational database constructed for the comparison of different proteomics studies on CVF.

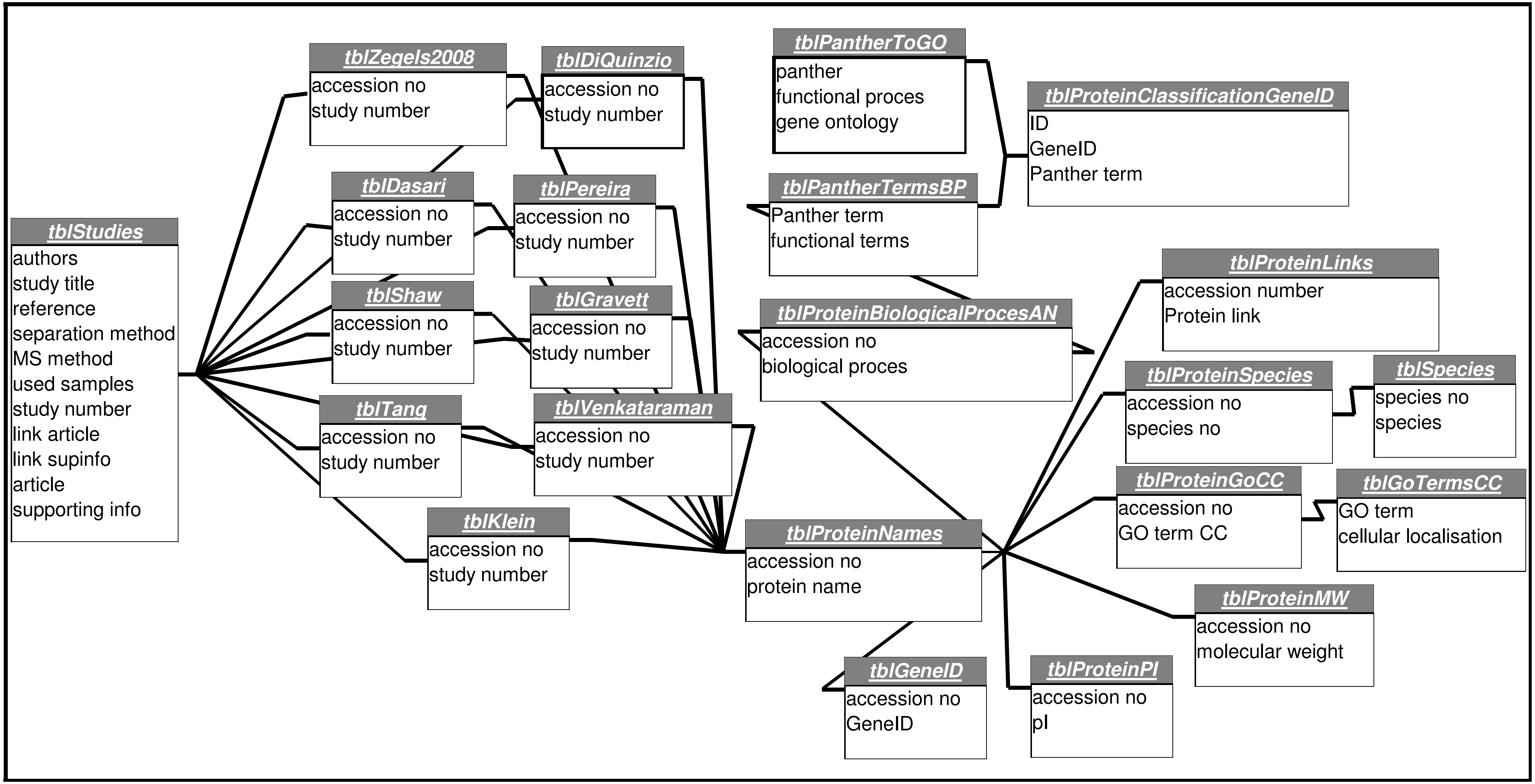

Supplement: Additional file 1 — Scheme of the relational database constructed for the comparison of different proteomics studies on CVF. [file 1477-5956-7-17-S1.pdf]
