# Supplementary material for: Comprehensive proteomic analysis of human cervical-vaginal fluid using colposcopy samples
Source: Proteome Sci. 2009 Apr 17;7:17. doi: 10.1186/1477-5956-7-17 (PMC2678104; doi:10.1186/1477-5956-7-17)
Supplement: Additional file 2 — Overview of identifications with their corresponding MASCOT-scores obtained in the different experiments performed in our study. [file 1477-5956-7-17-S2.pdf]

Additional file 2 – Overview of identifications with their corresponding MASCOT-scores obtained in the different experiments performed in our study.

Sample A1-3: six pooled samples, C_4 fractionated on protein level, C_{18} separation on peptide level, replicated 3 times. Sample Af and Ar: same samples pooled as sample A, filtrated (sample Af: filtrate; sample Ar: retentate), C_4 fractionated on protein level, C_{18} separation on peptide level. Sample B: one single sample, unpooled, C_4 fractionated on protein level, C_{18} separation on peptide level. Sample A1-3, Af, Ar and B: a score >32 corresponds with p<0,05.

Accession No	*Protein Description*	*Sample A1*	*Sample A2*	*Sample A3*	*Sample Af*	*Sample Ar*	*Sample B*
(1) A6NL28	Putative tropomyosin alpha-3 chain-like protein		57	40			
(2) A8K2U0	Alpha-2-macroglobulin-like protein 1 precursor		42	42		85	65
(3) A8MQ03	UPF0574 protein C9orf169		68				
(4) A9Z1Y9	Thymosin beta-4-like protein 6	84		110			
(5) O00555	Voltage-dependent P/Q-type calcium channel subunit alpha-1A				37		
(6) O15144	Actin-related protein 2/3 complex subunit 2		70	70			
(7) O15231	Zinc finger protein 185	103	118	99	72	38	
(8) O15263	Beta-defensin 2 precursor	41	53	57			43
(9) O43175	D-3-phosphoglycerate dehydrogenase					43	
(10) O43240	Kallikrein-10 precursor	295	266	367	57	172	45
(11) O43707	Actinin, alpha 4		45			94	
(12) O60235	Transmembrane protease, serine 11D precursor					80	89
(13) O60437	Periplakin	1541	2161	2010	100	922	362
(14) O60664	Mannose-6-phosphate receptor binding protein 1	42					
(15) O60814	Histone H2B type 1-K		363	405			266
(16) O75369	Filamin-B		42			40	
(17) O94823	Probable phospholipid-transporting ATPase VB						39
(18) O95171	Sciellin	150	282	289		122	188
(19) O95274	Ly6/PLAUR domain-containing protein 3 precursor	267	275	220		110	120
(20) P00338	L-lactate dehydrogenase A chain					117	
(21) P00441	Superoxide dismutase [Cu-Zn]		63				
(22) P00558	Phosphoglycerate kinase 1					215	
(23) P00738	Haptoglobin					32	33
(24) P00915	Carbonic anhydrase 1					41	
(25) P00918	Carbonic anhydrase 2					39	
(26) P01009	Alpha-1-antitrypsin precursor	174	160	152	61	35	51
(27) P01024	Complement component 3			49			
(28) P01033	Metalloproteinase inhibitor 1 precursor					65	
(29) P01034	Cystatin-C			35			
(30) P01040	Cystatin A (stefin A)	772	720	939	229	174	240
(31) P01591	Immunoglobulin J chain						39
(32) P01605	Ig kappa chain V-I region Lay					39	
(33) P01833	Polymeric immunoglobulin receptor	926	895	824		245	282
(34) P01834	Ig kappa chain C region	661	869	685		88	106
(35) P01842	Ig lambda chain C regions	178	323	338		72	95

Accession No		Protein Description	Sample A1	Sample A2	Sample A3	Sample Af	Sample Ar	Sample B
(36)	P01857	Ig gamma-1 chain C region	359	599	578	63	452	188
(37)	P01859	Ig gamma-2 chain C region			169	39	265	165
(38)	P01860	Ig gamma-3 chain C region	228	322				
(39)	P01861	Ig gamma-4 chain C region						108
(40)	P01876	Ig alpha-1 chain C region	287		338		273	
(41)	P01877	Ig alpha-2 chain C region		336				200
(42)	P02042	Hemoglobin subunit delta	2105	2067	1894		1002	925
(43)	P02100	Hemoglobin subunit epsilon		141	127		170	
(44)	P02511	Alpha-crystallin B chain	121	48	89	41	137	100
(45)	P02545	Lamin-A/C	77	166	86		40	
(46)	P02647	Apolipoprotein A1	79				526	82
(47)	P02652	Apolipoprotein A-II precursor					91	
(48)	P02671	Fibrinogen alpha chain precursor	78	96	122		56	
(49)	P02675	Fibrinogen beta chain precursor					34	
(50)	P02749	Apolipoprotein H (beta-2-glycoprotein I)	103	216	202		62	46
(51)	P02763	Alpha-1-acid glycoprotein 1	71	117	70			
(52)	P02765	Alpha-2-HS-glycoprotein		45				
(53)	P02766	Transthyretin		51				
(54)	P02768	Serum albumin precursor	4975	4805	5385	251	1769	2021
(55)	P02774	Vitamin D-binding protein precursor	42	78	52			
(56)	P02787	Serotransferrin	130	428	438		234	138
(57)	P02788	Lactotransferrin	483	871	933	63	502	288
(58)	P02790	Hemopexin	37	46	51		53	38
(59)	P03973	Antileukoproteinase 1 precursor	375	289	380		156	199
(60)	P04075	Fructose-bisphosphate aldolase A	195	320	241	236	132	244
(61)	P04080	Cystatin B	868	1016	795	311	715	293
(62)	P04083	Annexin A1	1735	2406	2059	813	1163	269
(63)	P04114	Apolipoprotein B-100 precursor				31		
(64)	P04279	Semenogelin-1	114	260	316			
(65)	P04406	Glyceraldehyde-3-phosphate dehydrogenase, liver	343	605	558	165	277	215
(66)	P04433	Ig kappa chain V-III region VG precursor					36	
(67)	P04792	Heat-shock protein beta-1	1389	1251	1425	468	1114	701
(68)	P05109	Calgranulin A (S100A8)	588	767	811	378	654	463
(69)	P05154	Plasma serine protease inhibitor precursor						45
(70)	P05164	Myeloperoxidase precursor					96	49
(71)	P05204	Non-histone chromosomal protein HMG-17	73	76	80			
(72)	P05387	60S acidic ribosomal protein P2	64	146	42	30		
(73)	P06702	Calgranulin B (S100A9)	2168	2319	2432	784	982	1229
(74)	P06731	Carcinoembryonic antigen-related cell adhesion molecule 5 precursor	128	112	166			111
(75)	P06733	Alpha-enolase	141	204	224	72	37	
(76)	P06744	Glucose-6-phosphate isomerase					39	
(77)	P06753	Tropomyosin 3	179	174	140			
(78)	P07108	Acyl-CoA binding protein	235	323	357			

Accession No		Protein Description	Sample A1	Sample A2	Sample A3	Sample Af	Sample Ar	Sample B
(79)	P07305	Histone H1.0		49				
(80)	P07355	Annexin A2	345	608	556	183	469	74
(81)	P07476	Involucrin	2251	2091	1571	120	416	717
(82)	P07737	Profilin 1		95			37	
(83)	P07951	Tropomyosin beta chain					53	
(84)	P08107	Heat shock 70 kDa protein 1	100	132	179	100	261	40
(85)	P08123	Collagen alpha 2 T	40	49	62			73
(86)	P08238	Heat shock protein HSP 90-beta					44	
(87)	P08246	Leukocyte elastase precursor		36			36	
(88)	P08311	cathepsin G	321	334	310		215	147
(89)	P08603	Complement factor H	42	56				
(90)	P08670	Vimentin	42					
(91)	P08708	40S ribosomal protein S17					61	
(92)	P09211	Glutathione S-transferase P					74	
(93)	P09466	Glycodelin precursor					32	
(94)	P09497	Clathrin light chain B	45	41				43
(95)	P09651	Heterogeneous nuclear ribonucleoprotein A1					43	
(96)	P0C0S8	Histone H2A type 1	432	589	658			292
(97)	P0C869	Cytosolic phospholipase A2 beta			79			
(98)	P10412	Histone H1.4					42	
(99)	P10599	Thioredoxin	178	157	192		65	93
(100)	P10606	Cytochrome c oxidase subunit 5B, mitochondrial precursor					31	
(101)	P10909	Clusterin precursor	67	101	127	31		
(102)	P11142	Heat shock 70kDa protein 8				90	124	
(103)	P12036	Neurofilament heavy polypeptide				53		
(104)	P12273	Prolactin-inducible protein precursor	87	89			47	
(105)	P12429	Annexin A3					45	
(106)	P12724	Eosinophil cationic protein precursor	215	278	266		138	194
(107)	P12814	Alpha-actinin-1						48
(108)	P13796	Lymphocyte cytosolic protein 1 (L-plastin)					63	
(109)	P13797	Plastin-3					85	
(110)	P13987	CD59 glycoprotein precursor	55		65			
(111)	P14136	Glial fibrillary acidic protein		560	391			
(112)	P14174	Macrophage migration inhibitory factor				53	67	
(113)	P14384	Carboxypeptidase M		54	35			
(114)	P14618	Pyruvate kinase isozymes M1/M2	93	115	138	34	119	
(115)	P15056	B-Raf proto-oncogene serine/threonine-protein kinase				35		
(116)	P15104	Glutamine synthetase						51
(117)	P15259	Phosphoglycerate mutase 2		35				
(118)	P15311	Ezrin	160	182	174		37	56
(119)	P15924	Desmoplakin	312	471	492	90	203	156
(120)	P16035	Metalloproteinase inhibitor 2		57				
(121)	P16401	Histone H1.5 (Histone H1a)	64	72			31	61

Accession No		Protein Description	Sample A1	Sample A2	Sample A3	Sample Af	Sample Ar	Sample B
(122)	P16402	Histone H1.3	102		118			
(123)	P16403	Histone H1.2						99
(124)	P17900	Ganglioside GM2 activator precursor	151	58	141			
(125)	P17931	Galectin-3	89	217	113		57	107
(126)	P18054	Arachidonate 12-lipoxygenase, 12S-type	64		80			55
(127)	P18206	Vinculin	77	135	78			
(128)	P18510	Interleukin 1 receptor antagonist protein	86	122			58	
(129)	P18621	60S ribosomal protein L17 (L23) isoform 5		74	42			
(130)	P18669	Phosphoglycerate mutase 1					80	
(131)	P19447	TFIIH basal transcription factor complex helicase XPB subunit				31		
(132)	P19957	Elafin precursor	473	461	476	65	155	78
(133)	P20160	Azurocidin 1 (cationic antimicrobial protein 37)	98	128	44		135	95
(134)	P20810	Calpastatin (Calpain inhibitor) (Sperm BS-17 component)	138	140	191			43
(135)	P20930	Filaggrin	296	236	204			88
(136)	P22528	Cornifin B	342	433	351		84	150
(137)	P22531	Small proline-rich protein 2E	358	403	490			
(138)	P22532	Small proline-rich protein 2D			448			144
(139)	P22735	Protein-glutamine gamma-glutamyltransferase K	205	192	129			
(140)	P23142	Fibulin-1 precursor	56	47	46		58	
(141)	P23528	cofilin-1		73				
(142)	P23786	Carnitine O-palmitoyltransferase 2, mitochondrial			46			
(143)	P24158	Myeloblastin precursor	61	84	84			72
(144)	P25685	DnaJ homolog subfamily B member 1					41	
(145)	P26373	60S ribosomal protein L13		48	45			67
(146)	P27482	Calmodulin-like protein 3	65	180	153		199	94
(147)	P29373	Cellular retinoic acid-binding protein 2					44	
(148)	P29508	Squamous cell carcinoma antigen 1 (SCCA-1); Serpin B3	226	431	416	179	617	120
(149)	P30041	Peroxiredoxin-6		160	52		82	
(150)	P30043	Flavin reductase	42	103	49			47
(151)	P30086	Phosphatidylethanolamine-binding protein	74	76		37	63	54
(152)	P30740	Monocyte/neutrophil elastase inhibitor		85		62	91	124
(153)	P31151	S100 calcium-binding protein A7 (psoriasin)	328	484	472		88	
(154)	P31946	14-3-3 protein beta/alpha		39				
(155)	P31947	Stratifin; 14-3-3 protein sigma					138	
(156)	P31949	S100 calcium-binding protein A11 (calgizzarine)	130	183	200	39		39
(157)	P31997	Carcinoembryonic antigen-related cell adhesion molecule 8 precursor		54			33	
(158)	P32119	Peroxiredoxin-2	78	246	139		155	60
(159)	P32320	Cytidine deaminase		44	43			
(160)	P32926	Desmoglein-3		80				
(161)	P35268	60S ribosomal protein L22					33	
(162)	P35321	Small-proline rich protein 1A; Cornifin A	446	481	528		93	248
(163)	P35325	Small proline-rich protein 2B		355				
(164)	P35326	Small proline-rich protein 2A	443	352	461			

Accession No		Protein Description	Sample A1	Sample A2	Sample A3	Sample Af	Sample Ar	Sample B
(165)	P35579	Myosin-9	312	317	285	83	40	84
(166)	P39019	40S ribosomal protein S19		54				
(167)	P39023	60S ribosomal protein L3	41					46
(168)	P40121	Macrophage-capping protein		51			44	
(169)	P42766	60S ribosomal protein L35		81	43			
(170)	P46776	60S ribosomal protein L27a	52	50	64		44	
(171)	P46778	60S ribosomal protein L21		92	93			
(172)	P47914	60S ribosomal protein L29		74				58
(173)	P47929	Lectin, galactoside-binding, soluble, 7 (galectin 7)	394	496	392	208	350	181
(174)	P48594	Squamous cell carcinoma antigen 2; Serpin B4		320	264	140	344	
(175)	P49773	Histidine triad nucleotide-binding protein 1		82	53		32	
(176)	P49913	Cathelicidin antimicrobial peptide precursor	50	46	35		40	42
(177)	P50914	60S ribosomal protein L14			43		68	
(178)	P52209	6-phosphogluconate dehydrogenase, decarboxylating					43	
(179)	P54108	Cysteine-rich secretory protein 3		49	58	44	76	67
(180)	P54253	Ataxin-1			44			
(181)	P54652	Heat shock-related 70 kDa protein 2				59		
(182)	P55145	ARMET protein precursor	41	44				
(183)	P59665	Neutrophil defensin 1 precursor	137	139	127	290		158
(184)	P59666	Neutrophil defensin 3 precursor					228	
(185)	P60174	Triosephosphate isomerase		40	60			48
(186)	P60709	Actin, cytoplasmic 1	676	799	760		599	365
(187)	P60866	40S ribosomal protein S20	71	109	102		60	
(188)	P60903	S100 calcium binding protein A10	46	121				
(189)	P60985	Keratinocyte differentiation-associated protein		83				
(190)	P61254	60S ribosomal protein L26		45	89		42	
(191)	P61626	Lysozym C	194	249	205	85	125	139
(192)	P61769	Beta-2-microglobulin			35			
(193)	P62081	40S ribosomal protein S7	65	50	58			
(194)	P62158	Calmodulin		95	43			72
(195)	P62249	40S ribosomal protein S16					51	
(196)	P62263	40S ribosomal protein S14			46			
(197)	P62266	40S ribosomal protein S23					39	
(198)	P62269	40S ribosomal protein S18	38	63	50		41	
(199)	P62280	40S ribosomal protein S11					34	
(200)	P62424	60S ribosomal protein L7a		68	62			
(201)	P62736	Actin, aortic smooth muscle				446		
(202)	P62753	40S ribosomal protein S6					71	
(203)	P62805	Histone H4	304	587	507	101	252	395
(204)	P62829	60S ribosomal protein L23						41
(205)	P62847	40S ribosomal protein S24	52	73	67		44	44
(206)	P62851	40S ribosomal protein S25				40		
(207)	P62854	40S ribosomal protein S26		46			52	

Accession No		Protein Description	Sample A1	Sample A2	Sample A3	Sample Af	Sample Ar	Sample B
(208)	P62861	40S ribosomal protein S30						33
(209)	P62899	60S ribosomal protein L31		43	48		33	
(210)	P62906	60S ribosomal protein L10a		39				
(211)	P62910	60S ribosomal protein L32						33
(212)	P62917	60S ribosomal protein L8		65	49			
(213)	P62937	Peptidyl-prolyl cis-trans isomerase A (Cyclophilin A)		119	126	118		
(214)	P62988	Ubiquitin	169	193	338	62	108	97
(215)	P63104	14-3-3 protein zeta/delta	110	81	104		110	
(216)	P63173	60S ribosomal protein L38	49	46	56			
(217)	P63220	40S ribosomal protein S21					32	
(218)	P63313	Thymosin beta-10		59	53			
(219)	P67936	Tropomyosin alpha 4 chain	162	212	160			36
(220)	P68032	Actin, alpha cardiac muscle 1	580	557	570			
(221)	P68104	eukaryotic translation elongation factor 1 alpha 1					136	
(222)	P68366	Tubulin alpha-1 chain					39	
(223)	P68871	Hemoglobin beta chain	3783	3808	4090	782	2274	1744
(224)	P69891	Hemoglobin subunit gamma-1					181	
(225)	P69905	Hemoglobin alpha subunit	1788	1899	1799	543	1246	1231
(226)	P80188	Neutrophil gelatinase-associated lipocalin	310	376	424		74	218
(227)	P80511	Protein S100-A12				32		
(228)	P81605	Dermcidin precursor	134	333				
(229)	P83731	60S ribosomal protein L24			42			
(230)	P84103	Splicing factor, arginine/serine-rich 3			39			
(231)	P98187	Cytochrome P450 4F8	36		33			
(232)	P99999	Cytochrome c	64	84	78		34	46
(233)	Q00796	Sorbitol dehydrogenase		59				
(234)	Q01469	Fatty acid-binding protein, epidermal	163	149	212	70	394	189
(235)	Q01518	CAP, adenylate cyclase-associated protein 1 (yeast)			54			35
(236)	Q01954	Zinc finger protein basonuclin-1		38				
(237)	Q02383	Semenogelin-2	111	199	182			
(238)	Q02487	Desmocollin-2 precursor	114	141	145		38	54
(239)	Q02878	60S ribosomal protein L6	58	84	84		65	
(240)	Q05639	Elongation factor 1-alpha 2	128					104
(241)	Q06323	Proteasome activator complex subunit 1					46	
(242)	Q06830	Peroxiredoxin 1			87	78	189	79
(243)	Q07065	Cytoskeleton associated protein 4					59	
(244)	Q07654	trefoil factor 3 precursor	70	94	118			
(245)	Q08188	Protein-glutamine gamma-glutamyltransferase E precursor					35	
(246)	Q08380	Galectin-3-binding protein precursor					47	
(247)	Q08EQ4	Thymosin beta-4-like protein 1		89				
(248)	Q09666	Neuroblast differentiation-associated protein AHNAK	1398	2831	2229	101	563	183
(249)	Q12888	Tumor suppressor p53-binding protein 1	36					
(250)	Q13835	Plakophilin 1	65		46			

Accession No	Protein Description	Sample A1	Sample A2	Sample A3	Sample Af	Sample Ar	Sample B
(251) Q14116	Interleukin-18 precursor					51	
(252) Q14134	Tripartite motif-containing protein 29	52	80	68	41		70
(253) Q14210	Lymphocyte antigen 6D precursor						45
(254) Q14508	WAP four-disulfide core domain protein 2 precursor	159	157	160	48		80
(255) Q15056	Eukaryotic translation initiation factor 4H				41		
(256) Q15149	Plectin-1	53	164	147			
(257) Q15651	High mobility group nucleosome-binding domain-containing protein 3		57	63			
(258) Q15843	NEDD8			74			
(259) Q15847	Adipose most abundant gene transcript 2 protein						40
(260) Q16610	Extracellular matrix protein 1 precursor	178	211	260		62	
(261) Q16629	Splicing factor, arginine/serine-rich 7	43					
(262) Q16695	Histone H3.1t	79	116	161			130
(263) Q16825	Tyrosine-protein phosphatase non-receptor type 21				33		
(264) Q3KQU3	MAP7 domain-containing protein 1		46	53			
(265) Q5CZC0	Fibrous sheath-interacting protein 2		36				
(266) Q5T0Z8	Uncharacterized protein C6orf132		50				
(267) Q5TZ20	Olfactory receptor 2G6				40		
(268) Q5TZA2	Rootletin	49	49				
(269) Q5VTE0	Putative elongation factor 1-alpha-like 3		194	196			
(270) Q5VTM1	Protein FAM25	229	249	249	147		171
(271) Q6E0U4	Dermokine precursor	63					
(272) Q6P3W6	Neuroblastoma breakpoint family member 10						30
(273) Q6UWP8	Suprabasin precursor	735	958	846	75	179	140
(274) Q6XPR3	Repetin	148	286	164			
(275) Q6ZVX7	Putative uncharacterized protein LOC342897				38		
(276) Q71DI3	Histone H3.2					166	
(277) Q71UM5	40S ribosomal protein S27-like protein						37
(278) Q7L7L0	Histone H2A type 3					187	
(279) Q7Z406	myosin, heavy chain 14 isoform 1		108				
(280) Q86SG5	Protein S100-A7-like 1						48
(281) Q8IUE6	Histone H2A type 2-B					112	124
(282) Q8IUS5	Abhydrolase domain-containing protein 7					41	
(283) Q8IVV2	Lipoxygenase homology domain-containing protein 1		39				
(284) Q8IZQ1	WD repeat and FYVE domain-containing protein 3					45	
(285) Q8N1A0	Keratin-like protein KRT222	99	135	141			
(286) Q8N257	Histone H2B type 3-B		348	394			
(287) Q8NA31	Coiled-coil domain-containing protein 79				34		
(288) Q8NAC3	Interleukin-17 receptor C precursor						30
(289) Q8NCR0	UDP-GalNAc:beta-1,3-N-acetylgalactosaminyltransferase 2				32		
(290) Q8NGC9	Olfactory receptor 11H4		35				37
(291) Q8NHM4	Putative trypsin-6	39	42	55			
(292) Q8NHS3	Major facilitator superfamily domain-containing protein 8		45		38	35	31
(293) Q8TC20	Cancer-associated gene 1 protein				34		

Accession No		Protein Description	Sample A1	Sample A2	Sample A3	Sample Af	Sample Ar	Sample B
(294)	Q8TD31	Coiled-coil alpha-helical rod protein 1						31
(295)	Q8TER0	Sushi, nidogen and EGF-like domain-containing protein 1	44					
(296)	Q8TER5	Protein SOLO				36		
(297)	Q8WWI1	LIM domain only protein 7		37		37		
(298)	Q8WXH0	Nesprin-2				32		
(299)	Q92597	Protein NDRG1			75			
(300)	Q92765	Secreted frizzled-related protein 3 precursor					32	
(301)	Q92817	Envoplakin	176	461	370		52	34
(302)	Q92876	Kallikrein-6 precursor	198	246	257		39	
(303)	Q93077	Histone H2A type 1-C			563			
(304)	Q93100	Phosphorylase b kinase regulatory subunit beta						31
(305)	Q96FF9	Sororin				31		
(306)	Q96FQ6	Protein S100-A16	62	168	231			
(307)	Q96HC4	PDZ and LIM domain protein 5		57	69			
(308)	Q96HE7	ERO1-like protein alpha precursor					38	
(309)	Q96P63	Serpin B12		51	34		42	
(310)	Q99835	Smoothened homolog precursor				32		34
(311)	Q99877	Histone H2B type 1-N	502				220	
(312)	Q99880	Histone H2B.c				118		
(313)	Q9BQE3	Tubulin alpha-1C chain		87				
(314)	Q9BW04	specifically androgen-regulated protein		48	68			
(315)	Q9BYE4	Small proline-rich protein 2G				44		
(316)	Q9BYT8	Neurolysin, mitochondrial			34			
(317)	Q9C0A6	SET domain-containing protein 5				32		
(318)	Q9GZV4	Eukaryotic translation initiation factor 5A-2		74				
(319)	Q9H1E1	Ribonuclease 7 precursor	65	103	142			44
(320)	Q9H7D7	WD repeat-containing protein 26						30
(321)	Q9HAY6	Beta,beta-carotene 15,15'-monooxygenase						31
(322)	Q9HC84	Mucin-5B precursor	75	66	80	56	56	145
(323)	Q9NQ38	Serine protease inhibitor Kazal-type 5	218	282	320	155	50	71
(324)	Q9NYK1	Toll-like receptor 7 precursor				41		
(325)	Q9NZT1	Calmodulin-like protein 5	73	209	67	89	58	49
(326)	Q9P0G3	Kallikrein-14		96	63			
(327)	Q9UBC9	Small proline-rich protein 3	2305	2760	2473	174	1071	1096
(328)	Q9UBG3	Cornulin	1208	1651	1401	195	373	432
(329)	Q9UBX7	Kallikrein 11 precursor	71	112	82		69	79
(330)	Q9UIV8	Serpin B13	69	70	90	39	105	43
(331)	Q9UJY1	Heat shock protein beta-8				43		
(332)	Q9UKR3	Kallikrein 13 precursor	61	69	105		80	
(333)	Q9UL52	Transmembrane protease, serine 11E					42	52
(334)	Q9ULV0	Myosin-5B			36			
(335)	Q9UPQ7	PDZ domain-containing RING finger protein 3				34		
(336)	Q9Y2V2	Calcium-regulated heat stable protein 1		85				

Accession No		Protein Description	Sample A1	Sample A2	Sample A3	Sample Af	Sample Ar	Sample B
(337)	Q9Y3T6	R3H and coiled-coil domain-containing protein 1				33		
(338)	Q9Y446	Plakophilin-3					46	
(339)	Q9Y4K1	Absent in melanoma 1 protein		64	68			
