# Supplementary material for: Comprehensive proteomic analysis of human cervical-vaginal fluid using colposcopy samples
Source: Proteome Sci. 2009 Apr 17;7:17. doi: 10.1186/1477-5956-7-17 (PMC2678104; doi:10.1186/1477-5956-7-17)
Supplement: Additional file 3 — Overview of the proteins which were uniquely identified in our study. [file 1477-5956-7-17-S3.pdf]

Additional file 3 – Overview of the proteins which were uniquely identified in our study.

Classification according to their functional process and cellular localization is also presented.

Accession no		Protein description	Cellular localization	Functional proces
(1)	A6NL28	Putative tropomyosin alpha-3 chain-like protein	Cytoplasm	Cell structure and mobility
(2)	A8MQ03	UPF0574 protein C9orf169	ND	ND
(3)	A9Z1Y9	Thymosin beta-4-like protein 6	Cytoplasm	Cell structure and mobility
(4)	O00555	Voltage-dependent P/Q-type calcium channel subunit alpha-1A	Nucleus	Muscle contraction
				Neuronal activities
				Transport
(5)	O15144	Actin-related protein 2/3 complex subunit 2	Cytoskeleton	Protein metabolism and modification
				Cell structure and mobility
(6)	O15263	Beta-defensin 2 precursor	Extracellular Region	Immunity and defense
				Nucleoside, nucleotide and nucleic acid metabolism
(7)	O60814	Histone H2B type 1-K	Nucleus	Immunity and defense
				Nucleoside, nucleotide and nucleic acid metabolism
(8)	O94823	Probable phospholipid-transporting ATPase VB	Membrane	Lipid, fatty acid and steroid metabolism
				Transport
(9)	P00915	Carbonic anhydrase 1	Cytoplasm	Nucleoside, nucleotide and nucleic acid metabolism
(10)	P00918	Carbonic anhydrase 2	Cytoplasm	Nucleoside, nucleotide and nucleic acid metabolism
(11)	P01033	Metalloproteinase inhibitor 1 precursor	Extracellular Region	Developmental processes
				Protein metabolism and modification
(12)	P01034	Cystatin-C	Extracellular Region	Protein metabolism and modification
(13)	P01605	Ig kappa chain V-I region Lay	Extracellular Region	Immunity and defense
(14)	P02042	Hemoglobin subunit delta	Cytoplasm	Blood circulation and gas exchange
				Transport
(15)	P02100	Hemoglobin subunit epsilon	Cytoplasm	Blood circulation and gas exchange
				Transport
(16)	P02652	Apolipoprotein A-II precursor	Extracellular Region	Lipid, fatty acid and steroid metabolism
				Transport
(17)	P04114	Apolipoprotein B-100 precursor	Extracellular Region	Lipid, fatty acid and steroid metabolism
				Transport
(18)	P04433	Ig kappa chain V-III region VG precursor	Extracellular Region	Immunity and defense
(19)	P06744	Glucose-6-phosphate isomerase	Extracellular Region	Carbohydrate metabolism

Accession no		Protein description	Cellular localization	Functional proces
(20)	P07305	Histone H1.0	Cytoskeleton	Nucleoside, nucleotide and nucleic acid metabolism
(21)	P07951	Tropomyosin beta chain	Cytoskeleton	Cell structure and mobility
				Developmental processes
				Muscle contraction
(22)	P08708	40S ribosomal protein S17	Cytoplasm	Protein metabolism and modification
(23)	P09466	Glycodelin precursor	Extracellular Region	Developmental processes
(24)	P09497	Clathrin light chain B	Golgi Apparatus	Intracellular protein traffic
(25)	P0C0S8	Histone H2A type 1	Nucleus	Nucleoside, nucleotide and nucleic acid metabolism
(26)	P0C869	Cytosolic phospholipase A2 beta	Cytoplasm	Immunity and defense
				Lipid, fatty acid and steroid metabolism
				Cell proliferation and differentiation
(27)	P10412	Histone H1.4	Intracellular	Nucleoside, nucleotide and nucleic acid metabolism
(28)	P10606	Cytochrome c oxidase subunit 5B, mitochondrial precursor	Mitochondrion	Electron transport
(29)	P12036	Neurofilament heavy polypeptide	Cytoskeleton	Cell structure and mobility
(30)	P14136	Glial fibrillary acidic protein	Cytoskeleton	Cell structure and mobility
(31)	P14174	Macrophage migration inhibitory factor	Extracellular Region	Immunity and defense
(32)	P14384	Carboxypeptidase M	Membrane	Protein metabolism and modification
(33)	P15056	B-Raf proto-oncogene serine/threonine-protein kinase	Membrane	Apoptosis
				Cell proliferation and differentiation
				Oncogenesis
				Signal transduction
(34)	P15104	Glutamine synthetase	Mitochondrion	Amino acid metabolism
				Other metabolism
(35)	P16035	Metalloproteinase inhibitor 2	Extracellular Region	Protein metabolism and modification
(36)	P19447	TFIIH basal transcription factor complex helicase XPB subunit	Nucleus	Nucleoside, nucleotide and nucleic acid metabolism
(37)	P22531	Small proline-rich protein 2E	Cytoskeleton	Developmental processes
				Cell proliferation and differentiation
(38)	P23786	Carnitine O-palmitoyltransferase 2, mitochondrial	Envelope	Amino acid metabolism
				Lipid, fatty acid and steroid metabolism
(39)	P25685	DnaJ homolog subfamily B member 1	Nucleus	Protein metabolism and modification
(40)	P26373	60S ribosomal protein L13	Cytoplasm	Protein metabolism and modification
(41)	P30041	Peroxiredoxin-6	Cytoplasm	Immunity and defense
(42)	P31946	14-3-3 protein beta/alpha	Cytoplasm	Cell cycle

Accession no		***Protein description***	***Cellular localization***	***Functional proces***
				Protein targeting and localization
				Signal transduction
(43)	P31997	Carcinoembryonic antigen-related cell adhesion molecule 8 precursor	Extracellular Region	Cell adhesion
				Signal transduction
(44)	P35268	60S ribosomal protein L22	Cytoplasm	Protein metabolism and modification
(45)	P39019	40S ribosomal protein S19	Nucleus	Protein metabolism and modification
(46)	P39023	60S ribosomal protein L3	Nucleus	Protein metabolism and modification
(47)	P40121	Macrophage-capping protein	Cytoskeleton	Cell structure and mobility
(48)	P42766	60S ribosomal protein L35	Nucleus	Protein metabolism and modification
(49)	P46776	60S ribosomal protein L27a	Cytoplasm	Protein metabolism and modification
(50)	P46778	60S ribosomal protein L21	Cytoplasm	Protein metabolism and modification
(51)	P47914	60S ribosomal protein L29	Cytoplasm	Protein metabolism and modification
(52)	P49773	Histidine triad nucleotide-binding protein 1	Cytoskeleton	Signal transduction
(53)	P49913	Cathelicidin antimicrobial peptide precursor	Extracellular Region	Immunity and defense
(54)	P50914	60S ribosomal protein L14	Cytoplasm	Protein metabolism and modification
(55)	P54253	Ataxin-1	Nucleus	Nucleoside, nucleotide and nucleic acid metabolism
(56)	P54652	Heat shock-related 70 kDa protein 2	Cell Surface	Immunity and defense
				Protein metabolism and modification
(57)	P59666	Neutrophil defensin 3 precursor	Extracellular Region	Immunity and defense
(58)	P60866	40S ribosomal protein S20	Cytoplasm	Protein metabolism and modification
(59)	P60985	Keratinocyte differentiation-associated protein	Extracellular Region	Cell proliferation and differentiation
				Developmental processes
(60)	P61254	60S ribosomal protein L26	Cytoplasm	Protein metabolism and modification
(61)	P61769	Beta-2-microglobulin	Cytoplasm	Immunity and defense
(62)	P62081	40S ribosomal protein S7	Nucleus	Protein metabolism and modification
(63)	P62249	40S ribosomal protein S16	Cytoplasm	Protein metabolism and modification
(64)	P62263	40S ribosomal protein S14	Cytoplasm	Protein metabolism and modification
(65)	P62266	40S ribosomal protein S23	Cytoplasm	Protein metabolism and modification
(66)	P62269	40S ribosomal protein S18	Cytoplasm	Protein metabolism and modification
(67)	P62280	40S ribosomal protein S11	Cytoplasm	Protein metabolism and modification
(68)	P62424	60S ribosomal protein L7a	Membrane Fraction	Protein metabolism and modification
(69)	P62736	Actin, aortic smooth muscle	Cytoskeleton	Cell cycle
				Cell structure and mobility

Accession no	***Protein description***	***Cellular localization***	***Functional proces***
			Intracellular protein traffic<br>Transport
(70) P62829	60S ribosomal protein L23	Cytoplasm	Protein metabolism and modification
(71) P62847	40S ribosomal protein S24	Cytoplasm	Protein metabolism and modification
(72) P62851	40S ribosomal protein S25	Cytoplasm	Protein metabolism and modification
(73) P62854	40S ribosomal protein S26	Cytoplasm	Protein metabolism and modification
(74) P62861	40S ribosomal protein S30	Cytoplasm	Protein metabolism and modification
(75) P62899	60S ribosomal protein L31	Cytoplasm	Protein metabolism and modification
(76) P62906	60S ribosomal protein L10a	Cytoplasm	Protein metabolism and modification
(77) P62910	60S ribosomal protein L32	Cytoplasm	Protein metabolism and modification
(78) P62917	60S ribosomal protein L8	Cytoplasm	Protein metabolism and modification
(79) P63173	60S ribosomal protein L38	Cytoplasm	Protein metabolism and modification
(80) P63220	40S ribosomal protein S21	Cytoplasm	Protein metabolism and modification
(81) P68032	Actin, alpha cardiac muscle 1	Cytoskeleton	Cell cycle<br>Cell structure and mobility<br>Intracellular protein traffic<br>Transport
(82) P69891	Hemoglobin subunit gamma-1	Cytoplasm	Blood circulation and gas exchange<br>Transport
(83) P83731	60S ribosomal protein L24	Cytoplasm	Protein metabolism and modification
(84) P84103	Splicing factor, arginine/serine-rich 3	Nucleus	Nucleoside, nucleotide and nucleic acid metabolism
(85) P98187	Cytochrome P450 4F8	Endoplasmic Reticulum	Electron transport<br>Lipid, fatty acid and steroid metabolism
(86) Q00796	Sorbitol dehydrogenase	ND	Carbohydrate metabolism<br>Other metabolism
(87) Q01954	Zinc finger protein basonuclin-1	Nucleus	Developmental processes<br>Sensory perception
(88) Q02878	60S ribosomal protein L6	Cytoplasm	Protein metabolism and modification
(89) Q05639	Elongation factor 1-alpha 2	Nucleus	Protein metabolism and modification
(90) Q06323	Proteasome activator complex subunit 1	Cytoplasm	Protein metabolism and modification
(91) Q08EQ4	Thymosin beta-4-like protein 1	Cytoplasm	Cell structure and mobility
(92) Q12888	Tumor suppressor p53-binding protein 1	Nucleus	Nucleoside, nucleotide and nucleic acid metabolism
(93) Q14116	Interleukin-18 precursor	Extracellular Region	Immunity and defense

Accession no		Protein description	Cellular localization	Functional proces
				Signal transduction
(94)	Q14210	Lymphocyte antigen 6D precursor	Membrane	Cell adhesion
(95)	Q15056	Eukaryotic translation initiation factor 4H	Cytoplasm	Protein metabolism and modification
(96)	Q15651	High mobility group nucleosome-binding domain-containing protein 3	Nucleus	Cell cycle
				Nucleoside, nucleotide and nucleic acid metabolism
(97)	Q15843	NEDD8	Nucleus	Protein metabolism and modification
(98)	Q15847	Adipose most abundant gene transcript 2 protein	Cellular	ND
(99)	Q16629	Splicing factor, arginine/serine-rich 7	Nucleus	Nucleoside, nucleotide and nucleic acid metabolism
(100)	Q16695	Histone H3.1t	Intracellular	Nucleoside, nucleotide and nucleic acid metabolism
(101)	Q16825	Tyrosine-protein phosphatase non-receptor type 21	Cytoskeleton	Cell structure and mobility
				Developmental processes
				Protein metabolism and modification
(102)	Q3KQU3	MAP7 domain-containing protein 1	ND	Cell structure and mobility
				Developmental processes
(103)	Q5CZC0	Fibrous sheath-interacting protein 2	ND	ND
(104)	Q5T0Z8	Uncharacterized protein C6orf132	ND	ND
(105)	Q5TZ20	Olfactory receptor 2G6	Membrane	Signal transduction
				Neuronal activities
				Sensory perception
(106)	Q5TZA2	Rootletin	Cytoskeleton	Cell cycle
(107)	Q5VTE0	Putative elongation factor 1-alpha-like 3	Cytoplasm	Protein metabolism and modification
(108)	Q5VTM1	Protein FAM25	ND	ND
(109)	Q6P3W6	Neuroblastoma breakpoint family member 10	Cytoplasm	ND
(110)	Q6XPR3	Repetin	Extracellular Region	Cell proliferation and differentiation
				Developmental processes
(111)	Q71DI3	Histone H3.2	Nucleus	Nucleoside, nucleotide and nucleic acid metabolism
(112)	Q71UM5	40S ribosomal protein S27-like protein	Cellular	Protein metabolism and modification
(113)	Q7L7L0	Histone H2A type 3	Nucleus	Nucleoside, nucleotide and nucleic acid metabolism
(114)	Q8IUE6	Histone H2A type 2-B	Nucleus	Nucleoside, nucleotide and nucleic acid metabolism
(115)	Q8IUS5	Abhydrolase domain-containing protein 7	Membrane	Immunity and defense
				Nucleoside, nucleotide and nucleic acid metabolism
(116)	Q8IVV2	Lipoxygenase homology domain-containing protein 1	ND	Protein metabolism and modification
(117)	Q8IZQ1	WD repeat and FYVE domain-containing protein 3	Cytoplasm	Nucleoside, nucleotide and nucleic acid metabolism

Accession no		Protein description	Cellular localization	Functional proces
				Protein targeting and localization
				Signal transduction
(118)	Q8N1A0	Keratin-like protein KRT222	Cytoskeleton	Cell structure and mobility
(119)	Q8N257	Histone H2B type 3-B	Nucleus	Nucleoside, nucleotide and nucleic acid metabolism
(120)	Q8NA31	Coiled-coil domain-containing protein 79	ND	Nucleoside, nucleotide and nucleic acid metabolism
(121)	Q8NAC3	Interleukin-17 receptor C precursor	Membrane	ND
(122)	Q8NCR0	UDP-GalNAc:beta-1,3-N-acetylgalactosaminyltransferase 2	Golgi Apparatus	Protein metabolism and modification
(123)	Q8NGC9	Olfactory receptor 11H4	Membrane	Sensory perception
				Signal transduction
(124)	Q8NHM4	Putative trypsin-6	Extracellular Region	Cell structure and mobility
				Protein metabolism and modification
(125)	Q8NHS3	Major facilitator superfamily domain-containing protein 8	Lysosome	Immunity and defense
(126)	Q8TC20	Cancer-associated gene 1 protein	ND	ND
(127)	Q8TD31	Coiled-coil alpha-helical rod protein 1	Nucleus	Intracellular protein traffic
				Protein targeting and localization
				Developmental processes
(128)	Q8TER0	Sushi, nidogen and EGF-like domain-containing protein 1	ND	Cell structure and mobility
				Developmental processes
				Immunity and defense
				Signal transduction
(129)	Q8TER5	Protein SOLO	Intracellular	Signal transduction
(130)	Q8WXH0	Nesprin-2	Nucleus	Cell structure and mobility
(131)	Q92765	Secreted frizzled-related protein 3 precursor	Extracellular Region	Signal transduction
(132)	Q93077	Histone H2A type 1-C	Intracellular	Nucleoside, nucleotide and nucleic acid metabolism
(133)	Q93100	Phosphorylase b kinase regulatory subunit beta	Membrane	Carbohydrate metabolism
(134)	Q96FF9	Sororin	Nucleus	Cell cycle
(135)	Q96FQ6	Protein S100-A16	ND	ND
(136)	Q96HC4	PDZ and LIM domain protein 5	Cytoskeleton	Cell structure and mobility
				Developmental processes
(137)	Q99835	Smoothened homolog precursor	Membrane	Signal transduction
(138)	Q99877	Histone H2B type 1-N	Intracellular	Other metabolism
(139)	Q9BQE3	Tubulin alpha-1C chain	Cytoskeleton	Cell cycle
				Cell structure and mobility

Accession no		Protein description	Cellular localization	Functional proces
				Intracellular protein traffic
(140)	Q9BYE4	Small proline-rich protein 2G	Cytoskeleton	Developmental processes
				Cell proliferation and differentiation
(141)	Q9BYT8	Neurolysin, mitochondrial	Mitochondrion	Intracellular protein traffic
				Protein metabolism and modification
(142)	Q9C0A6	SET domain-containing protein 5	Nucleus	Oncogenesis
(143)	Q9GZV4	Eukaryotic translation initiation factor 5A-2	Cytoplasm	Protein metabolism and modification
(144)	Q9H7D7	WD repeat-containing protein 26	Cytoplasm	Signal transduction
(145)	Q9HAY6	Beta,beta-carotene 15,15'-monooxygenase	ND	Coenzyme and prosthetic group metabolism
				Sensory perception
(146)	Q9NYK1	Toll-like receptor 7 precursor	Membrane	Developmental processes
				Signal transduction
(147)	Q9P0G3	Kallikrein-14	Extracellular Region	Protein metabolism and modification
(148)	Q9UPQ7	PDZ domain-containing RING finger protein 3	ND	Signal transduction
(149)	Q9Y2V2	Calcium-regulated heat stable protein 1	Cytoplasm	Nucleoside, nucleotide and nucleic acid metabolism
				Signal transduction
(150)	Q9Y3T6	R3H and coiled-coil domain-containing protein 1	ND	ND
(151)	Q9Y446	Plakophilin-3	Cytoskeleton	Cell adhesion
				Signal transduction
