# Supplementary material for: Comprehensive proteomic analysis of human cervical-vaginal fluid using colposcopy samples
Source: Proteome Sci. 2009 Apr 17;7:17. doi: 10.1186/1477-5956-7-17 (PMC2678104; doi:10.1186/1477-5956-7-17)

Additional file 5 – Classification according to cellular localisation of the identified proteins in this study

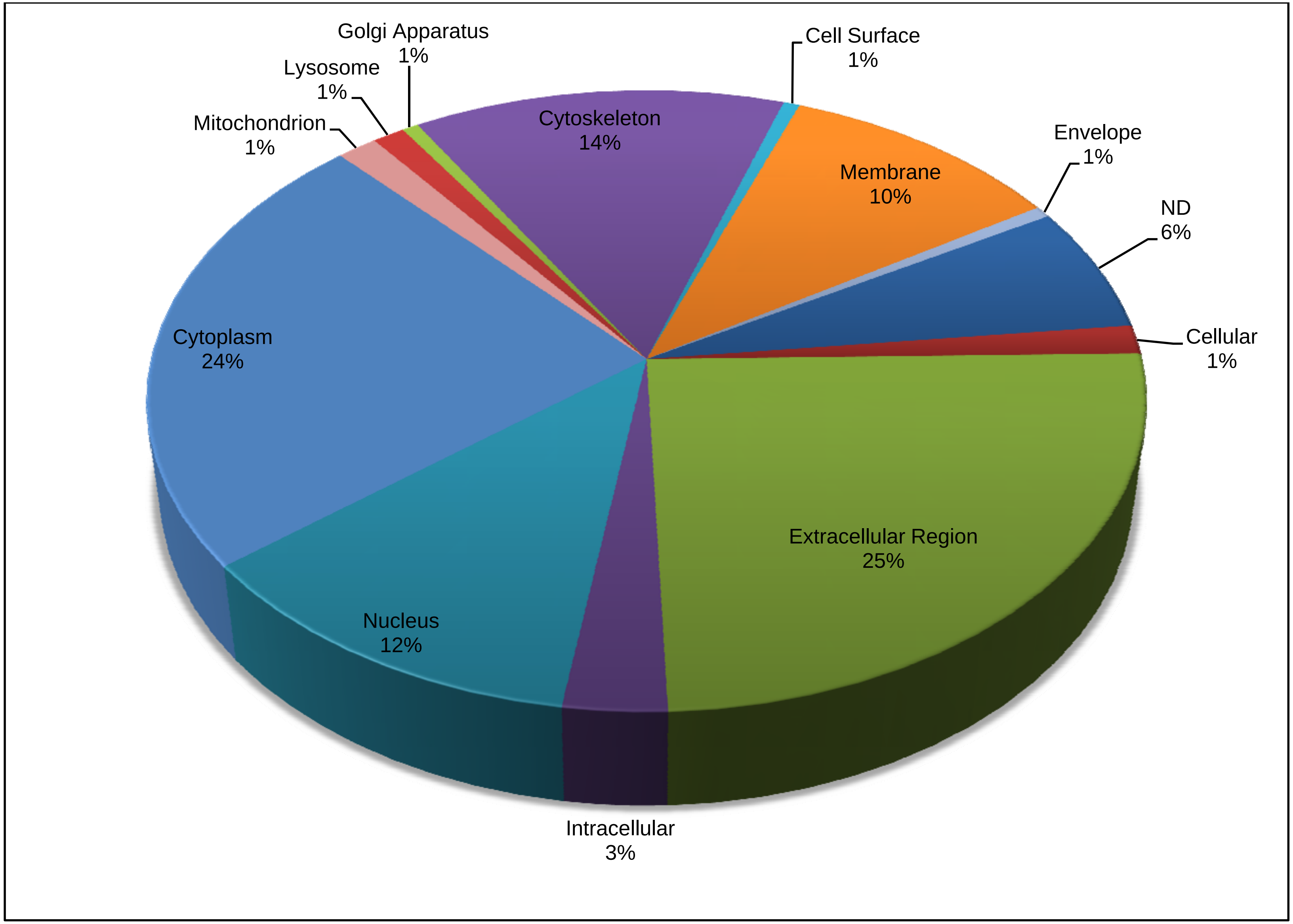

Supplement: Additional file 5 — Classification according to cellular localisation of the identified proteins in this study. [file 1477-5956-7-17-S5.pdf]
