# Supplementary material for: Comprehensive proteomic analysis of human cervical-vaginal fluid using colposcopy samples
Source: Proteome Sci. 2009 Apr 17;7:17. doi: 10.1186/1477-5956-7-17 (PMC2678104; doi:10.1186/1477-5956-7-17)
Supplement: Additional file 6 — Overview of the identifications with their corresponding spectral counting information obtained in the different experiments performed in our study. [file 1477-5956-7-17-S6.pdf]

Additional file 6 – Overview of the identifications with their corresponding spectral counting information obtained in the different experiments performed in our study

Sample A1-3: six pooled samples, C_4 fractionated on protein level, C_{18} separation on peptide level, replicated 3 times. Sample Af and Ar: same samples pooled as sample A, filtrated (sample Af: filtrate; sample Ar: retentate), C_4 fractionated on protein level, C_{18} separation on peptide level. Sample B: one single sample, unpooled, C_4 fractionated on protein level, C_{18} separation on peptide level. Sample A1-3, Af, Ar and B: a score >32 corresponds with p<0,05. Proteins highlighted in yellow are part of the overlapping protein set determined by comparing the four most comprehensive proteomics studies on human CVF.

NSCF: normalized spectral counts factor: $(NSCF)_k = \left(\dfrac{SC_k}{\sum_{i=1}^{N} SC_i} \right) \times 1000$

NSAF: normalized spectral abundance factor: $(NSAF)_k = \left(\dfrac{(SC/OP)_k}{\sum_{i=1}^{N} (SC/OP)_i} \right) \times 1000$

Where SC is the absolute number of MS/MS spectral counts for protein *k*, *OP* is the number of observable peptides for protein *k* and *N* is the total number of proteins identified in one experiment.

	Accession no	Protein Description	Sample A1		Sample A2		Sample A3		Sample Af		Sample Ar		Sample Af+r		Sample B	
			NSCF	NSAF	NSCF	NSAF	NSCF	NSAF	NSCF	NSAF	NSCF	NSAF	NSCF	NSAF	NSCF	NSAF
(1)	P06702	Calgranulin B (S100A9)	50,577	60,479	51,736	61,466	48,692	57,065	82,447	101,045	37,640	48,857	50,037	63,904	66,757	75,706
(2)	P69905	Hemoglobin alpha subunit	36,380	58,003	31,995	50,683	32,703	51,103	50,532	82,575	32,553	56,340	37,528	63,904	43,597	65,921
(3)	Q9UBC9	Small proline-rich protein 3	51,464	54,702	48,332	51,042	43,605	45,425	15,957	17,384	42,726	49,297	35,320	40,096	55,858	56,308
(4)	P68871	Hemoglobin beta chain	50,577	43,984	37,440	32,351	42,151	35,927	63,830	56,894	44,761	42,255	50,037	46,475	50,409	41,575
(5)	P35326	Small proline-rich protein 2A	7,986	38,197	3,404	16,175	5,087	23,848								
(6)	P05109	Calgranulin A (S100A8)	22,183	35,368	19,061	30,194	26,890	42,018	45,213	73,883	29,502	51,058	33,848	57,638	34,060	51,501
(7)	P0C0S8	Histone H2A type 1	10,648	33,953	17,018	53,918	10,174	31,797							12,262	37,081
(8)	P02768	Serum albumin precursor	115,350	33,438	82,369	23,724	89,390	25,397	31,915	9,482	65,107	20,487	55,923	17,314	98,093	26,968
(9)	P04080	Cystatin B	12,422	29,709	12,934	30,733	8,721	20,441	15,957	39,114	21,363	55,459	19,868	50,747	16,349	37,081
(10)	Q9UBG3	Cornulin	33,718	29,323	21,784	18,822	22,529	19,202	34,574	30,817	23,398	22,088	26,490	24,605	19,074	15,731
(11)	Q99877	Histone H2B type 1-N	14,197	27,162							9,156	19,015	6,623	13,533		
(12)	P35321	Small-proline rich protein 1A; Cornifin A	10,648	25,465	7,488	17,793	6,541	15,331			5,086	13,205	3,679	9,398	9,537	21,630
(13)	P04792	Heat-shock protein beta-1	23,070	22,069	18,380	17,469	23,983	22,485	47,872	46,937	39,674	41,198	41,943	42,853	34,060	30,901
(14)	P01834	Ig kappa chain C region	11,535	22,069	9,530	18,116	9,448	17,716			2,035	4,225	1,472	3,007	4,087	7,416
(15)	P01040	Cystatin A (stefin A)	12,422	19,806	10,211	16,175	13,081	20,441	23,936	39,114	4,069	7,042	9,566	16,289	12,262	18,540
(16)	P22528	Cornifin B	6,211	19,806	5,446	17,254	6,541	20,441			1,017	3,521	0,736	2,506	1,362	4,120
(17)	P02042	Hemoglobin subunit delta	21,295	18,520	14,976	12,940	16,715	14,247			11,190	10,564	8,094	7,518	23,161	19,102
(18)	P03973	Antileukoproteinase 1 precursor	8,873	16,976	3,404	6,470	6,541	12,265			5,086	10,564	3,679	7,518	8,174	14,832
(19)	P19957	Elafin precursor	7,098	16,976	6,127	14,558	10,174	23,848	2,660	6,519	3,052	7,923	2,943	7,518	2,725	6,180
(20)	P47929	Lectin, galactoside-binding, soluble, 7 (galectin 7)	12,422	13,204	10,211	10,784	10,901	11,356	26,596	28,974	15,259	17,606	18,396	20,884	10,899	10,987
(21)	P62805	Histone H4	7,986	12,732	8,850	14,019	9,448	14,763	7,979	13,038	8,138	14,085	8,094	13,783	13,624	20,600

Accession no		Protein Description	Sample A1		Sample A2		Sample A3		Sample Af		Sample Ar		Sample Af+r		Sample B	
			NSCF	NSAF	NSCF	NSAF	NSCF	NSAF	NSCF	NSAF	NSCF	NSAF	NSCF	NSAF	NSCF	NSAF
(22)	Q5VTM1	Protein FAM25	5,324	12,732	4,765	11,323	5,087	11,924	18,617	45,633			5,151	13,157	6,812	15,450
(23)	P07108	Acyl-CoA binding protein	5,324	12,732	6,807	16,175	7,994	18,738								
(24)	P07476	Involucrin	30,169	12,025	25,187	9,975	21,076	8,233	7,979	3,260	8,138	3,521	8,094	3,446	24,523	9,270
(25)	P10599	Thioredoxin	7,098	11,318	4,084	6,470	4,360	6,814			2,035	3,521	1,472	2,506	5,450	8,240
(26)	Q6UWP8	Suprabasin precursor	11,535	11,035	12,934	12,293	10,174	9,539	10,638	10,430	4,069	4,225	5,887	6,014	4,087	3,708
(27)	P04083	Annexin A1	23,957	10,913	26,549	12,016	24,709	11,032	58,511	27,318	26,450	13,079	35,320	17,184	10,899	4,709
(28)	Q16695	Histone H3.1t	4,437	10,610	5,446	12,940	5,087	11,924							9,537	21,630
(29)	P31151	S100 calcium-binding protein A7 (psoriasin)	5,324	10,186	9,530	18,116	9,448	17,716			2,035	4,225	1,472	3,007		
(30)	P01842	Ig lambda chain C regions	2,662	8,488	4,765	15,097	4,360	13,627			2,035	7,042	1,472	5,012	4,087	12,360
(31)	P31949	S100 calcium-binding protein A11 (calgizzarine)	3,549	8,488	2,042	4,853	2,180	5,110	2,660	6,519			0,736	1,880	1,362	3,090
(32)	P49913	Cathelicidin antimicrobial peptide precursor	0,887	8,488	0,681	6,470	0,727	6,814			1,017	10,564	0,736	7,518	1,362	12,360
(33)	A9Z1Y9	Thymosin beta-4-like protein 6	0,887	8,488			0,727	6,814								
(34)	P05204	Non-histone chromosomal protein HMG-17	0,887	8,488	0,681	6,470	0,727	6,814								
(35)	P22531	Small proline-rich protein 2E	2,662	8,488	3,404	10,784	4,360	13,627								
(36)	P60709	Actin, cytoplasmic 1	13,310	7,490	12,934	7,231	13,808	7,615			26,450	16,156	19,132	11,498	17,711	9,452
(37)	Q01469	Fatty acid-binding protein, epidermal	6,211	7,427	2,042	2,426	3,634	4,259	7,979	9,779	16,277	21,127	13,981	17,855	9,537	10,815
(38)	P61626	Lysozym C	5,324	7,276	4,765	6,470	3,634	4,867	10,638	14,901	8,138	12,073	8,830	12,888	6,812	8,829
(39)	P62988	Ubiquitin	3,549	6,791	3,404	6,470	5,087	9,539	2,660	5,215	2,035	4,225	2,208	4,511	4,087	7,416
(40)	P68032	Actin, alpha cardiac muscle 1	11,535	6,491	6,807	3,806	6,541	3,607								
(41)	P81605	Dermcidin precursor	2,662	6,366	2,723	6,470										
(42)	P01857	Ig gamma-1 chain C region	7,098	6,173	11,572	9,999	13,081	11,150	5,319	4,741	21,363	20,167	16,924	15,720	10,899	8,989
(43)	P12724	Eosinophil cationic protein precursor	3,549	5,659	2,723	4,313	2,907	4,542			3,052	5,282	2,208	3,759	5,450	8,240
(44)	P04406	Glyceraldehyde-3-phosphate dehydrogenase, liver	7,098	5,224	6,807	4,977	9,448	6,814	21,277	16,047	12,208	9,751	14,717	11,566	10,899	7,606
(45)	P59665	Neutrophil defensin 1 precursor	2,662	5,093	2,723	5,176	2,180	4,088	34,574	67,798			9,566	19,547	8,174	14,832
(46)	P08311	cathepsin G	5,324	5,093	5,446	5,176	5,814	5,451			7,121	7,395	5,151	5,263	6,812	6,180
(47)	O43240	Kallikrein-10 precursor	5,324	4,630	4,765	4,117	7,267	6,194	5,319	4,741	6,104	5,762	5,887	5,468	1,362	1,124
(48)	P01876	Ig alpha-1 chain C region	5,324	4,630			4,360	3,717			8,138	7,683	5,887	5,468		
(49)	P07355	Annexin A2	9,760	4,244	14,295	6,176	11,628	4,955	21,277	9,482	21,363	10,084	21,339	9,910	2,725	1,124
(50)	P04075	Fructose-bisphosphate aldolase A	7,098	4,244	7,488	4,448	7,267	4,259	18,617	11,408	6,104	3,961	9,566	6,108	10,899	6,180
(51)	P80188	Neutrophil gelatinase-associated lipocalin	5,324	4,244	4,765	3,774	5,814	4,542			4,069	3,521	2,943	2,506	8,174	6,180
(52)	Q14508	WAP four-disulfide core domain protein 2 precursor	1,775	4,244	1,361	3,235	2,907	6,814	2,660	6,519			0,736	1,880	2,725	6,180
(53)	P24158	Myeloblastin precursor	1,775	4,244	1,361	3,235	1,453	3,407							2,725	6,180
(54)	Q07654	trefoil factor 3 precursor	1,775	4,244	1,361	3,235	3,634	8,517								
(55)	O60437	Periplakin	39,929	3,858	42,886	4,117	38,517	3,648	7,979	0,790	48,830	5,122	37,528	3,873	27,248	2,497
(56)	P20160	Azurocidin 1 (cationic antimicrobial protein 37)	1,775	3,395	2,042	3,882	1,453	2,725			6,104	12,676	4,415	9,022	5,450	9,888
(57)	Q9NZT1	Calmodulin-like protein 5	2,662	3,183	4,765	5,661	2,180	2,555	7,979	9,779	1,017	1,320	2,943	3,759	1,362	1,545
(58)	P32119	Peroxiredoxin-2	2,662	3,183	2,723	3,235	2,180	2,555			3,052	3,961	2,208	2,819	2,725	3,090
(59)	P01833	Polymeric immunoglobulin receptor	11,535	3,153	8,850	2,403	9,448	2,531			10,173	3,018	7,358	2,148	17,711	4,591
(60)	Q05639	Elongation factor 1-alpha 2	4,437	3,032											5,450	3,531

Accession no		Protein Description	Sample A1		Sample A2		Sample A3		Sample Af		Sample Ar		Sample Af+r		Sample B	
			NSCF	NSAF	NSCF	NSAF	NSCF	NSAF	NSCF	NSAF	NSCF	NSAF	NSCF	NSAF	NSCF	NSAF
(61)	P01860	Ig gamma-3 chain C region	4,437	3,032	4,084	2,773										
(62)	P02511	Alpha-crystallin B chain	2,662	2,829	1,361	1,438	2,180	2,271	2,660	2,897	4,069	4,695	3,679	4,177	4,087	4,120
(63)	O95274	Ly6/PLAUR domain-containing protein 3 precursor	2,662	2,829	2,723	2,876	2,907	3,028			4,069	4,695	2,943	3,341	4,087	4,120
(64)	O15263	Beta-defensin 2 precursor	0,887	2,829	0,681	2,157	0,727	2,271							1,362	4,120
(65)	P60903	S100 calcium binding protein A10; Calpactin I light chain	0,887	2,829	2,042	6,470										
(66)	P06753	Tropomyosin 3	4,437	2,829	2,042	1,294	1,453	0,908								
(67)	P13987	CD59 glycoprotein precursor	1,775	2,829			0,727	1,136								
(68)	P63173	60S ribosomal protein L38	0,887	2,829	0,681	2,157	0,727	2,271								
(69)	P16402	Histone H1.3	1,775	2,829			2,180	3,407								
(70)	P29508	Squamous cell carcinoma antigen 1 (SCCA-1); Serpin B3	6,211	2,701	6,127	2,647	6,541	2,787	18,617	8,297	27,467	12,965	25,018	11,619	6,812	2,809
(71)	P02788	Lactotransferrin	9,760	2,594	14,976	3,954	14,535	3,785	2,660	0,724	19,329	5,575	14,717	4,177	16,349	4,120
(72)	P60866	40S ribosomal protein S20	0,887	2,122	2,042	4,853	1,453	3,407			1,017	2,641	0,736	1,880		
(73)	O15231	Zinc finger protein 185	4,437	1,929	4,084	1,765	2,907	1,239	10,638	4,741	2,035	0,960	4,415	2,050		
(74)	Q9NQ38	Serine protease inhibitor Kazal-type 5	7,098	1,886	4,084	1,078	6,541	1,703	21,277	5,795	1,017	0,293	6,623	1,880	5,450	1,373
(75)	P27482	Calmodulin-like protein 3	1,775	1,886	3,404	3,595	2,907	3,028			5,086	5,869	3,679	4,177	4,087	4,120
(76)	P62081	40S ribosomal protein S7	1,775	1,886	0,681	0,719	0,727	0,757								
(77)	P06731	Carcinoembryonic antigen-related cell adhesion molecule 5	2,662	1,819	1,361	0,924	1,453	0,973							4,087	2,649
(78)	Q6XPR3	Repetin	2,662	1,819	3,404	2,311	2,180	1,460								
(79)	P63104	14-3-3 protein zeta/delta	2,662	1,698	1,361	0,863	2,180	1,363			2,035	1,408	1,472	1,002		
(80)	P17931	Galectin-3	1,775	1,698	4,084	3,882	2,180	2,044			1,017	1,056	0,736	0,752	2,725	2,472
(81)	P99999	Cytochrome c	0,887	1,698	1,361	2,588	0,727	1,363			1,017	2,113	0,736	1,504	1,362	2,472
(82)	P05387	60S acidic ribosomal protein P2	0,887	1,698	1,361	2,588	0,727	1,363	2,660	5,215			0,736	1,504		
(83)	P46776	60S ribosomal protein L27a	0,887	1,698	0,681	1,294	1,453	2,725			1,017	2,113	0,736	1,504		
(84)	P01009	Alpha-1-antitrypsin precursor	3,549	1,617	2,042	0,924	2,180	0,973	5,319	2,483	1,017	0,503	2,208	1,074	1,362	0,589
(85)	Q8N1A0	Keratin-like protein KRT222	2,662	1,498	2,723	1,522	4,360	2,405								
(86)	P02787	Serotransferrin	5,324	1,455	8,169	2,218	9,448	2,531			7,121	2,113	5,151	1,504	5,450	1,413
(87)	P18510	Interleukin 1 receptor antagonist protein	0,887	1,415	1,361	2,157					3,052	5,282	2,208	3,759		
(88)	P16401	Histone H1.5 (Histone H1a)	0,887	1,415	0,681	1,078					1,017	1,761	0,736	1,253	1,362	2,060
(89)	P62847	40S ribosomal protein S24	0,887	1,415	0,681	1,078	0,727	1,136			1,017	1,761	0,736	1,253	1,362	2,060
(90)	P02749	Apolipoprotein H (beta-2-glycoprotein I)	1,775	1,415	2,042	1,618	2,907	2,271			1,017	0,880	0,736	0,627	1,362	1,030
(91)	Q92876	Kallikrein-6 precursor	1,775	1,415	2,723	2,157	2,907	2,271			1,017	0,880	0,736	0,627		
(92)	Q9H1E1	Ribonuclease 7 precursor	0,887	1,415	1,361	2,157	1,453	2,271							1,362	2,060
(93)	Q8NHM4	Putative trypsin-6	0,887	1,415	0,681	1,078	1,453	2,271								
(94)	Q96FQ6	Protein S100-A16	0,887	1,415	4,084	6,470	4,360	6,814								
(95)	P04279	Semenogelin-1	2,662	1,273	2,042	0,971	4,360	2,044								
(96)	Q02878	60S ribosomal protein L6	1,775	1,213	1,361	0,924	2,180	1,460			2,035	1,509	1,472	1,074		
(97)	P17900	Ganglioside GM2 activator precursor	0,887	1,213	0,681	0,924	1,453	1,947								
(98)	P67936	Tropomyosin alpha 4 chain	1,775	1,132	3,404	2,157	4,360	2,725							1,362	0,824
(99)	P02647	Apolipoprotein A1	1,775	1,061							14,242	9,243	10,302	6,578	4,087	2,318

Accession no		Protein Description	Sample A1		Sample A2		Sample A3		Sample Af		Sample Ar		Sample Af+r		Sample B	
			NSCF	NSAF	NSCF	NSAF	NSCF	NSAF	NSCF	NSAF	NSCF	NSAF	NSCF	NSAF	NSCF	NSAF
(100)	P20930	Filaggrin	4,437	1,061	3,404	0,809	2,180	0,511							5,450	1,236
(101)	P30043	Flavin reductase	0,887	1,061	1,361	1,618	0,727	0,852							1,362	1,545
(102)	Q09666	Neuroblast differentiation-associated protein AHNAK	25,732	1,030	50,374	2,003	38,517	1,511	21,277	0,873	28,484	1,238	26,490	1,132	13,624	0,517
(103)	P15311	Ezrin	3,549	1,029	3,404	0,980	4,360	1,239			1,017	0,320	0,736	0,228	1,362	0,375
(104)	P30086	Phosphatidylethanolamine-binding protein	0,887	0,943	1,361	1,438			2,660	2,897	2,035	2,347	2,208	2,506	2,725	2,747
(105)	Q9UKR3	Kallikrein 13 precursor	0,887	0,943	1,361	1,438	2,180	2,271			2,035	2,347	1,472	1,671		
(106)	P12273	Prolactin-inducible protein precursor	0,887	0,943	0,681	0,719					1,017	1,174	0,736	0,835		
(107)	Q16610	Extracellular matrix protein 1 precursor	2,662	0,943	3,404	1,198	4,360	1,514			1,017	0,391	0,736	0,278		
(108)	P09497	Clathrin light chain B	0,887	0,943	0,681	0,719									1,362	1,373
(109)	Q16629	Splicing factor, arginine/serine-rich 7	0,887	0,943												
(110)	P55145	ARMET protein precursor	0,887	0,943	0,681	0,719										
(111)	O95171	Sciellin	3,549	0,893	6,127	1,532	5,087	1,255			3,052	0,834	2,208	0,594	8,174	1,952
(112)	P02763	Alpha-1-acid glycoprotein 1	0,887	0,849	2,042	1,941	1,453	1,363								
(113)	P10909	Clusterin precursor	1,775	0,808	1,361	0,616	1,453	0,649	2,660	1,242			0,736	0,358		
(114)	P35579	Myosin-9	7,986	0,780	6,127	0,594	4,360	0,417	7,979	0,798	1,017	0,108	2,943	0,307	1,362	0,126
(115)	P06733	Alpha-enolase	1,775	0,772	2,723	1,176	2,907	1,239	2,660	1,185	2,035	0,960	2,208	1,025		
(116)	P62269	40S ribosomal protein S18	0,887	0,772	1,361	1,176	1,453	1,239			1,017	0,960	0,736	0,683		
(117)	Q9UBX7	Kallikrein 11 precursor	0,887	0,707	2,042	1,618	1,453	1,136			2,035	1,761	1,472	1,253	4,087	3,090
(118)	P23142	Fibulin-1 precursor	1,775	0,707	0,681	0,270	0,727	0,284			1,017	0,440	0,736	0,313		
(119)	P08107	Heat shock 70 kDa protein 1	1,775	0,679	2,723	1,035	2,180	0,818	7,979	3,129	10,173	4,225	9,566	3,909	2,725	0,989
(120)	P02545	Lamin-A/C	2,662	0,670	2,042	0,511	2,180	0,538			1,017	0,278	0,736	0,198		
(121)	P22735	Protein-glutamine gamma-glutamyltransferase K	2,662	0,670	1,361	0,341	0,727	0,179								
(122)	Q6E0U4	Dermokine precursor	0,887	0,653												
(123)	P14618	Pyruvate kinase isozymes M1/M2	1,775	0,585	0,681	0,223	2,180	0,705	2,660	0,899	5,086	1,821	4,415	1,555		
(124)	P15924	Desmoplakin	8,873	0,548	6,807	0,417	6,541	0,396	7,979	0,505	6,104	0,409	6,623	0,437	5,450	0,319
(125)	O60664	Mannose-6-phosphate receptor binding protein 1	0,887	0,499												
(126)	P39023	60S ribosomal protein L3	0,887	0,472											1,362	0,687
(127)	Q9UIV8	Serpin B13	0,887	0,447	0,681	0,341	0,727	0,359	2,660	1,372	4,069	2,224	3,679	1,978	2,725	1,301
(128)	P02774	Vitamin D-binding protein precursor	0,887	0,424	0,681	0,324	0,727	0,341								
(129)	Q92817	Envoplakin	4,437	0,408	6,127	0,560	7,267	0,655			2,035	0,203	1,472	0,145	1,362	0,119
(130)	P02790	Hemopexin	0,887	0,404	0,681	0,308	0,727	0,324			1,017	0,503	0,736	0,358	1,362	0,589
(131)	Q02487	Desmocollin-2 precursor	0,887	0,386	0,681	0,294	1,453	0,619			1,017	0,480	0,736	0,342	1,362	0,562
(132)	P20810	Calpastatin (Calpain inhibitor) (Sperm BS-17 component)	0,887	0,386	1,361	0,588	1,453	0,619							1,362	0,562
(133)	Q02383	Semenogelin-2	0,887	0,386	3,404	1,470	2,180	0,929								
(134)	Q14134	Tripartite motif-containing protein 29	0,887	0,326	2,042	0,747	0,727	0,262	2,660	1,003			0,736	0,289	2,725	0,951
(135)	P98187	Cytochrome P450 4F8	0,887	0,314			0,727	0,252								
(136)	P08670	Vimentin	0,887	0,293												
(137)	P18054	Arachidonate 12-lipoxygenase, 12S-type	0,887	0,265			2,180	0,639							2,725	0,773
(138)	P02671	Fibrinogen alpha chain precursor	0,887	0,250	1,361	0,381	1,453	0,401			1,017	0,311	0,736	0,221		

Accession no		Protein Description	Sample A1		Sample A2		Sample A3		Sample Af		Sample Ar		Sample Af+r		Sample B	
			NSCF	NSAF	NSCF	NSAF	NSCF	NSAF	NSCF	NSAF	NSCF	NSAF	NSCF	NSAF	NSCF	NSAF
(139)	P08123	Collagen alpha 2 T	0,887	0,236	0,681	0,180	0,727	0,189							2,725	0,687
(140)	Q13835	Plakophilin 1	0,887	0,193			0,727	0,155								
(141)	Q8TER0	Sushi, nidogen and EGF-like domain-containing protein 1	0,887	0,189												
(142)	P08603	Complement factor H	0,887	0,152	1,361	0,231										
(143)	P18206	Vinculin	0,887	0,149	1,361	0,227	0,727	0,120								
(144)	Q12888	Tumor suppressor p53-binding protein 1	0,887	0,133												
(145)	Q9HC84	Mucin-5B precursor	0,887	0,088	2,042	0,202	1,453	0,142	2,660	0,272	1,017	0,110	1,472	0,157	8,174	0,773
(146)	Q5TZA2	Rootletin	0,887	0,082	0,681	0,063										
(147)	Q15149	Plectin-1	0,887	0,035	2,042	0,079	0,727	0,028								
(148)	P62736	Actin, aortic smooth muscle							45,213	26,076			12,509	7,518		
(149)	Q06830	Peroxiredoxin 1					1,453	1,239	7,979	7,112	7,121	6,722	7,358	6,835	4,087	3,371
(150)	P01859	Ig gamma-2 chain C region					5,087	4,770	5,319	5,215	7,121	7,395	6,623	6,766	4,087	3,708
(151)	Q71DI3	Histone H3.2									9,156	23,768	6,623	16,916		
(152)	P59666	Neutrophil defensin 3 precursor									7,121	14,789	5,151	10,525		
(153)	Q7L7L0	Histone H2A type 3									7,121	24,649	5,151	17,542		
(154)	P05164	Myeloperoxidase precursor									6,104	1,585	4,415	1,128	2,725	0,618
(155)	A8K2U0	Alpha-2-macroglobulin-like protein 1 precursor			0,681	0,120	1,453	0,252			5,086	0,978	3,679	0,696	4,087	0,687
(156)	Q8NHS3	Major facilitator superfamily domain-containing protein 8			0,681	0,719			5,319	5,795	3,052	3,521	3,679	4,177	1,362	1,373
(157)	P68104	eukaryotic translation elongation factor 1 alpha 1									5,086	3,773	3,679	2,685		
(158)	Q99880	Histone H2B.c							10,638	20,861			2,943	6,014		
(159)	P00338	L-lactate dehydrogenase A chain									4,069	2,347	2,943	1,671		
(160)	P00558	Phosphoglycerate kinase 1									4,069	1,690	2,943	1,203		
(161)	P31947	Stratifin; 14-3-3 protein sigma									4,069	4,225	2,943	3,007		
(162)	O43707	Actinin, alpha 4			0,681	0,122					4,069	0,797	2,943	0,567		
(163)	P30041	Peroxiredoxin-6			2,042	1,294	1,453	0,908			4,069	2,817	2,943	2,005		
(164)	P30740	Monocyte/neutrophil elastase inhibitor			0,681	0,359			2,660	1,449	2,035	1,174	2,208	1,253	4,087	2,060
(165)	P54108	Cysteine-rich secretory protein 3			0,681	1,078	0,727	1,136	2,660	4,346	2,035	3,521	2,208	3,759	2,725	4,120
(166)	Q8IUE6	Histone H2A type 2-B									3,052	15,846	2,208	11,277	2,725	12,360
(167)	P14174	Macrophage migration inhibitory factor							2,660	8,692	2,035	7,042	2,208	7,518		
(168)	P48594	Squamous cell carcinoma antigen 2; Serpin B4			2,042	0,971	2,907	1,363	2,660	1,304	2,035	1,056	2,208	1,128		
(169)	P62249	40S ribosomal protein S16									3,052	3,961	2,208	2,819		
(170)	Q08380	Galectin-3-binding protein precursor									3,052	1,320	2,208	0,940		
(171)	Q14116	Interleukin-18 precursor									3,052	4,527	2,208	3,222		
(172)	P12036	Neurofilament heavy polypeptide							5,319	2,267			1,472	0,654		
(173)	P54652	Heat shock-related 70 kDa protein 2							5,319	1,932			1,472	0,557		
(174)	P62851	40S ribosomal protein S25							5,319	13,038			1,472	3,759		
(175)	Q15056	Eukaryotic translation initiation factor 4H							5,319	4,741			1,472	1,367		
(176)	Q9NYK1	Toll-like receptor 7 precursor							5,319	1,185			1,472	0,342		
(177)	Q9UJY1	Heat shock protein beta-8							5,319	6,519			1,472	1,880		

Accession no		Protein Description	Sample A1		Sample A2		Sample A3		Sample Af		Sample Ar		Sample Af+r		Sample B	
			NSCF	NSAF	NSCF	NSAF	NSCF	NSAF	NSCF	NSAF	NSCF	NSAF	NSCF	NSAF	NSCF	NSAF
(178)	P62937	Peptidyl-prolyl cis-trans isomerase A (Cyclophilin A)			2,042	2,426	1,453	1,703	5,319	6,519			1,472	1,880		
(179)	P11142	Heat shock 70kDa protein 8							2,660	1,003	1,017	0,406	1,472	0,578		
(180)	P00915	Carbonic anhydrase 1									2,035	1,921	1,472	1,367		
(181)	P02652	Apolipoprotein A-II precursor									2,035	5,282	1,472	3,759		
(182)	P09211	Glutathione S-transferase P									2,035	2,113	1,472	1,504		
(183)	P13797	Plastin-3									2,035	0,660	1,472	0,470		
(184)	P69891	Hemoglobin subunit gamma-1									2,035	1,761	1,472	1,253		
(185)	O75369	Filamin-B			0,681	0,054					2,035	0,178	1,472	0,126		
(186)	P08246	Leukocyte elastase precursor			0,681	1,078					2,035	3,521	1,472	2,506		
(187)	P02100	Hemoglobin subunit epsilon			2,723	2,157	2,180	1,703			2,035	1,761	1,472	1,253		
(188)	P61254	60S ribosomal protein L26			0,681	0,924	1,453	1,947			2,035	3,018	1,472	2,148		
(189)	O60235	Transmembrane protease, serine 11D precursor									1,017	2,641	0,736	1,880	4,087	9,270
(190)	Q99835	Smoothened homolog precursor							2,660	1,087			0,736	0,313	1,362	0,515
(191)	P00738	Haptoglobin									1,017	0,587	0,736	0,418	1,362	0,687
(192)	Q9UL52	Transmembrane protease, serine 11E									1,017	0,587	0,736	0,418	1,362	0,687
(193)	O00555	Voltage-dependent P/Q-type calcium channel subunit alpha-1A							2,660	0,362			0,736	0,104		
(194)	P04114	Apolipoprotein B-100 precursor							2,660	0,113			0,736	0,033		
(195)	P15056	B-Raf proto-oncogene serine/threonine-protein kinase							2,660	0,966			0,736	0,278		
(196)	P19447	TFIIH basal transcription factor complex helicase XPB subunit							2,660	0,745			0,736	0,215		
(197)	P80511	Protein S100-A12							2,660	8,692			0,736	2,506		
(198)	Q16825	Tyrosine-protein phosphatase non-receptor type 21							2,660	0,579			0,736	0,167		
(199)	Q5TZ20	Olfactory receptor 2G6							2,660	5,215			0,736	1,504		
(200)	Q6ZVX7	Putative uncharacterized protein LOC342897							2,660	2,371			0,736	0,683		
(201)	Q8NA31	Coiled-coil domain-containing protein 79							2,660	0,869			0,736	0,251		
(202)	Q8NCR0	UDP-GalNAc:beta-1,3-N-acetylgalactosaminyltransferase 2							2,660	1,372			0,736	0,396		
(203)	Q8TC20	Cancer-associated gene 1 protein							2,660	0,899			0,736	0,259		
(204)	Q8TER5	Protein SOLO							2,660	0,450			0,736	0,130		
(205)	Q8WXH0	Nesprin-2							2,660	0,070			0,736	0,020		
(206)	Q96FF9	Sororin							2,660	2,371			0,736	0,683		
(207)	Q9BYE4	Small proline-rich protein 2G							2,660	8,692			0,736	2,506		
(208)	Q9C0A6	SET domain-containing protein 5							2,660	0,511			0,736	0,147		
(209)	Q9UPQ7	PDZ domain-containing RING finger protein 3							2,660	0,652			0,736	0,188		
(210)	Q9Y3T6	R3H and coiled-coil domain-containing protein 1							2,660	1,185			0,736	0,342		
(211)	Q8WWI1	LIM domain only protein 7			0,681	0,069			2,660	0,277			0,736	0,080		
(212)	O43175	D-3-phosphoglycerate dehydrogenase									1,017	0,528	0,736	0,376		
(213)	P00918	Carbonic anhydrase 2									1,017	0,880	0,736	0,627		
(214)	P01033	Metalloproteinase inhibitor 1 precursor									1,017	1,174	0,736	0,835		
(215)	P01605	Ig kappa chain V-I region Lay									1,017	5,282	0,736	3,759		
(216)	P02675	Fibrinogen beta chain precursor									1,017	0,406	0,736	0,289		

Accession no		Protein Description	Sample A1		Sample A2		Sample A3		Sample Af		Sample Ar		Sample Af+r		Sample B	
			NSCF	NSAF	NSCF	NSAF	NSCF	NSAF	NSCF	NSAF	NSCF	NSAF	NSCF	NSAF	NSCF	NSAF
(217)	P04433	Ig kappa chain V-III region VG precursor									1,017	3,521	0,736	2,506		
(218)	P06744	Glucose-6-phosphate isomerase									1,017	0,480	0,736	0,342		
(219)	P07951	Tropomyosin beta chain									1,017	0,880	0,736	0,627		
(220)	P08238	Heat shock protein HSP 90-beta									1,017	0,293	0,736	0,209		
(221)	P08708	40S ribosomal protein S17									1,017	2,113	0,736	1,504		
(222)	P09466	Glycodelin precursor									1,017	1,320	0,736	0,940		
(223)	P09651	Heterogeneous nuclear ribonucleoprotein A1									1,017	0,880	0,736	0,627		
(224)	P10412	Histone H1.4									1,017	1,761	0,736	1,253		
(225)	P10606	Cytochrome c oxidase subunit 5B, mitochondrial precursor									1,017	1,761	0,736	1,253		
(226)	P12429	Annexin A3									1,017	0,528	0,736	0,376		
(227)	P13796	Lymphocyte cytosolic protein 1 (L-plastin)									1,017	0,311	0,736	0,221		
(228)	P18669	Phosphoglycerate mutase 1									1,017	1,056	0,736	0,752		
(229)	P25685	DnaJ homolog subfamily B member 1									1,017	0,660	0,736	0,470		
(230)	P29373	Cellular retinoic acid-binding protein 2									1,017	1,320	0,736	0,940		
(231)	P35268	60S ribosomal protein L22									1,017	2,641	0,736	1,880		
(232)	P52209	6-phosphogluconate dehydrogenase, decarboxylating									1,017	0,556	0,736	0,396		
(233)	P62266	40S ribosomal protein S23									1,017	2,641	0,736	1,880		
(234)	P62280	40S ribosomal protein S11									1,017	1,174	0,736	0,835		
(235)	P62753	40S ribosomal protein S6									1,017	1,320	0,736	0,940		
(236)	P63220	40S ribosomal protein S21									1,017	2,113	0,736	1,504		
(237)	P68366	Tubulin alpha-1 chain									1,017	0,556	0,736	0,396		
(238)	Q06323	Proteasome activator complex subunit 1									1,017	0,704	0,736	0,501		
(239)	Q07065	Cytoskeleton associated protein 4									1,017	0,330	0,736	0,235		
(240)	Q08188	Protein-glutamine gamma-glutamyltransferase E precursor									1,017	0,556	0,736	0,396		
(241)	Q8IUS5	Abhydrolase domain-containing protein 7									1,017	0,660	0,736	0,470		
(242)	Q8IZQ1	WD repeat and FYVE domain-containing protein 3									1,017	0,076	0,736	0,054		
(243)	Q92765	Secreted frizzled-related protein 3 precursor									1,017	1,509	0,736	1,074		
(244)	Q96HE7	ERO1-like protein alpha precursor									1,017	0,440	0,736	0,313		
(245)	Q9Y446	Plakophilin-3									1,017	0,311	0,736	0,221		
(246)	P40121	Macrophage-capping protein			0,681	0,539					1,017	0,880	0,736	0,627		
(247)	P31997	Carcinoembryonic antigen-related cell adhesion molecule 8			0,681	0,719					1,017	1,174	0,736	0,835		
(248)	P62854	40S ribosomal protein S26			0,681	1,294					1,017	2,113	0,736	1,504		
(249)	P07737	Profilin 1			1,361	1,618					1,017	1,320	0,736	0,940		
(250)	Q96P63	Serpin B12			0,681	0,381	0,727	0,401			1,017	0,621	0,736	0,442		
(251)	P50914	60S ribosomal protein L14					0,727	0,852			1,017	1,320	0,736	0,940		
(252)	P62899	60S ribosomal protein L31			0,681	1,078	1,453	2,271			1,017	1,761	0,736	1,253		
(253)	P49773	Histidine triad nucleotide-binding protein 1			0,681	1,294	1,453	2,725			1,017	2,113	0,736	1,504		
(254)	O60814	Histone H2B type 1-K			6,807	12,940	6,541	12,265							13,624	24,720
(255)	P01877	Ig alpha-2 chain C region			5,446	5,751									10,899	10,987

Accession no		Protein Description	Sample A1		Sample A2		Sample A3		Sample Af		Sample Ar		Sample Af+r		Sample B	
			NSCF	NSAF	NSCF	NSAF	NSCF	NSAF	NSCF	NSAF	NSCF	NSAF	NSCF	NSAF	NSCF	NSAF
(256)	P22532	Small proline-rich protein 2D					3,634	8,517							6,812	15,450
(257)	P16403	Histone H1.2													2,725	4,120
(258)	P26373	60S ribosomal protein L13			0,681	0,809	0,727	0,852							2,725	3,090
(259)	P62158	Calmodulin			1,361	1,618	0,727	0,852							2,725	3,090
(260)	O94823	Probable phospholipid-transporting ATPase VB													1,362	0,258
(261)	P01861	Ig gamma-4 chain C region													1,362	1,124
(262)	P05154	Plasma serine protease inhibitor precursor													1,362	0,687
(263)	P12814	Alpha-actinin-1													1,362	0,242
(264)	P15104	Glutamine synthetase													1,362	1,030
(265)	P62829	60S ribosomal protein L23													1,362	1,545
(266)	P62861	40S ribosomal protein S30													1,362	6,180
(267)	P62910	60S ribosomal protein L32													1,362	1,766
(268)	Q14210	Lymphocyte antigen 6D precursor													1,362	6,180
(269)	Q15847	Adipose most abundant gene transcript 2 protein													1,362	6,180
(270)	Q6P3W6	Neuroblastoma breakpoint family member 10													1,362	0,441
(271)	Q71UM5	40S ribosomal protein S27-like protein													1,362	4,120
(272)	Q86SG5	Protein S100-A7-like 1													1,362	2,472
(273)	Q8NAC3	Interleukin-17 receptor C precursor													1,362	0,458
(274)	Q8TD31	Coiled-coil alpha-helical rod protein 1													1,362	0,325
(275)	Q93100	Phosphorylase b kinase regulatory subunit beta													1,362	0,258
(276)	Q9HAY6	Beta,beta-carotene 15,15'-monooxygenase													1,362	0,475
(277)	P01591	Immunoglobulin J chain													1,362	1,766
(278)	Q9H7D7	WD repeat-containing protein 26													1,362	0,494
(279)	Q8NGC9	Olfactory receptor 11H4			0,681	1,078									1,362	2,060
(280)	P47914	60S ribosomal protein L29			0,681	3,235									1,362	6,180
(281)	Q01518	CAP, adenylate cyclase-associated protein 1 (yeast)					0,727	0,273							1,362	0,494
(282)	P60174	Triosephosphate isomerase			0,681	0,462	0,727	0,487							1,362	0,883
(283)	Q5CZC0	Fibrous sheath-interacting protein 2			0,681	0,047										
(284)	Q8IVV2	Lipoxygenase homology domain-containing protein 1			0,681	0,080										
(285)	Q5T0Z8	Uncharacterized protein C6orf132			0,681	0,129										
(286)	Q7Z406	myosin, heavy chain 14 isoform 1			1,361	0,129										
(287)	P32926	Desmoglein-3			0,681	0,185										
(288)	Q01954	Zinc finger protein basonuclin-1			0,681	0,196										
(289)	Q00796	Sorbitol dehydrogenase			0,681	0,341										
(290)	Q9BQE3	Tubulin alpha-1C chain			0,681	0,341										
(291)	P31946	14-3-3 protein beta/alpha			0,681	0,462										
(292)	P15259	Phosphoglycerate mutase 2			0,681	0,539										
(293)	P16035	Metalloproteinase inhibitor 2			0,681	0,647										
(294)	P23528	cofilin-1			0,681	0,647										

Accession no		Protein Description	Sample A1		Sample A2		Sample A3		Sample Af		Sample Ar		Sample Af+r		Sample B	
			NSCF	NSAF	NSCF	NSAF	NSCF	NSAF	NSCF	NSAF	NSCF	NSAF	NSCF	NSAF	NSCF	NSAF
(295)	P62906	60S ribosomal protein L10a			0,681	0,647										
(296)	P02765	Alpha-2-HS-glycoprotein			0,681	0,719										
(297)	P00441	Superoxide dismutase [Cu-Zn]			0,681	1,294										
(298)	P02766	Transthyretin			0,681	1,294										
(299)	Q9GZV4	Eukaryotic translation initiation factor 5A-2			0,681	1,294										
(300)	P39019	40S ribosomal protein S19			1,361	1,438										
(301)	P07305	Histone H1.0			0,681	1,618										
(302)	Q9Y2V2	Calcium-regulated heat stable protein 1			1,361	2,157										
(303)	P60985	Keratinocyte differentiation-associated protein			0,681	3,235										
(304)	A8MQ03	UPF0574 protein C9orf169			1,361	3,235										
(305)	Q08EQ4	Thymosin beta-4-like protein 1			0,681	6,470										
(306)	P35325	Small proline-rich protein 2B			2,723	8,627										
(307)	Q9ULV0	Myosin-5B					0,727	0,074								
(308)	P01024	Complement component 3					0,727	0,077								
(309)	Q9Y4K1	Absent in melanoma 1 protein			0,681	0,104	0,727	0,110								
(310)	Q9BYT8	Neurolysin, mitochondrial					0,727	0,213								
(311)	Q9BW04	specifically androgen-regulated protein			0,681	0,249	0,727	0,262								
(312)	Q96HC4	PDZ and LIM domain protein 5			0,681	0,281	0,727	0,296								
(313)	P14384	Carboxypeptidase M			0,681	0,324	0,727	0,341								
(314)	O15144	Actin-related protein 2/3 complex subunit 2			0,681	0,341	0,727	0,359								
(315)	P23786	Carnitine O-palmitoyltransferase 2, mitochondrial					1,453	0,379								
(316)	A6NL28	Putative tropomyosin alpha-3 chain-like protein			1,361	0,863	0,727	0,454								
(317)	P54253	Ataxin-1					1,453	0,545								
(318)	Q9P0G3	Kallikrein-14			1,361	1,078	0,727	0,568								
(319)	Q3KQU3	MAP7 domain-containing protein 1			0,681	0,270	1,453	0,568								
(320)	Q92597	Protein NDRG1					0,727	0,757								
(321)	P18621	60S ribosomal protein L17 (L23) isoform 5			0,681	0,809	0,727	0,852								
(322)	P46778	60S ribosomal protein L21			0,681	0,924	0,727	0,973								
(323)	P0C869	Cytosolic phospholipase A2 beta					2,907	0,973								
(324)	P83731	60S ribosomal protein L24					0,727	1,136								
(325)	P84103	Splicing factor, arginine/serine-rich 3					0,727	1,136								
(326)	P62917	60S ribosomal protein L8			1,361	2,157	0,727	1,136								
(327)	P62424	60S ribosomal protein L7a			2,042	1,765	1,453	1,239								
(328)	P01034	Cystatin-C					0,727	1,363								
(329)	P61769	Beta-2-microglobulin					0,727	1,363								
(330)	P62263	40S ribosomal protein S14					0,727	1,703								
(331)	P32320	Cytidine deaminase			0,681	1,618	0,727	1,703								
(332)	P42766	60S ribosomal protein L35			0,681	1,618	0,727	1,703								
(333)	Q15843	NEDD8					0,727	2,271								

Accession no		Protein Description	Sample A1		Sample A2		Sample A3		Sample Af		Sample Ar		Sample Af+r		Sample B	
			NSCF	NSAF	NSCF	NSAF	NSCF	NSAF	NSCF	NSAF	NSCF	NSAF	NSCF	NSAF	NSCF	NSAF
(334)	P14136	Glial fibrillary acidic protein			7,488	2,542	7,267	2,433								
(335)	Q5VTE0	Putative elongation factor 1-alpha-like 3			4,084	2,773	5,087	3,407								
(336)	P63313	Thymosin beta-10			0,681	6,470	0,727	6,814								
(337)	Q15651	High mobility nucleosome-binding domain-containing protein 3			0,681	6,470	0,727	6,814								
(338)	Q93077	Histone H2A type 1-C					2,907	9,085								
(339)	Q8N257	Histone H2B type 3-B			5,446	12,940	5,814	13,627								
