# Supplementary material for: Comprehensive proteomic analysis of human cervical-vaginal fluid using colposcopy samples
Source: Proteome Sci. 2009 Apr 17;7:17. doi: 10.1186/1477-5956-7-17 (PMC2678104; doi:10.1186/1477-5956-7-17)
Supplement: Additional file 7 — Overview of all identifications obtained in different human proteomics studies on human CVF. [file 1477-5956-7-17-S7.pdf]

Additional file 7 – Overview of all identifications obtained in different proteomics studies on human CVF.

Accession No	Protein Description	Dasari[1]	DiQuinzio[2]	Klein[3]	Pereira[4]	Shaw[5]	Tang[6]	Venkataraman[7]	This study
(1) 681073A	Haptoglobin alpha1S						√		
(2) A5YKK6	CCR4-NOT transcription complex, subunit 1 isoform a					√			
(3) A6NHG4	D-dopachrome decarboxylase-like protein					√			
(4) A6NL28	Putative tropomyosin alpha-3 chain-like protein								√
(5) A8K2U0	Alpha-2-macroglobulin-like protein 1 precursor					√			√
(6) A8MQ03	UPF0574 protein C9orf169								√
(7) A8MQC9	Uncharacterized protein CLCA4					√			
(8) A9Z1Y9	Thymosin beta-4-like protein 6								√
(9) BAC01816	Immunoglobulin λ light chain VLJ						√		
(10) O00151	PDZ and LIM domain protein 1					√			
(11) O00204	Sulfotransferase family cytosolic 2B member 1					√			
(12) O00299	Chloride intracellular channel protein 1						√		
(13) O00391	Sulfhydryl oxidase 1 precursor					√			
(14) O00555	Voltage-dependent P/Q-type calcium channel subunit alpha-1A								√
(15) O00584	Ribonuclease T2 precursor					√			
(16) O00754	mannosidase, alpha, class 2B, member 1 precursor					√			
(17) O14745	Ezrin-radixin-moesin-binding phosphoprotein 50					√			
(18) O15031	plexin-B2 precursor					√			
(19) O15143	Actin-related protein 2/3 complex subunit 1B								
(20) O15144	Actin-related protein 2/3 complex subunit 2								√
(21) O15145	Actin-related protein 2/3 complex subunit 3					√			
(22) O15231	Zinc finger protein 185					√			√
(23) O15260	Surfeit locus protein 4					√			
(24) O15263	Beta-defensin 2 precursor								√
(25) O15335	Chondroadherin precursor								
(26) O15393	Transmembrane protease, serine 2 precursor					√			
(27) O15400	syntaxin 7					√			
(28) O15457	MutS protein homolog 4					√			
(29) O15511	Actin-related protein 2/3 complex subunit 5								
(30) O43175	D-3-phosphoglycerate dehydrogenase					√			√
(31) O43240	Kallikrein-10 precursor					√			√
(32) O43278	Kunitz-type protease inhibitor 1 precursor					√			
(33) O43451	Maltase-glucoamylase, intestinal					√			
(34) O43497	Voltage-dependent T-type calcium channel subunit alpha-1G					√			
(35) O43707	Actinin, alpha 4	√			√	√			√
(36) O43781	Dual-specificity tyrosine-phosphorylation regulated kinase 3					√			
(37) O43852	Calumenin precursor					√			
(38) O43866	CD5 antigen-like precursor					√			
(39) O60218	Aldo-keto reductase family 1, member B10 (aldose reductase)				√	√			
(40) O60235	Transmembrane protease, serine 11D precursor	√			√	√			√
(41) O60259	Kallikrein-8 precursor					√			

Accession No		Protein Description	Dasari[1]	DiQuinzio[2]	Klein[3]	Pereira[4]	Shaw[5]	Tang[6]	Venkataraman[7]	This study
(42)	O60285-2	NUAK family SNF1-like kinase 1							√	
(43)	O60391	Glutamate [NMDA] receptor subunit 3B precursor					√			
(44)	O60437	Periplakin	√			√	√			√
(45)	O60504	Vinexin	√							
(46)	O60664	Mannose-6-phosphate receptor binding protein 1				√				√
(47)	O60814	Histone H2B type 1-K								√
(48)	O75015	Fc-gamma receptor IIIb					√			
(49)	O75083	WD repeat protein 1					√			
(50)	O75131	Copine-3				√				
(51)	O75223	Protein C7orf24	√			√	√			
(52)	O75351	Vacuolar protein sorting-associating protein 4B					√			
(53)	O75367	Core histone macro-H2A.1					√			
(54)	O75369	Filamin-B					√			√
(55)	O75448	Thyroid hormone receptor-associated protein complex 100 kDa component					√			
(56)	O75594	Peptidoglycan recognition protein precursor				√				
(57)	O75629	Protein CREG1 precursor					√			
(58)	O75882	Attractin precursor					√			
(59)	O94823	Probable phospholipid-transporting ATPase VB								√
(60)	O94919	Endonuclease domain-containing 1 protein precursor					√			
(61)	O95171	Sciellin	√			√	√			√
(62)	O95274	Ly6/PLAUR domain-containing protein 3 precursor					√			√
(63)	O95361	Tripartite motif-containing protein 16					√			
(64)	O95831	Programmed cell death protein 8, mitochondrial precursor					√			
(65)	O95994	Anterior gradient protein 2 homolog precursor								
(66)	P00338	L-lactate dehydrogenase A chain				√	√			√
(67)	P00354	Glyceraldehyde-3-phosphate dehydrogenase								
(68)	P00390	Glutathione reductase, mitochondrial precursor				√	√			
(69)	P00441	Superoxide dismutase [Cu-Zn]	√	√		√	√			√
(70)	P00450	Ceruloplasmin precursor	√			√	√	√		
(71)	P00491	Purine nucleoside phosphorylase				√	√	√		
(72)	P00492	Hypoxanthine-guanine phosphoribosyltransferase				√				
(73)	P00558	Phosphoglycerate kinase 1	√			√	√			√
(74)	P00738	Haptoglobin	√			√	√	√		√
(75)	P00746	Complement factor D precursor								
(76)	P00747	Plasminogen precursor	√				√			
(77)	P00748	Coagulation factor XII precursor					√			
(78)	P00751	Complement factor B precursor				√	√			
(79)	P00915	Carbonic anhydrase 1								√
(80)	P00918	Carbonic anhydrase 2								√
(81)	P01008	Antithrombin-III precursor	√				√	√		
(82)	P01009	Alpha-1-antitrypsin precursor	√	√	√	√	√			√
(83)	P01011	Alpha-1-antichymotrypsin precursor	√		√		√			
(84)	P01019	Angiotensinogen precursor [Contains: Angiotensin I				√				

Accession No		Protein Description	Dasari[1]	DiQuinzio[2]	Klein[3]	Pereira[4]	Shaw[5]	Tang[6]	Venkataraman[7]	This study
(85)	P01023	Alpha-2-macroglobulin precursor				√	√			
(86)	P01024	Complement component 3	√			√	√	√		√
(87)	P01028	Complement C4 precursor	√			√				
(88)	P01033	Metalloproteinase inhibitor 1 precursor								√
(89)	P01034	Cystatin-C								√
(90)	P01040	Cystatin A (stefin A)	√	√		√	√	√	√	√
(91)	P01042	Kininogen	√			√				
(92)	P01591	Immunoglobulin J chain	√			√	√			√
(93)	P01593	Ig kappa chain V-I					√			
(94)	P01597	Ig kappa chain V-I region DEE	√							
(95)	P01605	Ig kappa chain V-I region Lay								√
(96)	P01616	Ig kappa chain V-II region MIL					√			
(97)	P01617	Ig kappa chain V-II region TEW	√			√				
(98)	P01620	Ig kappa chain V-III region SIE	√							
(99)	P01625	Ig kappa chain V-IV region Len	√			√	√			
(100)	P01701	Ig lambda chain V-I region NEW				√				
(101)	P01703	Ig lambda chain V-I region NEWM	√							
(102)	P01708	Ig lambda chain V-II region BUR					√			
(103)	P01714	Ig lambda chain V-III region SH					√			
(104)	P01743	Ig heavy chain V-I region HG3 precursor					√			
(105)	P01766	Ig heavy chain V-III region BRO				√	√			
(106)	P01768	Ig heavy chain V-III region CAM					√			
(107)	P01771	Ig heavy chain V-III region HIL					√			
(108)	P01772	Ig heavy chain V-III region KOL				√				
(109)	P01833	Polymeric immunoglobulin receptor	√		√	√	√			√
(110)	P01834	Ig kappa chain C region	√		√	√	√			√
(111)	P01842	Ig lambda chain C regions	√		√	√	√			√
(112)	P01857	Ig gamma-1 chain C region	√		√	√	√	√		√
(113)	P01859	Ig gamma-2 chain C region	√			√	√			√
(114)	P01860	Ig gamma-3 chain C region				√				√
(115)	P01861	Ig gamma-4 chain C region	√			√	√			√
(116)	P01871	Ig mu chain C region	√			√	√			
(117)	P01876	Ig alpha-1 chain C region	√			√	√			√
(118)	P01877	Ig alpha-2 chain C region	√				√			√
(119)	P02042	Hemoglobin subunit delta								√
(120)	P02100	Hemoglobin subunit epsilon								√
(121)	P02511	Alpha-crystallin B chain					√			√
(122)	P02545	Lamin-A/C				√	√			√
(123)	P02647	Apolipoprotein A1	√			√	√	√		√
(124)	P02652	Apolipoprotein A-II precursor								√
(125)	P02671	Fibrinogen alpha chain precursor	√			√	√			√
(126)	P02675	Fibrinogen beta chain precursor	√			√	√	√		√
(127)	P02679	Fibrinogen gamma chain	√			√	√			

Accession No	Protein Description	Dasari[1]	DiQuinzio[2]	Klein[3]	Pereira[4]	Shaw[5]	Tang[6]	Venkataraman[7]	This study
(128) P02749	Apolipoprotein H (beta-2-glycoprotein I)	√			√	√			√
(129) P02750	Leucine-rich alpha-2-glycoprotein precursor				√				
(130) P02751	Fibronectin precursor				√	√			
(131) P02760	AMBP protein precursor [Contains: Alpha-1-microglobulin				√				
(132) P02763	Alpha-1-acid glycoprotein 1	√			√	√	√		√
(133) P02765	Alpha-2-HS-glycoprotein	√			√				√
(134) P02766	Transthyretin		√		√	√	√		√
(135) P02768	Serum albumin precursor	√	√	√	√	√	√	√	√
(136) P02774	Vitamin D-binding protein precursor	√			√	√	√		√
(137) P02787	Serotransferrin	√		√	√	√	√		√
(138) P02788	Lactotransferrin	√		√	√	√	√		√
(139) P02790	Hemopexin	√			√	√			√
(140) P02792	Ferritin light chain					√			
(141) P03973	Antileukoproteinase 1 precursor	√		√	√	√			√
(142) P04003	C4b-binding protein alpha chain precursor					√			
(143) P04004	Vitronectin	√			√				
(144) P04040	Catalase	√			√	√	√		
(145) P04075	Fructose-bisphosphate aldolase A	√			√	√			√
(146) P04080	Cystatin B	√			√	√	√	√	√
(147) P04083	Annexin A1	√		√	√	√	√		√
(148) P04114	Apolipoprotein B-100 precursor								√
(149) P04196	Histidine-rich glycoprotein precursor					√			
(150) P04207	Ig kappa chain V-III region CLL [Precursor]				√				
(151) P04208	Ig lambda chain V-I region WAH					√			
(152) P04217	Alpha-1-B glycoprotein				√		√		
(153) P04279	Semenogelin-1	√							√
(154) P04406	Glyceraldehyde-3-phosphate dehydrogenase, liver	√		√	√	√	√		√
(155) P04433	Ig kappa chain V-III region VG precursor								√
(156) P04792	Heat-shock protein beta-1	√		√	√	√			√
(157) P05089	Arginase-1					√			
(158) P05090	Apolipoprotein D precursor					√			
(159) P05107	Integrin beta-2 precursor					√			
(160) P05109	Calgranulin A (S100A8)	√		√	√	√	√	√	√
(161) P05154	Plasma serine protease inhibitor precursor					√			√
(162) P05155	Plasma protease C1 inhibitor precursor					√			
(163) P05164	Myeloperoxidase precursor	√		√	√	√			√
(164) P05204	Non-histone chromosomal protein HMG-17	√							√
(165) P05386	60S acidic ribosomal protein P1	√							
(166) P05387	60S acidic ribosomal protein P2	√							√
(167) P05388	60S acidic ribosomal protein P10	√							
(168) P06312	Ig kappa chain V-IV region precursor				√				
(169) P06396	Gelsolin				√	√			
(170) P06702	Calgranulin B (S100A9)	√		√	√	√	√	√	√

Accession No	Protein Description	Dasari[1]	DiQuinzio[2]	Klein[3]	Pereira[4]	Shaw[5]	Tang[6]	Venkataraman[7]	This study
(171) P06703	Protein S100-A6								
(172) P06731	Carcinoembryonic antigen-related cell adhesion molecule 5 precursor	√				√			√
(173) P06733	Alpha-enolase	√			√		√		√
(174) P06737	Glycogen phosphorylase, liver form								
(175) P06744	Glucose-6-phosphate isomerase								√
(176) P06753	Tropomyosin 3				√	√			√
(177) P07108	Acyl-CoA binding protein	√	√		√	√			√
(178) P07237	Protein disulfide-isomerase precursor	√			√	√			
(179) P07305	Histone H1.0								√
(180) P07339	Cathepsin D precursor				√	√			
(181) P07355	Annexin A2	√		√	√	√	√		√
(182) P07384	Calpain-1 catalytic subunit				√	√			
(183) P07476	Involucrin	√		√	√	√			√
(184) P07686	Beta-hexosaminidase beta chain precursor					√			
(185) P07737	Profilin 1	√			√	√	√		√
(186) P07858	Cathepsin B	√			√	√			
(187) P07900	Heat shock protein HSP 90-alpha 2	√			√	√			
(188) P07911	Uromodulin precursor					√			
(189) P07919	Ubiquinol-cytochrome c reductase complex 11 kDa protein, mitochondrial precursor					√			
(190) P07948	Tyrosine-protein kinase Lyn					√			
(191) P07951	Tropomyosin beta chain								√
(192) P07998	Ribonuclease pancreatic precursor					√			
(193) P08107	Heat shock 70 kDa protein 1	√			√	√			√
(194) P08123	Collagen alpha 2 T				√	√			√
(195) P08133	Annexin A6								
(196) P08174	Complement decay-accelerating factor precursor					√			
(197) P08236	Beta-glucuronidase precursor					√			
(198) P08238	Heat shock protein HSP 90-beta				√	√			√
(199) P08246	Leukocyte elastase precursor			√	√	√			√
(200) P08311	cathepsin G	√		√	√	√		√	√
(201) P08603	Complement factor H	√			√	√			√
(202) P08670	Vimentin	√			√	√			√
(203) P08697	Alpha-2-antiplasmin precursor					√			
(204) P08708	40S ribosomal protein S17								√
(205) P08758	Annexin A5; Calphobindin I				√		√		
(206) P08833	Insulin-like growth factor binding protein 1				√				
(207) P09211	Glutathione S-transferase P	√	√		√	√	√		√
(208) P09429	High mobility group protein B1					√			
(209) P09466	Glycodelin precursor								√
(210) P09493	Tropomyosin alpha-1 chain					√			
(211) P09497	Clathrin light chain B								√
(212) P09525	annexin IV					√			
(213) P09651	Heterogeneous nuclear ribonucleoprotein A1					√			√

Accession No	Protein Description	Dasari[1]	DiQuinzio[2]	Klein[3]	Pereira[4]	Shaw[5]	Tang[6]	Venkataraman[7]	This study
(214) P09668	Cathepsin H precursor					√			
(215) P09758	Tumor-associated calcium signal transducer 2 precursor					√			
(216) P09960	Leukotriene A-4 hydrolase					√			
(217) P09972	Fructose-bisphosphate aldolase C					√			
(218) P0C0L5	Complement C4-B precursor					√			
(219) P0C0S5	Histone H2A.Z (H2A/z).					√			
(220) P0C0S8	Histone H2A type 1								√
(221) P0C869	Cytosolic phospholipase A2 beta								√
(222) P10153	Nonsecretory ribonuclease precursor				√	√			
(223) P10155	60 kDa SS-A/Ro ribonucleoprotein					√			
(224) P10412	Histone H1.4								√
(225) P10599	Thioredoxin	√	√		√	√	√		√
(226) P10606	Cytochrome c oxidase subunit 5B, mitochondrial precursor								√
(227) P10619	Lysosomal protective protein precursor					√			
(228) P10909	Clusterin precursor					√			√
(229) P11021	78 kDa glucose-regulated protein	√			√	√			
(230) P11142	Heat shock 70kDa protein 8	√			√	√			√
(231) P11216	Glycogen phosphorylase, brain form					√			
(232) P11413	glucose-6-phosphate dehydrogenase isoform a					√	√		
(233) P12036	Neurofilament heavy polypeptide								√
(234) P12273	Prolactin-inducible protein precursor					√			√
(235) P12429	Annexin A3	√	√		√	√	√		√
(236) P12724	Eosinophil cationic protein precursor	√			√	√			√
(237) P12814	Alpha-actinin-1					√			√
(238) P12830	E-cadherin					√			
(239) P13611	Versican core protein precursor					√			
(240) P13639	Elongation factor 2	√			√	√			
(241) P13667	Protein disulfide-isomerase A4 precursor					√			
(242) P13671	Complement component C6 precursor					√			
(243) P13796	Lymphocyte cytosolic protein 1 (L-plastin)	√		√	√	√	√		√
(244) P13797	Plastin-3					√			√
(245) P13928	Annexin A8								
(246) P13929	Beta-enolase					√			
(247) P13987	CD59 glycoprotein precursor	√			√	√			√
(248) P14136	Glial fibrillary acidic protein								√
(249) P14174	Macrophage migration inhibitory factor								√
(250) P14314	Glucosidase 2 subunit beta precursor					√			
(251) P14317	Hematopoietic lineage cell-specific protein					√			
(252) P14384	Carboxypeptidase M								√
(253) P14550	Alcohol dehydrogenase [NADP+]								
(254) P14618	Pyruvate kinase isozymes M1/M2	√			√	√			√
(255) P14625	Endoplasmin precursor					√			
(256) P14780	Matrix metalloproteinase-9 precursor	√			√	√			

Accession No		Protein Description	Dasari[1]	DiQuinzio[2]	Klein[3]	Pereira[4]	Shaw[5]	Tang[6]	Venkataraman[7]	This study
(257)	P14854	Cytochrome c oxidase subunit VIb isoform 1					√			
(258)	P14923	Desmoplakin-3	√			√	√			
(259)	P15056	B-Raf proto-oncogene serine/threonine-protein kinase								√
(260)	P15104	Glutamine synthetase								√
(261)	P15153	Ras-related C3 botulinum toxin substrate 2 precursor					√			
(262)	P15170	Eukaryotic peptide chain release factor GTP-binding subunit ERF3A								
(263)	P15259	Phosphoglycerate mutase 2					√			√
(264)	P15289	Arylsulfatase A precursor					√			
(265)	P15309	Prostatic acid phosphatase precursor					√			
(266)	P15311	Ezrin					√			√
(267)	P15538	cytochrome P450, family 11, subfamily B, polypeptide 1 isoform 2 precursor					√			
(268)	P15924	Desmoplakin	√			√	√			√
(269)	P16035	Metalloproteinase inhibitor 2								√
(270)	P16401	Histone H1.5 (Histone H1a)	√			√	√			√
(271)	P16402	Histone H1.3	√							√
(272)	P16403	Histone H1.2				√				√
(273)	P16562	Cysteine-rich secretory protein 2 precursor								
(274)	P16870	Carboxipeptidase E						√		
(275)	P17213	bactericidal/permeability-increasing protein precursor					√			
(276)	P17858	liver phosphofructokinase isoform b					√			
(277)	P17900	Ganglioside GM2 activator precursor					√			√
(278)	P17931	Galectin-3					√			√
(279)	P18054	Arachidonate 12-lipoxygenase, 12S-type					√			√
(280)	P18136	Ig kappa chain V-III region HIC [Precursor]					√			
(281)	P18206	Vinculin	√			√	√			√
(282)	P18510	Interleukin 1 receptor antagonist protein	√	√		√	√	√		√
(283)	P18621	60S ribosomal protein L17 (L23) isoform 5					√			√
(284)	P18669	Phosphoglycerate mutase 1	√			√	√			√
(285)	P18858	DNA ligase 1					√			
(286)	P18859	ATP synthase, H+ transportine, mitochondrial FO complex,					√			
(287)	P19105	Myosin regulatory light chain 2, nonsarcomeric					√			
(288)	P19447	TFIIH basal transcription factor complex helicase XPB subunit								√
(289)	P19652	Alpha-1-acid glycoprotein 2 precursor				√	√			
(290)	P19957	Elafin precursor					√			√
(291)	P19961	Alpha-amylase 2B precursor					√			
(292)	P19971	Thymidine phosphorylase precursor								
(293)	P20020	Plasma membrane calcium-transporting ATPase1					√			
(294)	P20061	Transcobalamin-1 precursor					√			
(295)	P20160	Azurocidin 1 (cationic antimicrobial protein 37)				√	√			√
(296)	P20670	Histone H2A.o	√						√	
(297)	P20700	Lamin-B1					√			
(298)	P20810	Calpastatin (Calpain inhibitor) (Sperm BS-17 component)	√			√	√			√
(299)	P20908	Collagen alpha-1(V) chain precursor					√			

Accession No	Protein Description	Dasari[1]	DiQuinzio[2]	Klein[3]	Pereira[4]	Shaw[5]	Tang[6]	Venkataraman[7]	This study
(300) P20930	Filaggrin					√			√
(301) P21128	Placental protein 11 precursor					√			
(302) P21333	Filamin-A				√	√			
(303) P21817	Ryanodine receptor 1					√			
(304) P22090	40S ribosomal protein S4, Y isoform 1					√			
(305) P22314	Ubiquitin-activating enzyme E1					√			
(306) P22528	Cornifin B	√			√	√			√
(307) P22531	Small proline-rich protein 2E								√
(308) P22532	Small proline-rich protein 2D	√			√				√
(309) P22626	Heterogeneous nuclear ribonucleoprotein A2/B1					√			
(310) P22735	Protein-glutamine gamma-glutamyltransferase K	√				√			√
(311) P22894	Neutrophil collagenase precursor				√	√			
(312) P23083	Ig heavy chain V-I region V35 precursor				√				
(313) P23142	Fibulin-1 precursor					√			√
(314) P23246	Splicing factor, proline-and gluatmine-rich					√			
(315) P23284	peptidylprolyl isomerase B precursor					√			
(316) P23396	40S ribosomal protein S3					√			
(317) P23526	Adenosylhomocysteinase					√			
(318) P23528	cofilin-1	√				√			√
(319) P23786	Carnitine O-palmitoyltransferase 2, mitochondrial								√
(320) P24158	Myeloblastin precursor	√			√	√			√
(321) P25311	Alpha-2-glycoprotein 1, zinc					√	√		
(322) P25685	DnaJ homolog subfamily B member 1								√
(323) P25774	Cathepsin S precursor					√			
(324) P25789	Proteasome subunit alpha type 4					√			
(325) P25815	S100 calcium binding protein P				√	√			
(326) P26038	Moesin	√			√	√			
(327) P26373	60S ribosomal protein L13								√
(328) P26447	S100 calcium binding protein A4				√				
(329) P26583	High mobility group protein B2								
(330) P26641	Elongation factor 1-gamma					√			
(331) P27482	Calmodulin-like protein 3	√			√	√			√
(332) P27487	Dipeptidyl peptidase 4					√			
(333) P27797	Calreticulin precursor					√			
(334) P27816	Microtubule-associated protein 4	√							
(335) P27824	Calnexin precursor					√			
(336) P27918	Properdin precursor					√			
(337) P28001	Histone H2A.a				√				
(338) P28676	Grancalcin								
(339) P28799	Granulins precursor	√			√	√			
(340) P29034	Protein S100-A2				√				
(341) P29218	Inositol monophosphatase					√			
(342) P29373	Cellular retinoic acid-binding protein 2	√			√	√			√

Accession No		Protein Description	Dasari[1]	DiQuinzio[2]	Klein[3]	Pereira[4]	Shaw[5]	Tang[6]	Venkataraman[7]	This study
(343)	P29401	Transketolase				√	√	√		
(344)	P29508	Squamous cell carcinoma antigen 1 (SCCA-1); Serpin B3	√	√	√	√	√	√		√
(345)	P29590	Probable transcription factor PML					√			
(346)	P30041	Peroxiredoxin-6								√
(347)	P30043	Flavin reductase					√			√
(348)	P30044	Peroxiredoxin-5, mitochondrial precursor								
(349)	P30046	D-dopachrome tautomerase				√				
(350)	P30050	60S ribosomal protein L12					√			
(351)	P30085	UMP-CMP kinase								
(352)	P30086	Phosphatidylethanolamine-binding protein; Prostatic binding protein; neuropolypeptide h3	√			√	√	√		√
(353)	P30101	Glucose regulated protein, 58kDa; protein disulfide-isomerase A3	√							
(354)	P30153	Serine/threonine-protein phosphatase 2A 65 kDa regulatory subunit A					√			
(355)	P30456	HLA class I histocompatibility antigen, A-43 alpha chain precursor					√			
(356)	P30475	HLA class I histocompatibility antigen, B-39 alpha chain precursor					√			
(357)	P30740	Monocyte/neutrophil elastase inhibitor	√	√	√	√	√			√
(358)	P31146	Coronin, actin binding protein, 1A				√				
(359)	P31151	S100 calcium-binding protein A7 (psoriasin)	√			√	√			√
(360)	P31689	DnaJ homolog subfamily A member 1					√			
(361)	P31930	Ubiquinol-cytochrome-c reductase complex core protein I, mitochondrial precursor					√			
(362)	P31941	Probable DNA dC->dU-editing enzyme APOBEC-3A								
(363)	P31942	Heterogeneous nuclear ribonucleoprotein H3					√			
(364)	P31944	Caspase-14 precursor					√			
(365)	P31946	14-3-3 protein beta/alpha								√
(366)	P31947	Stratifin; 14-3-3 protein sigma	√			√	√			√
(367)	P31948	Stress-induced-phosphoprotein 1					√			
(368)	P31949	S100 calcium-binding protein A11 (calgizzarine)				√	√			√
(369)	P31997	Carcinoembryonic antigen-related cell adhesion molecule 8 precursor								√
(370)	P32119	Peroxiredoxin-2		√			√			√
(371)	P32243	Homeobox protein OTX2					√			
(372)	P32320	Cytidine deaminase				√	√	√		√
(373)	P32926	Desmoglein-3	√			√	√			√
(374)	P33241	Lymphocyte-specific protein 1					√			
(375)	P33778	Histone H2B.f				√				
(376)	P34059	N-acetylgalactosamine-6-sulfatase precursor					√			
(377)	P34932	Heat shock 70 kDa protein 4					√			
(378)	P35221	Catenin-alpha-1					√			
(379)	P35237	Serpin B6 (Placental thrombin inhibitor)	√				√			
(380)	P35268	60S ribosomal protein L22								√
(381)	P35321	Small-proline rich protein 1A; Cornifin A	√		√	√	√			√
(382)	P35325	Small proline-rich protein 2B	√							√
(383)	P35326	Small proline-rich protein 2A	√		√	√	√			√
(384)	P35555	Fibrillin-1 precursor					√			
(385)	P35579	Myosin-9				√	√			√

Accession No	Protein Description	Dasari[1]	DiQuinzio[2]	Klein[3]	Pereira[4]	Shaw[5]	Tang[6]	Venkataraman[7]	This study
(386) P35658	Nuclear pore complex protein Nup214					√			
(387) P35754	Glutaredoxin-1				√	√			
(388) P35789	Zinc finger protein 93					√			
(389) P36952	Maspin precursor; Serpin B5 precursor				√				
(390) P36955	Pigment epithelium-derived factor precursor								
(391) P36957	Dihydrolipoyllysine-residue succinyltransferase component					√			
(392) P37802	Transgelin-2				√	√			
(393) P37837	Transaldolase	√			√	√			
(394) P38159	RNA binding motif protein, X-linked-like 1					√			
(395) P38646	Heat shock 70 kDa protein 9					√			
(396) P39019	40S ribosomal protein S19								√
(397) P39023	60S ribosomal protein L3								√
(398) P39060	Type XVIII collagen long variant					√			
(399) P40121	Macrophage-capping protein								√
(400) P40925	Malate dehydrogenase, cytoplasmic								
(401) P40926	Malate dehydrogenase, mitochondrial precursor					√			
(402) P41439	folate receptor 3 precursor					√			
(403) P42677	40S ribosomal protein S27					√			
(404) P42766	60S ribosomal protein L35								√
(405) P43490	Nicotinamide phosphoribosyltransferase					√			
(406) P43652	Afamin precursor					√			
(407) P46108	Proto-oncogene c-crk					√			
(408) P46776	60S ribosomal protein L27a								√
(409) P46778	60S ribosomal protein L21								√
(410) P46821	Microtubule-associated protein 1B					√			
(411) P46940	Ras GTPase-activating-like protein IQGAP1					√			
(412) P46976	Glycogenin 1					√			
(413) P47756	F-actin cappling protein subunit beta				√	√			
(414) P47914	60S ribosomal protein L29								√
(415) P47929	Lectin, galactoside-binding, soluble, 7 (galectin 7)	√			√	√		√	√
(416) P48594	Squamous cell carcinoma antigen 2; Serpin B4	√			√	√	√		√
(417) P49189	aldehyde dehydrogenase 9A1					√			
(418) P49327	Fatty acid synthase					√			
(419) P49411	Tu translation elongation factor, mitochondrial					√			
(420) P49773	Histidine triad nucleotide-binding protein 1								√
(421) P49862	Kallikrein-7 precursor					√			
(422) P49913	Cathelicidin antimicrobial peptide precursor								√
(423) P50453	Serpin B9					√			
(424) P50750	Cell division protein kinase 9					√			
(425) P50914	60S ribosomal protein L14								√
(426) P50995	Annexin A11				√		√		
(427) P51149	Ras-related protein Rab-7				√				
(428) P51451	Tyrosine-protein kinase BLK								

Accession No		Protein Description	Dasari[1]	DiQuinzio[2]	Klein[3]	Pereira[4]	Shaw[5]	Tang[6]	Venkataraman[7]	This study
(429)	P51589	Cytochrome P450 2J2					√			
(430)	P51659	Peroxisomal multifunctional enzyme type 2					√			
(431)	P51805	Plexin-A3 precursor					√			
(432)	P51884	Lumican precursor				√				
(433)	P51991	Heterogeneous nuclear ribonucleoprotein A3					√			
(434)	P52209	6-phosphogluconate dehydrogenase, decarboxylating				√				√
(435)	P52565	Rho GDP dissociation inhibitor (GDI) alpha				√				
(436)	P52566	Rho GDP dissociation inhibitor (GDI) beta				√		√		
(437)	P52907	F-actin capping protein alpha-1 subunit				√	√	√		
(438)	P54108	Cysteine-rich secretory protein 3	√			√	√			√
(439)	P54253	Ataxin-1								√
(440)	P54652	Heat shock-related 70 kDa protein 2								√
(441)	P55000	Secreted Ly-6/uPAR-related protein 1 precursor					√			
(442)	P55072	Transitional endoplasmic reticulum ATPase					√			
(443)	P55145	ARMET protein precursor					√			√
(444)	P55196	Afadin (Protein AF-6)					√			
(445)	P55786	Puromycin-sensitive aminopeptidase				√	√			
(446)	P56537	Eukaryotic translation initiation factor 6					√			
(447)	P58062	Serine protease inhibitor Kazal-type 7 precursor					√			
(448)	P58546	Myotrophin				√	√			
(449)	P59665	Neutrophil defensin 1 precursor	√			√	√		√	√
(450)	P59666	Neutrophil defensin 3 precursor								√
(451)	P59998	Actin-related protein 2/3 complex subunit 4								
(452)	P60174	Triosephosphate isomerase	√			√	√			√
(453)	P60510	Serine/threonine-protein phosphatase 4 catalytic subunit					√			
(454)	P60660	Myosin light polypeptide 6								
(455)	P60709	Actin, cytoplasmic 1	√	√		√	√	√		√
(456)	P60866	40S ribosomal protein S20								√
(457)	P60900	Proteasome subunit alpha type 6				√				
(458)	P60903	S100 calcium binding protein A10; Calpactin I light chain				√	√			√
(459)	P60953	Cell division control protein 42 homolog precursor					√			
(460)	P60981	Destrin								
(461)	P60985	Keratinocyte differentiation-associated protein								√
(462)	P61088	Ubiquitin-conjugating enzyme E2 N								
(463)	P61158	Actin-related protein 3								
(464)	P61160	Actin-like protein 2				√	√			
(465)	P61254	60S ribosomal protein L26								√
(466)	P61457	Pterin-4-alpha-carbinolamine dehydratase					√			
(467)	P61586	Transforming protein RhoA				√				
(468)	P61626	Lysozym C	√		√	√	√	√	√	√
(469)	P61769	Beta-2-microglobulin								√
(470)	P61916	Epididymal secretory protein E1				√				
(471)	P61978	Heterogeneous nuclear ribonucleoprotein K					√			

Accession No		Protein Description	Dasari[1]	DiQuinzio[2]	Klein[3]	Pereira[4]	Shaw[5]	Tang[6]	Venkataraman[7]	This study
(472)	P62081	40S ribosomal protein S7								√
(473)	P62158	Calmodulin					√			√
(474)	P62195	Protease regulatory subunit 8, 26S						√		
(475)	P62244	40S ribosomal protein S15a					√			
(476)	P62249	40S ribosomal protein S16								√
(477)	P62263	40S ribosomal protein S14								√
(478)	P62266	40S ribosomal protein S23								√
(479)	P62269	40S ribosomal protein S18								√
(480)	P62280	40S ribosomal protein S11								√
(481)	P62318	Small nuclear ribonucleoprotein Sm D3					√			
(482)	P62328	Thymosin beta-4	√			√				
(483)	P62330	ADP-ribosylation factor 6					√			
(484)	P62424	60S ribosomal protein L7a								√
(485)	P62736	Actin, aortic smooth muscle								√
(486)	P62753	40S ribosomal protein S6					√			√
(487)	P62805	Histone H4	√		√	√	√		√	√
(488)	P62807	Histone H2B.a/g/h/k/l				√				
(489)	P62829	60S ribosomal protein L23								√
(490)	P62847	40S ribosomal protein S24								√
(491)	P62851	40S ribosomal protein S25								√
(492)	P62854	40S ribosomal protein S26								√
(493)	P62861	40S ribosomal protein S30								√
(494)	P62888	60S ribosomal protein L30					√			
(495)	P62899	60S ribosomal protein L31								√
(496)	P62906	60S ribosomal protein L10a								√
(497)	P62910	60S ribosomal protein L32								√
(498)	P62917	60S ribosomal protein L8								√
(499)	P62937	Peptidyl-prolyl cis-trans isomerase A (Cyclophilin A)	√			√		√		√
(500)	P62988	Ubiquitin	√			√	√			√
(501)	P63104	14-3-3 protein zeta/delta	√			√				√
(502)	P63167	Dynein light chain 1, cytoplasmic					√			
(503)	P63173	60S ribosomal protein L38								√
(504)	P63220	40S ribosomal protein S21								√
(505)	P63241	Eukaryotic initiation factor 5A isoform I variant A					√			
(506)	P63313	Thymosin beta-10				√				√
(507)	P67775	Serine/threonine-protein phosphatase 2A catalytic subunit alpha isoform					√			
(508)	P67936	Tropomyosin alpha 4 chain	√			√				√
(509)	P68032	Actin, alpha cardiac muscle 1								√
(510)	P68036	Ubiquitin-conjugating enzyme E2 L3				√				
(511)	P68104	eukaryotic translation elongation factor 1 alpha 1	√				√			√
(512)	P68366	Tubulin alpha-1 chain					√			√
(513)	P68431	Histone H3.1				√				
(514)	P68871	Hemoglobin beta chain			√	√	√			√

Accession No		Protein Description	Dasari[1]	DiQuinzio[2]	Klein[3]	Pereira[4]	Shaw[5]	Tang[6]	Venkataraman[7]	This study
(515)	P69891	Hemoglobin subunit gamma-1								√
(516)	P69905	Hemoglobin alpha subunit			√	√	√			√
(517)	P78417	Glutathione transferase omega 1				√				
(518)	P80188	Neutrophil gelatinase-associated lipocalin	√		√	√	√	√	√	√
(519)	P80511	Protein S100-A12				√	√	√		√
(520)	P80723	Brain abundant, membrane attached signal protein 1; Brain acid soluble protein 1	√			√				
(521)	P81605	Dermcidin precursor					√			√
(522)	P83731	60S ribosomal protein L24								√
(523)	P84103	Splicing factor, arginine/serine-rich 3								√
(524)	P98088	Mucin-5AC precursor				√	√			
(525)	P98187	Cytochrome P450 4F8								√
(526)	P99999	Cytochrome c					√			√
(527)	Q00610	clathrin heavy chain 1					√			
(528)	Q00688	FK506-binding protein 3					√			
(529)	Q00796	Sorbitol dehydrogenase								√
(530)	Q00839	heterogeneous nuclear ribonucleoprotein U isoform a					√			
(531)	Q01082	Spectrin beta chain, brain 1					√			
(532)	Q01105	Protein SET					√			
(533)	Q01459	Di-N-acetylchitobiase precursor					√			
(534)	Q01469	Fatty acid-binding protein, epidermal	√	√	√	√	√	√	√	√
(535)	Q01518	CAP, adenylate cyclase-associated protein 1 (yeast)				√				√
(536)	Q01954	Zinc finger protein basonuclin-1								√
(537)	Q02383	Semenogelin-2	√							√
(538)	Q02413	Desmoglein-1 precursor				√	√			
(539)	Q02487	Desmocollin-2 precursor	√			√	√			√
(540)	Q02818	Nucleobindin-1 precursor					√			
(541)	Q02878	60S ribosomal protein L6								√
(542)	Q03013	glutathione S-transferase M4 isoform 1					√			
(543)	Q03252	Lamin B2					√			
(544)	Q05524	Alpha-enolase, lung specific	√							
(545)	Q05639	Elongation factor 1-alpha 2								√
(546)	Q05682	Caldesmon					√			
(547)	Q06323	Proteasome activator complex subunit 1								√
(548)	Q06830	Peroxiredoxin 1	√			√	√			√
(549)	Q07065	Cytoskeleton associated protein 4					√			√
(550)	Q07157	Tight-junction protein ZO-1					√			
(551)	Q07654	trefoil factor 3 precursor					√			√
(552)	Q08188	Protein-glutamine gamma-glutamyltransferase E precursor					√			√
(553)	Q08380	Galectin-3-binding protein precursor					√			√
(554)	Q08EQ4	Thymosin beta-4-like protein 1								√
(555)	Q09666	Neuroblast differentiation-associated protein AHNAK	√		√	√	√			√
(556)	Q0VD83	apolipoprotein B48 receptor					√			
(557)	Q10588	ADP-ribosyl cyclase 2 precursor				√	√			

Accession No		Protein Description	Dasari[1]	DiQuinzio[2]	Klein[3]	Pereira[4]	Shaw[5]	Tang[6]	Venkataraman[7]	This study
(558)	Q12802	A-kinase anchor protein 13 (AKAP 13)					√			
(559)	Q12841	Follistatin-related protein 1 precursor					√			
(560)	Q12888	Tumor suppressor p53-binding protein 1								√
(561)	Q12906	Interleukin enhancer-binding factor 3					√			
(562)	Q12931	Heat shock protein 75 kDa, mitochondrial precursor								
(563)	Q13011	Delta(3,5)-Delta(2,4)-dienoyl-CoA isomerase, mitochondrial precursor					√			
(564)	Q13162	Peroxiredoxin-4					√			
(565)	Q13228	Selenium-binding protein 1								
(566)	Q13231	Chitotriosidase-1 precursor					√			
(567)	Q13283	Ras-GTPase-activating protein-binding protein 1					√			
(568)	Q13421	Mesothelin precursor								
(569)	Q13445	Transmembrane emp24 domain-containing protein 1 precursor					√			
(570)	Q13557	calcium/calmodulin-dependent protein kinase II delta isoform 1					√			
(571)	Q13614	Myotubularin-related protein 2					√			
(572)	Q13765	Nascent polypeptide-associated complex alpha subunit					√			
(573)	Q13813	Spectrin alpha chain, brain					√			
(574)	Q13835	Plakophilin 1	√			√	√			√
(575)	Q13867	Bleomycin hydrolase					√			
(576)	Q14055	Collagen alpha-2(IX) chain precursor					√			
(577)	Q14116	Interleukin-18 precursor								√
(578)	Q14134	Tripartite motif-containing protein 29					√			√
(579)	Q14166	Tubulin--tyrosine ligase-like protein 12					√			
(580)	Q14210	Lymphocyte antigen 6D precursor								√
(581)	Q14508	WAP four-disulfide core domain protein 2 precursor					√			√
(582)	Q14515	SPARC-like protein 1 precursor					√			
(583)	Q14624	Inter-alpha-trypsin inhibitor heavy chain H4 precursor				√	√			
(584)	Q14677	Clathrin interactor-1					√			
(585)	Q14764	Major vault protein					√			
(586)	Q14839	Chromodomain helicase-DNA-binding protein 4					√			
(587)	Q14914	NADP-dependent leukotriene B4 12-hydroxydehydrogenase								
(588)	Q15056	Eukaryotic translation initiation factor 4H								√
(589)	Q15075	Early endosome antigen 1					√			
(590)	Q15084	Protein disulfide-isomerase A6 precursor					√			
(591)	Q15149	Plectin-1					√			√
(592)	Q15185	Prostaglandin E synthase 3 (Cytosolic prostaglandin E2 synthase)					√			
(593)	Q15365	Poly(rC)-binding protein 1					√			
(594)	Q15424	Scaffold attachment factor B					√			
(595)	Q15459	Splicing factor 3 subunit 1					√			
(596)	Q15468	SCL-interrupting locus protein					√			
(597)	Q15555	Microtubule-associated protein RB/EB family member 2					√			
(598)	Q15642	Cdc-42 interacting protein 4					√			
(599)	Q15643	Thyroid receptor-interacting protein 11					√			
(600)	Q15651	High mobility group nucleosome-binding domain-containing protein 3								√

Accession No	Protein Description	Dasari[1]	DiQuinzio[2]	Klein[3]	Pereira[4]	Shaw[5]	Tang[6]	Venkataraman[7]	This study
(601) Q15843	NEDD8								√
(602) Q15847	Adipose most abundant gene transcript 2 protein								√
(603) Q16270	Insulin-like growth factor-binding protein 7 precursor					√			
(604) Q16363	Laminin-alpha-4 chain precursor					√			
(605) Q16537	serine/threonine protein phosphatase 2A, 56 kDa regulatory subunit, epsilon isoform				√				
(606) Q16610	Extracellular matrix protein 1 precursor	√			√	√			√
(607) Q16629	Splicing factor, arginine/serine-rich 7								√
(608) Q16695	Histone H3.1t								√
(609) Q16825	Tyrosine-protein phosphatase non-receptor type 21								√
(610) Q1U7T2	Oligopeptide/dipeptide ABC transporter, ATP-binding protein-like						√		
(611) Q32MZ4	Leucine-rich repeat flightless-interacting protein 1					√			
(612) Q3KNS1	Patched domain-containing protein 3					√			
(613) Q3KQU3	MAP7 domain-containing protein 1								√
(614) Q3MII2	Serine proteinase inhibitor						√		
(615) Q495M9	Usher syndrome type-1G protein					√			
(616) Q4L180	GPBP-interacting protein 130d					√			
(617) Q53FA7	Putative quinone oxidoreductase					√			
(618) Q53GZ6	Heat shock 70kDa protein 8 isoform 1						√		
(619) Q53RT3	Retroviral-like aspartic protease 1 precursor					√			
(620) Q5CZC0	Fibrous sheath-interacting protein 2								√
(621) Q5D862	Filaggrin-2 (FLG-2)					√			
(622) Q5H9J7	Protein BEX5					√			
(623) Q5QNY2	Heat shock 70kDa protein						√		
(624) Q5T0N1	Tetratricopeptide repeat-containing protein					√			
(625) Q5T0Z8	Uncharacterized protein C6orf132								√
(626) Q5T3I0	G patch domain containing 4 protein isoform 1					√			
(627) Q5TZ20	Olfactory receptor 2G6								√
(628) Q5TZA2	Rootletin								√
(629) Q5VTE0	Putative elongation factor 1-alpha-like 3								√
(630) Q5VTM1	Protein FAM25								√
(631) Q6E0U4	Dermokine precursor					√			√
(632) Q6IBS0	Twinfilin-2					√			
(633) Q6N089	Ig gamma-1 chain C region					√			
(634) Q6P2D8	X-ray radiation resistance-associated protein 1					√			
(635) Q6P3W6	Neuroblastoma breakpoint family member 10								√
(636) Q6P4A8	Putative phospholipase B-like 1 precursor					√			
(637) Q6UWN5	Ly6/PLAUR domain-containing protein 5 precursor					√			
(638) Q6UWP8	Suprabasin precursor					√			√
(639) Q6WCQ1	Myosin phosphatase-Rho interacting protein					√			
(640) Q6XPR3	Repetin								√
(641) Q6ZMR5	Transmembrane protease, serine 11A					√			
(642) Q6ZN66	Guanylate-binding protein 6					√			
(643) Q6ZVX7	Putative uncharacterized protein LOC342897					√			√

Accession No		Protein Description	Dasari[1]	DiQuinzio[2]	Klein[3]	Pereira[4]	Shaw[5]	Tang[6]	Venkataraman[7]	This study
(644)	Q71DI3	Histone H3.2								√
(645)	Q71UI9	Histone H2AV					√			
(646)	Q71UM5	40S ribosomal protein S27-like protein								√
(647)	Q7L7L0	Histone H2A type 3								√
(648)	Q7RTV2	Glutathione S-transferase A5								
(649)	Q7Z3Z4	Piwi-like protein 4					√			
(650)	Q7Z406	myosin, heavy chain 14 isoform 1					√			√
(651)	Q7Z5L0	Vitelline membrane outer layer protein 1 homolog precursor								
(652)	Q86SG5	Protein S100-A7-like 1					√			√
(653)	Q86T26	Transmembrane protease, serine 11B					√			
(654)	Q86UP2	Kinectin					√			
(655)	Q86VD1	MORC family CW-type zinc finger 1					√			
(656)	Q86XP0	Cytosolic phospholipase A2 delta					√			
(657)	Q86YZ3	Hornerin					√			
(658)	Q8IUE6	Histone H2A type 2-B								√
(659)	Q8IUS5	Abhydrolase domain-containing protein 7								√
(660)	Q8IVV2	Lipoxygenase homology domain-containing protein 1								√
(661)	Q8IW41	MAP kinase-activated protein kinase 5								
(662)	Q8IY18	SMC5 structural maintenance of chromosomes 5-like 1					√			
(663)	Q8IY33	MICAL-like protein 2					√			
(664)	Q8IZQ1	WD repeat and FYVE domain-containing protein 3								√
(665)	Q8N0V4	Leucine-rich repeat LGI family member 2 precursor					√			
(666)	Q8N1A0	Keratin-like protein KRT222								√
(667)	Q8N257	Histone H2B type 3-B								√
(668)	Q8N2Z9	Centromere protein S					√			
(669)	Q8N355	IGLC1 protein					√			
(670)	Q8N4F0	Bactericidal/permeability-increasing protein-like 1 precursor								
(671)	Q8N568	Serine/threonine-protein kinase DCAMKL2					√			
(672)	Q8N6Q3	CD177 antigen precursor					√			
(673)	Q8N7U6	EF-hand domain-containing family member B					√			
(674)	Q8NA31	Coiled-coil domain-containing protein 79								√
(675)	Q8NAC3	Interleukin-17 receptor C precursor								√
(676)	Q8NBI6	Protein C3orf21					√			
(677)	Q8NC51	Plasminogen activator inhibitor 1 RNA-binding protein					√			
(678)	Q8NCB2	CaM kinase-like vesicle-associated protein					√			
(679)	Q8NCR0	UDP-GalNAc:beta-1,3-N-acetylgalactosaminyltransferase 2								√
(680)	Q8NFC6	Protein FAM44A					√			
(681)	Q8NGC9	Olfactory receptor 11H4								√
(682)	Q8NHM4	Putative trypsin-6								√
(683)	Q8NHS3	Major facilitator superfamily domain-containing protein 8								√
(684)	Q8TC20	Cancer-associated gene 1 protein								√
(685)	Q8TD31	Coiled-coil alpha-helical rod protein 1								√
(686)	Q8TDC3	BR serine/threonine-protein kinase 1					√			

Accession No		Protein Description	Dasari[1]	DiQuinzio[2]	Klein[3]	Pereira[4]	Shaw[5]	Tang[6]	Venkataraman[7]	This study
(687)	Q8TDL5	Long palate, lung and nasal epithelium carcinoma associated protein 1	√		√	√				
(688)	Q8TE68	Epidermal growth factor receptor kinase substrate 8-like protein 1					√			
(689)	Q8TER0	Sushi, nidogen and EGF-like domain-containing protein 1								√
(690)	Q8TER5	Protein SOLO								√
(691)	Q8WV44	Tripartite motif protein 41					√			
(692)	Q8WVV4	Premature ovarian failure, 1B					√			
(693)	Q8WW22	DnaJ homolog subfamily A member 4					√			
(694)	Q8WWI1	LIM domain only protein 7					√			√
(695)	Q8WWY7	WAP four-disulfide core domain protein 12 precursor					√			
(696)	Q8WXH0	Nesprin-2								√
(697)	Q8WXI7	Mucin-16					√			
(698)	Q8WXX0	Ciliary dynein heavy chain 7					√			
(699)	Q8WYL5	Protein phosphatase Slingshot homolog 1					√			
(700)	Q8WYP5	transcription factor ELYS					√			
(701)	Q8WZ42	Titin					√			
(702)	Q92597	Protein NDRG1					√			√
(703)	Q92614	Myosin-XVIIIa (Myosin containing a PDZ domain)					√			
(704)	Q92736	Ryanodine recpetor 2					√			
(705)	Q92765	Secreted frizzled-related protein 3 precursor								√
(706)	Q92817	Envoplakin	√				√			√
(707)	Q92820	Gamma-glutamyl hydrolase precursor					√			
(708)	Q92876	Kallikrein-6 precursor					√			√
(709)	Q93077	Histone H2A type 1-C								√
(710)	Q93100	Phosphorylase b kinase regulatory subunit beta								√
(711)	Q96BT7	Alkylated DNA repair protein alkB homolog 8					√			
(712)	Q96C19	EF-hand domain-containing protein 2					√			
(713)	Q96C86	Scavenger mRNA decapping enzyme DcpS					√			
(714)	Q96CS3	UBX domain-containing protein 8					√			
(715)	Q96F07	cytoplasmic FMR1 interacting protein 2					√			
(716)	Q96FF9	Sororin								√
(717)	Q96FQ6	Protein S100-A16								√
(718)	Q96FX8	p53 apoptosis effector related to PMP-22					√			
(719)	Q96HC4	PDZ and LIM domain protein 5								√
(720)	Q96HE7	ERO1-like protein alpha precursor					√			√
(721)	Q96JD0	Amyloid lambda 6 light chain variable region SAR					√			
(722)	Q96JY6	PDZ and LIM domain protein 2					√			
(723)	Q96KC8	DnaJ homolog subfamily C member 1				√				
(724)	Q96P63	Serpin B12					√			√
(725)	Q96PQ0	VPS10 domain-containing receptor SorCS2 precursor					√			
(726)	Q96QH2	PML-RARA-regulated adapter molecule 1 (PRAM-1) (PRAM)					√			
(727)	Q96QV6	Histone H2A.1A								
(728)	Q96RM1	Small-proline-rich protein 2F								
(729)	Q96RY5	Protein cramped-like					√			

Accession No	Protein Description	Dasari[1]	DiQuinzio[2]	Klein[3]	Pereira[4]	Shaw[5]	Tang[6]	Venkataraman[7]	This study
(730) Q96S94	Cyclin-L2					√			
(731) Q96TA1	Niban-like protein					√			
(732) Q99102	mucin 4 isoform a					√			
(733) Q99497	DJ-1 protein				√				
(734) Q99523	Sortilin precursor					√			
(735) Q99538	Legumain precursor					√			
(736) Q99835	Smoothened homolog precursor								√
(737) Q99877	Histone H2B type 1-N								√
(738) Q99880	Histone H2B.c	√						√	√
(739) Q9BPY8	Homeodomain-only protein					√			
(740) Q9BQE3	Tubulin alpha-1C chain								√
(741) Q9BQR3	Serine protease 27 precursor					√			
(742) Q9BRA2	Thioredoxin-like protein 5					√			
(743) Q9BS26	Thioredoxin domain-containing protein 4 precursor					√			
(744) Q9BW04	specifically androgen-regulated protein					√			√
(745) Q9BXL7	Caspase recruitment domain-containing protein 11					√			
(746) Q9BYB0	proline-rich synapse-associated protein 2 isoform 1					√			
(747) Q9BYE4	Small proline-rich protein 2G								√
(748) Q9BYT8	Neurolysin, mitochondrial								√
(749) Q9BZA7	Protocadherin-11 X-linked precursor					√			
(750) Q9C0A6	SET domain-containing protein 5								√
(751) Q9C0I9	Leucine-rich repeat-containing protein 27					√			
(752) Q9GZP4	UPF0424 protein C1orf128					√			
(753) Q9GZV4	Eukaryotic translation initiation factor 5A-2								√
(754) Q9H008	Phospholysine phosphohistidine inorganic pyrophosphate phosphatase					√			
(755) Q9H0W9	Ester hydrolase C11orf54					√			
(756) Q9H1E1	Ribonuclease 7 precursor					√			√
(757) Q9H201	Epsin-3					√			
(758) Q9H251	Cadherin related 23					√			
(759) Q9H361	Polyadenylate-binding protein 3					√			
(760) Q9H3S7	Tyrosine-protein phosphatase non-receptor type 23					√			
(761) Q9H4M9	EH-domain-containing protein 1					√			
(762) Q9H5V8	CUB domain-containing protein 1					√			
(763) Q9H6S3	Epidermal growth factor receptor kinase substrate 8-like protein 2					√			
(764) Q9H7D7	WD repeat-containing protein 26								√
(765) Q9HAY6	Beta,beta-carotene 15,15'-monooxygenase								√
(766) Q9HC84	Mucin-5B precursor	√			√	√			√
(767) Q9HCE3	Zinc finger protein 532					√			
(768) Q9HCY8	S100A14								
(769) Q9HD89	Resistin precursor					√			
(770) Q9NQ38	Serine protease inhibitor Kazal-type 5	√			√	√			√
(771) Q9NR45	Sialic acid synthase					√			
(772) Q9NRL2	Bromodomain adjacent to zinc finger domain protein 1A					√			

Accession No		Protein Description	Dasari[1]	DiQuinzio[2]	Klein[3]	Pereira[4]	Shaw[5]	Tang[6]	Venkataraman[7]	This study
(773)	Q9NS15	latent transforming growth factor beta binding protein 3					√			
(774)	Q9NSY1	BMP-2-inducible protein kinase					√			
(775)	Q9NU02	Ankyrin repeat domain-containing protein 5					√			
(776)	Q9NX62	Inositol monophosphatase 3					√			
(777)	Q9NYK1	Toll-like receptor 7 precursor								√
(778)	Q9NYQ8	Protocadherin Fat 2 precursor					√			
(779)	Q9NZH8	Interleukin-1 family member 9					√			
(780)	Q9NZQ8	Transient receptor potential cation channel, subfamily M, member 5					√			
(781)	Q9NZT1	Calmodulin-like protein 5					√			√
(782)	Q9P0G3	Kallikrein-14								√
(783)	Q9P1Z0	Zinc finger and BTB domain-containing protein 4					√			
(784)	Q9P202	Whirlin					√			
(785)	Q9P258	Protein RCC2					√			
(786)	Q9UBC9	Small proline-rich protein 3	√		√	√	√			√
(787)	Q9UBD6	Rhesus-associated C glycoprotein					√			
(788)	Q9UBG3	Cornulin					√			√
(789)	Q9UBR2	Cathepsin Z precursor					√			
(790)	Q9UBX7	Kallikrein 11 precursor	√			√	√			√
(791)	Q9UFN0	Protein NipSnap3A					√			
(792)	Q9UGL9	NICE-1 protein	√			√				
(793)	Q9UGM3	deleted in malignant brain tumors 1 isoform c precursor					√			
(794)	Q9UGV6	High mobility group protein 1-like 10				√				
(795)	Q9UH77	Kelch-like protein 3					√			
(796)	Q9UHG3	Prenylcysteine oxidase precursor					√			
(797)	Q9UHL4	Dipeptidyl-peptidase 2 precursor					√			
(798)	Q9UI42	Carboxypeptidase A4 precursor					√			
(799)	Q9UIV8	Serpin B13					√			√
(800)	Q9UJY1	Heat shock protein beta-8					√			√
(801)	Q9UKR0	Kallikrein-12 precursor					√			
(802)	Q9UKR3	Kallikrein 13 precursor	√			√	√			√
(803)	Q9UL16	Coiled-coil domain-containing protein 19					√			
(804)	Q9UL52	Transmembrane protease, serine 11E	√				√			√
(805)	Q9ULH0	Ankyrin repeat-rich membrane spanning protein					√			
(806)	Q9ULH4	Leucine-rich repeat and fibronectin type-III domain-containing protein 2 precursor					√			
(807)	Q9ULV0	Myosin-5B					√			√
(808)	Q9UM54	Myosin-6					√			
(809)	Q9UN36	Protein NDRG2								
(810)	Q9UNZ2	NSFL1 cofactor p47					√			
(811)	Q9UPP5	KIAA1107 protein					√			
(812)	Q9UPQ7	PDZ domain-containing RING finger protein 3								√
(813)	Q9UPT5	Exocyst complex component 7					√			
(814)	Q9UPY3	dicer1					√			
(815)	Q9Y275	Tumor necrosis factor ligand superfamily member 13B								

Accession No		Protein Description	Dasari[1]	DiQuinzio[2]	Klein[3]	Pereira[4]	Shaw[5]	Tang[6]	Venkataraman[7]	This study
(816)	Q9Y285	Phenylalanyl-tRNA synthetase alpha chain					√			
(817)	Q9Y2B0	MIR-interacting saposin-like protein precursor					√			
(818)	Q9Y2V2	Calcium-regulated heat stable protein 1								√
(819)	Q9Y3T6	R3H and coiled-coil domain-containing protein 1								√
(820)	Q9Y446	Plakophilin-3								√
(821)	Q9Y490	Talin-1				√	√			
(822)	Q9Y4K1	Absent in melanoma 1 protein					√			√
(823)	Q9Y5Y6	Suppressor of tumorigenicity protein 14					√			
(824)	Q9Y6R7	Fc fragment of IgG binding protein					√			
(825)	Q9Y6U3	Adseverin								
(826)	Q9Y6V0	Protein piccolo (Aczonin)					√			

1. Dasari S, Pereira L, Reddy AP, Michaels JE, Lu X, Jacob T, Thomas A, Rodland M, Roberts CT, Jr., Gravett MG et al.: **Comprehensive proteomic analysis of human cervical-vaginal fluid.** *J Proteome Res* 2007, **6:**1258-1268.

2. DI Quinzio MK, Oliva K, Holdsworth SJ, Ayhan M, Walker SP, Rice GE, Georgiou HM, Permezel M: **Proteomic analysis and characterisation of human cervico-vaginal fluid proteins.** *Aust N Z J Obstet Gynaecol* 2007, **47:**9-15.

3. Klein LL, Jonscher KR, Heerwagen MJ, Gibbs RS, McManaman JL: **Shotgun proteomic analysis of vaginal fluid from women in late pregnancy.** *Reprod Sci* 2008, **15:**263-273.

4. Pereira L, Reddy AP, Jacob T, Thomas A, Schneider KA, Dasari S, Lapidus JA, Lu X, Rodland M, Roberts CT, Jr. et al.: **Identification of novel protein biomarkers of preterm birth in human cervical-vaginal fluid.** *J Proteome Res* 2007, **6:**1269-1276.

5. Shaw JL, Smith CR, Diamandis EP: **Proteomic analysis of human cervico-vaginal fluid.** *J Proteome Res* 2007, **6:**2859-2865.

6. Tang LJ, De SF, Odreman F, Venge P, Piva C, Guaschino S, Garcia RC: **Proteomic analysis of human cervical-vaginal fluids.** *J Proteome Res* 2007, **6:**2874-2883.

7. Venkataraman N, Cole AL, Svoboda P, Pohl J, Cole AM: **Cationic polypeptides are required for anti-HIV-1 activity of human vaginal fluid.** *J Immunol* 2005, **175:**7560-7567.
