# Supplementary material for: Comprehensive proteomic analysis of human cervical-vaginal fluid using colposcopy samples
Source: Proteome Sci. 2009 Apr 17;7:17. doi: 10.1186/1477-5956-7-17 (PMC2678104; doi:10.1186/1477-5956-7-17)

Additional file 9 – Classification according to cellular localisation of all proteins identified in comprehensive CVF proteomics studies so far.

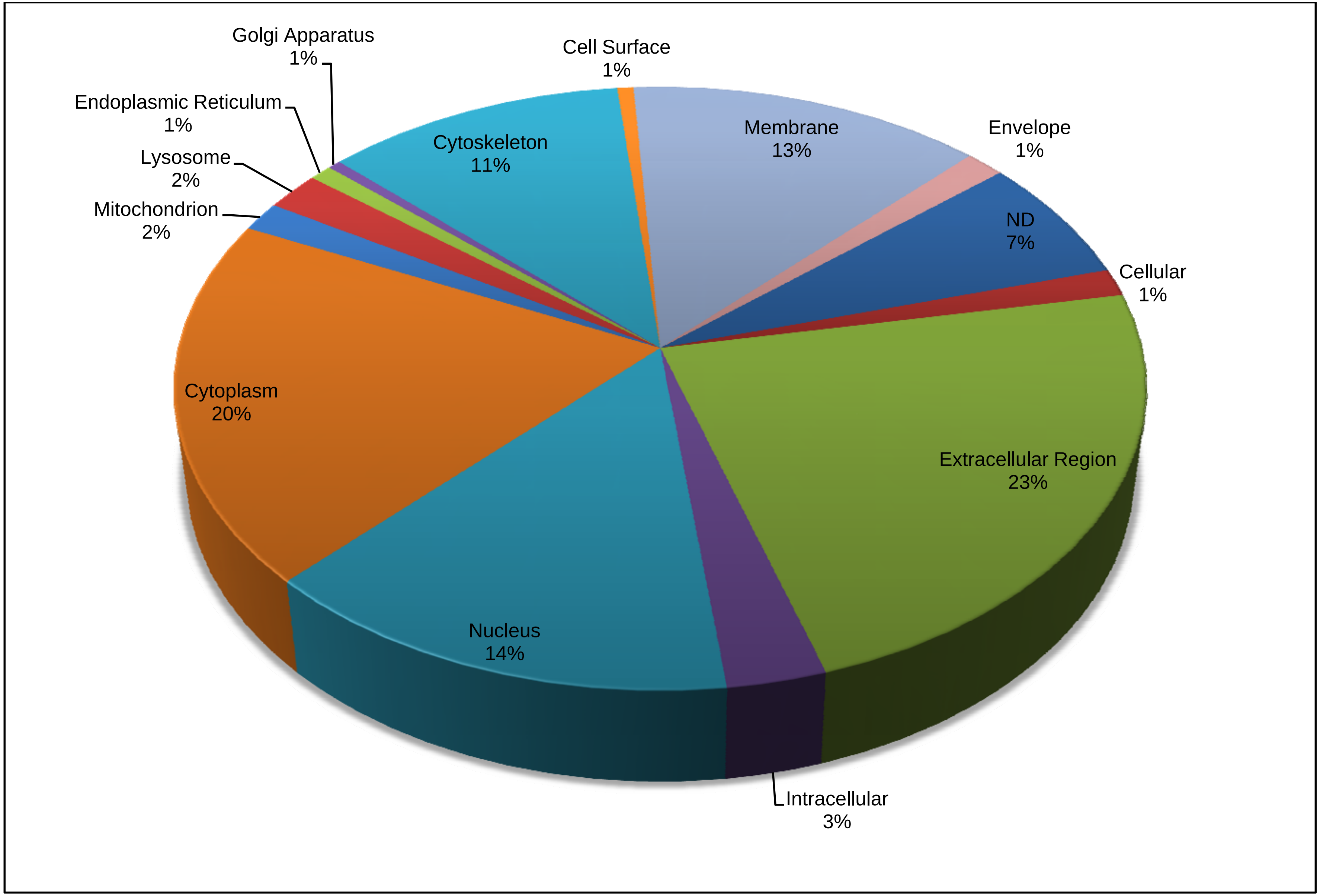

Supplement: Additional file 9 — Classification according to cellular localisation of all proteins identified in comprehensive CVF proteomics studies so far. [file 1477-5956-7-17-S9.pdf]
