# Supplementary material for: Comprehensive proteomic analysis of human cervical-vaginal fluid using colposcopy samples
Source: Proteome Sci. 2009 Apr 17;7:17. doi: 10.1186/1477-5956-7-17 (PMC2678104; doi:10.1186/1477-5956-7-17)
Supplement: Additional file 10 — Overview of proteins which were included in the overlapping protein set. [file 1477-5956-7-17-S10.pdf]

Additional file 10 – Overview of proteins which were included in the overlapping protein set.
Four studies (including ours) were compared and proteins had to be present in at least 3 of the 4 studies before they were taken up in the overlapping protein set.

Accession no		Protein description	Dasari et al.[1]	Pereira et al.[2]	Shaw et al.[3]	This study
(1)	O43707	Actinin, alpha 4	√	√	√	√
(2)	O60235	Transmembrane protease, serine 11D precursor	√	√	√	√
(3)	O60437	Periplakin	√	√	√	√
(4)	O75223	Protein C7orf24	√	√	√	
(5)	O95171	Sciellin	√	√	√	√
(6)	P00338	L-lactate dehydrogenase A chain		√	√	√
(7)	P00441	Superoxide dismutase [Cu-Zn]	√	√	√	√
(8)	P00450	Ceruloplasmin precursor	√	√	√	
(9)	P00558	Phosphoglycerate kinase 1	√	√	√	√
(10)	P00738	Haptoglobin	√	√	√	√
(11)	P01009	Alpha-1-antitrypsin precursor	√	√	√	√
(12)	P01024	Complement component 3	√	√	√	√
(13)	P01040	Cystatin A (stefin A)	√	√	√	√
(14)	P01591	Immunoglobulin J chain	√	√	√	√
(15)	P01625	Ig kappa chain V-IV region Len	√	√	√	
(16)	P01833	Polymeric immunoglobulin receptor	√	√	√	√
(17)	P01834	Ig kappa chain C region	√	√	√	√
(18)	P01842	Ig lambda chain C regions	√	√	√	√
(19)	P01857	Ig gamma-1 chain C region	√	√	√	√
(20)	P01859	Ig gamma-2 chain C region	√	√	√	√
(21)	P01861	Ig gamma-4 chain C region	√	√	√	√
(22)	P01871	Ig mu chain C region	√	√	√	
(23)	P01876	Ig alpha-1 chain C region	√	√	√	√
(24)	P01877	Ig alpha-2 chain C region	√		√	√
(25)	P02545	Lamin-A/C		√	√	√
(26)	P02647	Apolipoprotein A1	√	√	√	√
(27)	P02671	Fibrinogen alpha chain precursor	√	√	√	√
(28)	P02675	Fibrinogen beta chain precursor	√	√	√	√
(29)	P02679	Fibrinogen gamma chain	√	√	√	
(30)	P02749	Apolipoprotein H (beta-2-glycoprotein I)	√	√	√	√
(31)	P02763	Alpha-1-acid glycoprotein 1	√	√	√	√
(32)	P02765	Alpha-2-HS-glycoprotein	√	√		√
(33)	P02766	Transthyretin		√	√	√

Accession no		***Protein description***	***Dasari et al.[1]***	***Pereira et al.[2]***	***Shaw et al.[3]***	***This study***
(34)	P02768	Serum albumin precursor	√	√	√	√
(35)	P02774	Vitamin D-binding protein precursor	√	√	√	√
(36)	P02787	Serotransferrin	√	√	√	√
(37)	P02788	Lactotransferrin	√	√	√	√
(38)	P02790	Hemopexin	√	√	√	√
(39)	P03973	Antileukoproteinase 1 precursor	√	√	√	√
(40)	P04040	Catalase	√	√	√	
(41)	P04075	Fructose-bisphosphate aldolase A	√	√	√	√
(42)	P04080	Cystatin B	√	√	√	√
(43)	P04083	Annexin A1	√	√	√	√
(44)	P04406	Glyceraldehyde-3-phosphate dehydrogenase, liver	√	√	√	√
(45)	P04792	Heat-shock protein beta-1	√	√	√	√
(46)	P05109	Calgranulin A (S100A8)	√	√	√	√
(47)	P05164	Myeloperoxidase precursor	√	√	√	√
(48)	P06702	Calgranulin B (S100A9)	√	√	√	√
(49)	P06731	Carcinoembryonic antigen-related cell adhesion molecule 5 precursor	√		√	√
(50)	P06733	Alpha-enolase	√	√		√
(51)	P06753	Tropomyosin 3		√	√	√
(52)	P07108	Acyl-CoA binding protein	√	√	√	√
(53)	P07237	Protein disulfide-isomerase precursor	√	√	√	
(54)	P07355	Annexin A2	√	√	√	√
(55)	P07476	Involucrin	√	√	√	√
(56)	P07737	Profilin 1	√	√	√	√
(57)	P07858	Cathepsin B	√	√	√	
(58)	P07900	Heat shock protein HSP 90-alpha 2	√	√	√	
(59)	P08107	Heat shock 70 kDa protein 1	√	√	√	√
(60)	P08123	Collagen alpha 2 T		√	√	√
(61)	P08238	Heat shock protein HSP 90-beta		√	√	√
(62)	P08246	Leukocyte elastase precursor		√	√	√
(63)	P08311	cathepsin G	√	√	√	√
(64)	P08603	Complement factor H	√	√	√	√
(65)	P08670	Vimentin	√	√	√	√
(66)	P09211	Glutathione S-transferase P	√	√	√	√
(67)	P10599	Thioredoxin	√	√	√	√
(68)	P11021	78 kDa glucose-regulated protein	√	√	√	
(69)	P11142	Heat shock 70kDa protein 8	√	√	√	√
(70)	P12429	Annexin A3	√	√	√	√

Accession no		Protein description	Dasari et al.[1]	Pereira et al.[2]	Shaw et al.[3]	This study
(71)	P12724	Eosinophil cationic protein precursor	√	√	√	√
(72)	P13639	Elongation factor 2	√	√	√	
(73)	P13796	Lymphocyte cytosolic protein 1 (L-plastin)	√	√	√	√
(74)	P13987	CD59 glycoprotein precursor	√	√	√	√
(75)	P14618	Pyruvate kinase isozymes M1/M2	√	√	√	√
(76)	P14780	Matrix metalloproteinase-9 precursor	√	√	√	
(77)	P14923	Desmoplakin-3	√	√	√	
(78)	P15924	Desmoplakin	√	√	√	√
(79)	P16401	Histone H1.5 (Histone H1a)	√	√	√	√
(80)	P18206	Vinculin	√	√	√	√
(81)	P18510	Interleukin 1 receptor antagonist protein	√	√	√	√
(82)	P18669	Phosphoglycerate mutase 1	√	√	√	√
(83)	P20160	Azurocidin 1 (cationic antimicrobial protein 37)		√	√	√
(84)	P20810	Calpastatin (Calpain inhibitor) (Sperm BS-17 component)	√	√	√	√
(85)	P22528	Cornifin B	√	√	√	√
(86)	P22532	Small proline-rich protein 2D	√	√		√
(87)	P22735	Protein-glutamine gamma-glutamyltransferase K	√		√	√
(88)	P23528	cofilin-1	√		√	√
(89)	P24158	Myeloblastin precursor	√	√	√	√
(90)	P26038	Moesin	√	√	√	
(91)	P27482	Calmodulin-like protein 3	√	√	√	√
(92)	P28799	Granulins precursor	√	√	√	
(93)	P29373	Cellular retinoic acid-binding protein 2	√	√	√	√
(94)	P29508	Squamous cell carcinoma antigen 1 (SCCA-1); Serpin B3	√	√	√	√
(95)	P30086	Phosphatidylethanolamine-binding protein; Prostatic binding protein; neuropolypeptide h3	√	√	√	√
(96)	P30740	Monocyte/neutrophil elastase inhibitor	√	√	√	√
(97)	P31151	S100 calcium-binding protein A7 (psoriasin)	√	√	√	√
(98)	P31947	Stratifin; 14-3-3 protein sigma	√	√	√	√
(99)	P31949	S100 calcium-binding protein A11 (calgizzarine)		√	√	√
(100)	P32320	Cytidine deaminase		√	√	√
(101)	P32926	Desmoglein-3	√	√	√	√
(102)	P35321	Small-proline rich protein 1A; Cornifin A	√	√	√	√
(103)	P35326	Small proline-rich protein 2A	√	√	√	√
(104)	P35579	Myosin-9		√	√	√
(105)	P37837	Transaldolase	√	√	√	
(106)	P47929	Lectin, galactoside-binding, soluble, 7 (galectin 7)	√	√	√	√
(107)	P48594	Squamous cell carcinoma antigen 2; Serpin B4	√	√	√	√

Accession no		Protein description	Dasari et al.[1]	Pereira et al.[2]	Shaw et al.[3]	This study
(108)	P54108	Cysteine-rich secretory protein 3	√	√	√	√
(109)	P59665	Neutrophil defensin 1 precursor	√	√	√	√
(110)	P60174	Triosephosphate isomerase	√	√	√	√
(111)	P60709	Actin, cytoplasmic 1	√	√	√	√
(112)	P60903	S100 calcium binding protein A10; Calpactin I light chain		√	√	√
(113)	P61626	Lysozym C	√	√	√	√
(114)	P62805	Histone H4	√	√	√	√
(115)	P62937	Peptidyl-prolyl cis-trans isomerase A (Cyclophilin A)	√	√		√
(116)	P62988	Ubiquitin	√	√	√	√
(117)	P63104	14-3-3 protein zeta/delta	√	√		√
(118)	P67936	Tropomyosin alpha 4 chain	√	√		√
(119)	P68104	eukaryotic translation elongation factor 1 alpha 1	√		√	√
(120)	P68871	Hemoglobin beta chain		√	√	√
(121)	P69905	Hemoglobin alpha subunit		√	√	√
(122)	P80188	Neutrophil gelatinase-associated lipocalin	√	√	√	√
(123)	P80511	Protein S100-A12		√	√	√
(124)	Q01469	Fatty acid-binding protein, epidermal	√	√	√	√
(125)	Q02487	Desmocollin-2 precursor	√	√	√	√
(126)	Q06830	Peroxiredoxin 1	√	√	√	√
(127)	Q09666	Neuroblast differentiation-associated protein AHNAK	√	√	√	√
(128)	Q13835	Plakophilin 1	√	√	√	√
(129)	Q16610	Extracellular matrix protein 1 precursor	√	√	√	√
(130)	Q92817	Envoplakin	√		√	√
(131)	Q9HC84	Mucin-5B precursor	√	√	√	√
(132)	Q9NQ38	Serine protease inhibitor Kazal-type 5	√	√	√	√
(133)	Q9UBC9	Small proline-rich protein 3	√	√	√	√
(134)	Q9UBX7	Kallikrein 11 precursor	√	√	√	√
(135)	Q9UKR3	Kallikrein 13 precursor	√	√	√	√
(136)	Q9UL52	Transmembrane protease, serine 11E	√		√	√

1. Dasari S, Pereira L, Reddy AP, Michaels JE, Lu X, Jacob T, Thomas A, Rodland M, Roberts CT, Jr., Gravett MG et al.: **Comprehensive proteomic analysis of human cervical-vaginal fluid.** *J Proteome Res* 2007, **6:**1258-1268.

2. Pereira L, Reddy AP, Jacob T, Thomas A, Schneider KA, Dasari S, Lapidus JA, Lu X, Rodland M, Roberts CT, Jr. et al.: **Identification of novel protein biomarkers of preterm birth in human cervical-vaginal fluid.** *J Proteome Res* 2007, **6:**1269-1276.

3. Shaw JL, Smith CR, Diamandis EP: **Proteomic analysis of human cervico-vaginal fluid.** *J Proteome Res* 2007, **6:**2859-2865.
