# Supplementary material for: Comprehensive proteomic analysis of human cervical-vaginal fluid using colposcopy samples
Source: Proteome Sci. 2009 Apr 17;7:17. doi: 10.1186/1477-5956-7-17 (PMC2678104; doi:10.1186/1477-5956-7-17)
Supplement: Additional file 11 — Classification of proteins which were included overlapping in the protein set. [file 1477-5956-7-17-S11.pdf]

Additional file 11 – Classification of proteins which were included in the overlapping protein set.
Classification according to their functional process and cellular localization is presented.

Accession no		Protein description	Cellular localization	Functional proces
(1)	O43707	Actinin, alpha 4	Nucleus	Cell structure and mobility
(2)	O60235	Transmembrane protease, serine 11D precursor	Extracellular Region	Protein metabolism and modification
				Nucleoside, nucleotide and nucleic acid metabolism
(3)	O60437	Periplakin	Cytoskeleton	Developmental processes
				Cell proliferation and differentiation
(4)	O75223	Protein C7orf24	ND	ND
(5)	O95171	Sciellin	Cytoskeleton	Developmental processes
(6)	P00338	L-lactate dehydrogenase A chain	Cytoplasm	Carbohydrate metabolism
(7)	P00441	Superoxide dismutase [Cu-Zn]	Extracellular Region	Immunity and defense
(8)	P00450	Ceruloplasmin precursor	Extracellular Region	Homeostasis
(9)	P00558	Phosphoglycerate kinase 1	Cytoplasm	Carbohydrate metabolism
(10)	P00738	Haptoglobin	Extracellular Region	Blood circulation and gas exchange
				Protein metabolism and modification
				Immunity and defense
(11)	P01009	Alpha-1-antitrypsin precursor	Extracellular Region	Protein metabolism and modification
(12)	P01024	Complement component 3	Extracellular Region	Immunity and defense
(13)	P01040	Cystatin A (stefin A)	Cytoskeleton	Protein metabolism and modification
(14)	P01591	Immunoglobulin J chain	Extracellular Region	Immunity and defense
(15)	P01625	Ig kappa chain V-IV region Len	Extracellular Region	Immunity and defense
(16)	P01833	Polymeric immunoglobulin receptor	Membrane	Intracellular protein traffic
				Immunity and defense
(17)	P01834	Ig kappa chain C region	Extracellular Region	Immunity and defense
(18)	P01842	Ig lambda chain C regions	Membrane	Immunity and defense
(19)	P01857	Ig gamma-1 chain C region	Membrane	Immunity and defense
(20)	P01859	Ig gamma-2 chain C region	Membrane	Immunity and defense
(21)	P01861	Ig gamma-4 chain C region	Membrane	Immunity and defense
(22)	P01871	Ig mu chain C region	Membrane	Immunity and defense
(23)	P01876	Ig alpha-1 chain C region	Extracellular Region	Immunity and defense
(24)	P01877	Ig alpha-2 chain C region	Membrane	Immunity and defense

(25)	P02545	Lamin-A/C	Nucleus	Cell structure and mobility
(26)	P02647	Apolipoprotein A1	Extracellular Region	Lipid, fatty acid and steroid metabolism
				Transport
(27)	P02671	Fibrinogen alpha chain precursor	Extracellular Region	Cell proliferation and differentiation
				Blood circulation and gas exchange
				Immunity and defense
(28)	P02675	Fibrinogen beta chain precursor	Extracellular Region	Immunity and defense
				Blood circulation and gas exchange
				Cell proliferation and differentiation
(29)	P02679	Fibrinogen gamma chain	Extracellular Region	Blood circulation and gas exchange
				Immunity and defense
				Cell proliferation and differentiation
(30)	P02749	Apolipoprotein H (beta-2-glycoprotein I)	Extracellular Region	Immunity and defense
(31)	P02763	Alpha-1-acid glycoprotein 1	Extracellular Region	Immunity and defense
(32)	P02765	Alpha-2-HS-glycoprotein	Extracellular Region	Immunity and defense
				Developmental processes
				Protein metabolism and modification
(33)	P02766	Transthyretin	Extracellular Region	Transport
(34)	P02768	Serum albumin precursor	Extracellular Region	Transport
(35)	P02774	Vitamin D-binding protein precursor	Extracellular Region	Coenzyme and prosthetic group metabolism
				Transport
(36)	P02787	Serotransferrin	Extracellular Region	Transport
				Miscellaneous
(37)	P02788	Lactotransferrin	Extracellular Region	Miscellaneous
				Transport
(38)	P02790	Hemopexin	Extracellular Region	Transport
				Coenzyme and prosthetic group metabolism
(39)	P03973	Antileukoproteinase 1 precursor	Extracellular Region	Immunity and defense
				Protein metabolism and modification
(40)	P04040	Catalase	Membrane	Immunity and defense
				Electron transport
(41)	P04075	Fructose-bisphosphate aldolase A	ND	Carbohydrate metabolism
(42)	P04080	Cystatin B	Extracellular Region	Protein metabolism and modification

(43)	P04083	Annexin A1	Cytoskeleton	Cell structure and mobility
				Lipid, fatty acid and steroid metabolism
				Signal transduction
(44)	P04406	Glyceraldehyde-3-phosphate dehydrogenase, liver	Cytoplasm	Carbohydrate metabolism
(45)	P04792	Heat-shock protein beta-1	Cytoskeleton	Protein metabolism and modification
				Immunity and defense
(46)	P05109	Calgranulin A (S100A8)	Extracellular Region	Cell structure and mobility
				Immunity and defense
(47)	P05164	Myeloperoxidase precursor	Lysosome	Immunity and defense
(48)	P06702	Calgranulin B (S100A9)	Extracellular Region	Signal transduction
				Immunity and defense
(49)	P06731	Carcinoembryonic antigen-related cell adhesion molecule 5 precursor	Membrane	Signal transduction
				Cell adhesion
(50)	P06733	Alpha-enolase	Nucleus	Carbohydrate metabolism
(51)	P06753	Tropomyosin 3	Cytoskeleton	Developmental processes
				Muscle contraction
				Cell structure and mobility
(52)	P07108	Acyl-CoA binding protein	ND	Lipid, fatty acid and steroid metabolism
(53)	P07237	Protein disulfide-isomerase precursor	Extracellular Region	Protein metabolism and modification
(54)	P07355	Annexin A2	Membrane	Intracellular protein traffic
				Developmental processes
				Cell structure and mobility
(55)	P07476	Involucrin	Cytoskeleton	Cell structure and mobility
				Cell proliferation and differentiation
(56)	P07737	Profilin 1	Cytoskeleton	Nucleoside, nucleotide and nucleic acid metabolism
				Developmental processes
				Cell adhesion
(57)	P07858	Cathepsin B	Intracellular	Protein metabolism and modification
				Oncogenesis
(58)	P07900	Heat shock protein HSP 90-alpha 2	Cytoplasm	Immunity and defense
				Protein metabolism and modification
(59)	P08107	Heat shock 70 kDa protein 1	Mitochondrion	Protein metabolism and modification
				Immunity and defense

(60)	P08123	Collagen alpha 2 T	Extracellular Region	Cell adhesion
				Cell structure and mobility
(61)	P08238	Heat shock protein HSP 90-beta	Cytoplasm	Protein metabolism and modification
				Immunity and defense
(62)	P08246	Leukocyte elastase precursor	Extracellular Region	Immunity and defense
				Protein metabolism and modification
(63)	P08311	cathepsin G	Lysosome	Immunity and defense
				Apoptosis
				Protein metabolism and modification
(64)	P08603	Complement factor H	Extracellular Region	Immunity and defense
(65)	P08670	Vimentin	Cytoskeleton	Developmental processes
				Cell structure and mobility
(66)	P09211	Glutathione S-transferase P	Cytoplasm	Immunity and defense
(67)	P10599	Thioredoxin	Cytoplasm	Immunity and defense
				Other metabolism
				Electron transport
				Signal transduction
				Apoptosis
				Cell proliferation and differentiation
(68)	P11021	78 kDa glucose-regulated protein	Endoplasmic Reticulum	Developmental processes
				Apoptosis
(69)	P11142	Heat shock 70kDa protein 8	Cell Surface	Immunity and defense
				Protein metabolism and modification
(70)	P12429	Annexin A3	Cytoplasm	Lipid, fatty acid and steroid metabolism
(71)	P12724	Eosinophil cationic protein precursor	Extracellular Region	Nucleoside, nucleotide and nucleic acid metabolism
(72)	P13639	Elongation factor 2	Cytoplasm	Protein metabolism and modification
(73)	P13796	Lymphocyte cytosolic protein 1 (L-plastin)	Cytoplasm	Cell structure and mobility
(74)	P13987	CD59 glycoprotein precursor	Membrane	Immunity and defense
(75)	P14618	Pyruvate kinase isozymes M1/M2	Cytoplasm	Carbohydrate metabolism
(76)	P14780	Matrix metalloproteinase-9 precursor	Extracellular Region	Protein metabolism and modification
(77)	P14923	Desmoplakin-3	Membrane	Signal transduction
(78)	P15924	Desmoplakin	Cytoskeleton	Cell proliferation and differentiation
				Protein metabolism and modification

				Developmental processes
(79)	P16401	Histone H1.5 (Histone H1a)	Intracellular	Nucleoside, nucleotide and nucleic acid metabolism
(80)	P18206	Vinculin	Membrane	Cell structure and mobility
(81)	P18510	Interleukin 1 receptor antagonist protein	Extracellular Region	Signal transduction
				Immunity and defense
(82)	P18669	Phosphoglycerate mutase 1	Cytoplasm	Carbohydrate metabolism
(83)	P20160	Azurocidin 1 (cationic antimicrobial protein 37)	Extracellular Region	Immunity and defense
				Protein metabolism and modification
(84)	P20810	Calpastatin (Calpain inhibitor) (Sperm BS-17 component)	Cytoplasm	Protein metabolism and modification
(85)	P22528	Cornifin B	Cytoskeleton	Protein metabolism and modification
				Developmental processes
				Cell proliferation and differentiation
(86)	P22532	Small proline-rich protein 2D	Cytoskeleton	Cell proliferation and differentiation
				Developmental processes
(87)	P22735	Protein-glutamine gamma-glutamyltransferase K	Cytoskeleton	Developmental processes
				Protein metabolism and modification
(88)	P23528	cofilin-1	Nucleus	Cell structure and mobility
(89)	P24158	Myeloblastin precursor	Cellular	Protein metabolism and modification
(90)	P26038	Moesin	Cytoskeleton	Cell structure and mobility
(91)	P27482	Calmodulin-like protein 3	ND	Cell cycle
				Cell proliferation and differentiation
				Signal transduction
(92)	P28799	Granulins precursor	Extracellular Region	Signal transduction
(93)	P29373	Cellular retinoic acid-binding protein 2	Cytoplasm	Coenzyme and prosthetic group metabolism
				Lipid, fatty acid and steroid metabolism
				Signal transduction
				Transport
				Developmental processes
(94)	P29508	Squamous cell carcinoma antigen 1 (SCCA-1); Serpin B3	Extracellular Region	Protein metabolism and modification
(95)	P30086	Prostatic binding protein	Cytoplasm	Signal transduction
(96)	P30740	Monocyte/neutrophil elastase inhibitor	Cytoplasm	Protein metabolism and modification
(97)	P31151	S100 calcium-binding protein A7 (psoriasin)	Extracellular Region	Developmental processes
				Cell proliferation and differentiation

				Immunity and defense
(98)	P31947	Stratifin; 14-3-3 protein sigma	Extracellular Region	Signal transduction<br>Cell cycle<br>Protein targeting and localization
(99)	P31949	S100 calcium-binding protein A11 (calgizzarine)	Nucleus	Nucleoside, nucleotide and nucleic acid metabolism<br>Oncogenesis<br>Cell cycle
(100)	P32320	Cytidine deaminase	Extracellular Region	Nucleoside, nucleotide and nucleic acid metabolism
(101)	P32926	Desmoglein-3	Membrane	Cell adhesion<br>Signal transduction
(102)	P35321	Small-proline rich protein 1A; Cornifin A	Cytoskeleton	Cell proliferation and differentiation<br>Developmental processes<br>Protein metabolism and modification
(103)	P35326	Small proline-rich protein 2A	Cytoskeleton	Developmental processes<br>Cell proliferation and differentiation
(104)	P35579	Myosin-9	Cytoskeleton	Developmental processes<br>Cell structure and mobility
(105)	P37837	Transaldolase	Cytoplasm	Carbohydrate metabolism
(106)	P47929	Lectin, galactoside-binding, soluble, 7 (galectin 7)	Extracellular Region	Apoptosis<br>Cell adhesion<br>Immunity and defense
(107)	P48594	Squamous cell carcinoma antigen 2; Serpin B4	Cytoplasm	Protein metabolism and modification
(108)	P54108	Cysteine-rich secretory protein 3	Extracellular Region	Developmental processes
(109)	P59665	Neutrophil defensin 1 precursor	Extracellular Region	Immunity and defense
(110)	P60174	Triosephosphate isomerase	Cytoplasm	Nucleoside, nucleotide and nucleic acid metabolism<br>Carbohydrate metabolism
(111)	P60709	Actin, cytoplasmic 1	Cytoskeleton	Cell structure and mobility<br>Transport<br>Intracellular protein traffic<br>Cell cycle
(112)	P60903	S100 calcium binding protein A10; Calpactin I light chain	ND	Developmental processes
(113)	P61626	Lysozym C	Extracellular Region	Carbohydrate metabolism<br>Immunity and defense

(114)	P62805	Histone H4	Nucleus	Nucleoside, nucleotide and nucleic acid metabolism
(115)	P62937	Peptidyl-prolyl cis-trans isomerase A (Cyclophilin A)	Extracellular Region	Protein metabolism and modification
				Immunity and defense
				Intracellular protein traffic
(116)	P62988	Ubiquitin	Nucleus	Protein metabolism and modification
(117)	P63104	14-3-3 protein zeta/delta	Cytoplasm	Cell cycle
				Signal transduction
				Protein targeting and localization
(118)	P67936	Tropomyosin alpha 4 chain	Cytoskeleton	Muscle contraction
				Cell structure and mobility
				Developmental processes
(119)	P68104	eukaryotic translation elongation factor 1 alpha 1	Cytoplasm	Protein metabolism and modification
(120)	P68871	Hemoglobin beta chain	Cytoplasm	Transport
				Other metabolism
(121)	P69905	Hemoglobin alpha subunit	Cytoplasm	Transport
				Blood circulation and gas exchange
(122)	P80188	Neutrophil gelatinase-associated lipocalin	Cytoplasm	Oncogenesis
				Immunity and defense
				Transport
(123)	P80511	Protein S100-A12	Cytoplasm	Immunity and defense
(124)	Q01469	Fatty acid-binding protein, epidermal	Cytoplasm	Transport
				Developmental processes
				Signal transduction
				Coenzyme and prosthetic group metabolism
				Lipid, fatty acid and steroid metabolism
(125)	Q02487	Desmocollin-2 precursor	Cytoskeleton	Signal transduction
				Cell adhesion
(126)	Q06830	Peroxiredoxin 1	Cytoplasm	Immunity and defense
(127)	Q09666	Neuroblast differentiation-associated protein AHNAK	Nucleus	Developmental processes
(128)	Q13835	Plakophilin 1	Cytoskeleton	Signal transduction
				Cell adhesion
(129)	Q16610	Extracellular matrix protein 1 precursor	Extracellular Region	Signal transduction
(130)	Q92817	Envoplakin	Cytoskeleton	Developmental processes

				Cell adhesion
				Cell structure and mobility
(131)	Q9HC84	Mucin-5B precursor	Extracellular Region	Developmental processes
(132)	Q9NQ38	Serine protease inhibitor Kazal-type 5	Extracellular Region	Homeostasis
(133)	Q9UBC9	Small proline-rich protein 3	Envelope	Cell proliferation and differentiation
				Developmental processes
(134)	Q9UBX7	Kallikrein 11 precursor	Extracellular Region	Cell proliferation and differentiation
				Developmental processes
				Protein metabolism and modification
				Cell cycle
(135)	Q9UKR3	Kallikrein 13 precursor	Extracellular Region	Protein metabolism and modification
(136)	Q9UL52	Transmembrane protease, serine 11E	Membrane	Protein metabolism and modification
